# Efficient construction of the hexacyclic ring core of palau'amine: the p*K*_a_ concept for proceeding with unfavorable equilibrium reactions†

**DOI:** 10.1039/d1sc03260g

**Published:** 2021-08-11

**Authors:** Eisaku Ohashi, Sangita Karanjit, Atsushi Nakayama, Kohei Takeuchi, Sherif E. Emam, Hidenori Ando, Tatsuhiro Ishida, Kosuke Namba

**Affiliations:** Graduate School of Pharmaceutical Sciences, Tokushima University 1-78 Shomachi Tokushima 770-8505 Japan namba@tokushima-u.ac.jp; Research Cluster on “Innovative Chemical Sensing”, Tokushima University 1-78 Shomachi Tokushima 770-8505 Japan

## Abstract

Palau'amine has received a great deal of attention as an attractive synthetic target due to its intriguing molecular architecture and significant immunosuppressive activity, and we achieved its total synthesis in 2015. However, the synthesized palau'amine has not been readily applicable to the mechanistic study of immunosuppressive activity, because it requires 45 longest linear steps from a commercially available compound. Here, we report the short-step construction of the ABCDEF hexacyclic ring core of palau'amine. The construction of the CDE tricyclic ring core in a single step is achieved by our p*K*_a_ concept for proceeding with unfavorable equilibrium reactions, and a palau'amine analog without the aminomethyl and chloride groups is synthesized in 20 longest linear steps from the same starting material. The palau'amine analog is confirmed to retain the immunosuppressive activity. The present synthetic approach for a palau'amine analog has the potential for use in the development of palau'amine probes for mechanistic elucidation.

## Abstract 2

A palau'amine analog (**2**) was synthesized from 2-cyclopentenone in 20 steps. The construction of the CDE tricyclic ring core in a single step is achieved by our p*K*_a_ concept for proceeding with the unfavorable equilibrium reactions.

## Graphical abstract

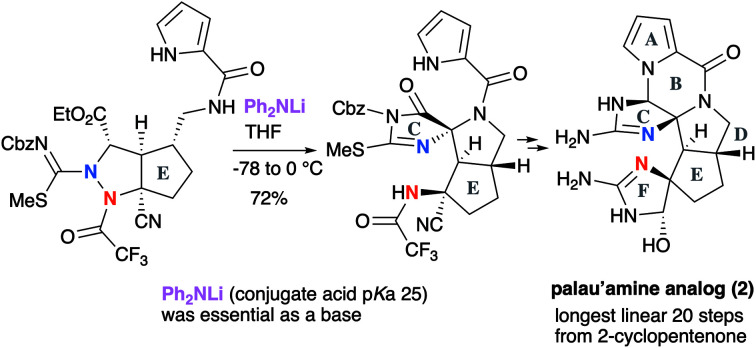

## Introduction

Pyrrole–imidazole alkaloids, which comprise a large family of natural products, have received a great deal of attention because of their potent biological activities and tremendous structural diversity.^1^ In 1993, palau'amine (**1**) was originally isolated from a sponge, Stylotella agminate, by Scheuer as a novel class of pyrrole–imidazole alkaloids,^2^ and its proposed structure was revised in 2007.^3^ Since its discovery, **1** has been an attractive synthetic target due to its intriguing molecular architecture and significant biological properties; these include antifungal, antitumor, and immunosuppressive activities, with the immunosuppressive activities being of particular interest to researchers. At least two studies reported on the mode of action of **1**.^4^ Thus, the development of molecular probes based on **1** is required for further elucidation of the potential of palau'amine as an immunosuppressive agent, and investigation into its structure–activity relationship (SAR) is also needed to drive it forward from the stage of a novel lead compound to that of a useful immunosuppressive agent. However, palau'amine is well known as a highly challenging synthetic target. Its noteworthy structural features include two guanidine moieties, a fused polycyclic system containing a spiro-cycle, a fully substituted complex cyclopentane ring, eight contiguous stereogenic centers including a nitrogen-substituted quaternary carbon center, and the *trans*-azabicyclo[3.3.0]octane skeleton (D/E ring junction). Not surprisingly, many attempts to synthesize palau'amine^3*e*,5^ and related compounds^6^ have been reported, and numerous reviews of these different approaches have been published.^7^ Nonetheless, to date, there have been only two examples of the total synthesis of **1**. Baran's group reported the first total synthesis in 2010,^8^ which was followed by the development of an asymmetric version in 2011,^9^ and our group also achieved a total synthesis in 2015.^10^

**Fig. 1:**
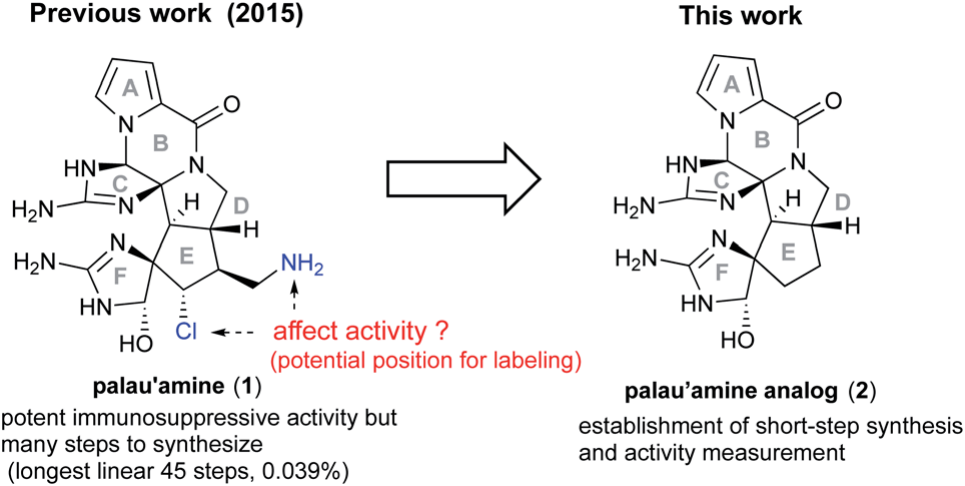
Structure of palau'amine **1** and target palau'amine analog **2**.

The key feature of our total synthesis was that a unique polycyclic ring system including a *trans*-azabicyclo[3.3.0]octane skeleton of **1** was constructed in advance and later converted to **1** by functional group transformations. This kind of approach is useful for elucidating the pharmacophore and for the development of palau'amine-based probes. We actually focused on the effects of the aminomethyl and chloride groups on the immunosuppressive activity. These functional groups were naturally considered significant for the activity, because they may function as the nucleophilic and electrophilic groups for the specific functional group of the target protein. However, if the rigid polycyclic structure containing cyclic guanidino groups was more significant for the activity and these functional groups have little effect, the aminomethyl group can serve as a potential position for labeling to develop palau'amine-based probes. In addition, the unstable chloride group, which makes synthesis and handling difficult, can be removed from probes. Thus, to elucidate the effect of these functional groups on the immunosuppressive activity, it was required to synthesize analog **2**, in which the aminomethyl and chloride groups of **1** were removed, and evaluate its activity (Fig. 1). However, our previous synthetic route to **1** required too many steps (45 longest linear steps) to expand to SAR studies. Therefore, to realistically drive the mode of action study of **1** forward, further dramatic evolution of the construction method for the ABCDEF hexa-cyclic ring core was required. In the present study, we develop a new method for short-step construction of the ABCDEF hexa-cyclic ring core and evaluate the immunosuppressive activity of **2**.

## Results and discussion

### Plan for the second-generation construction of a polycyclic ring core

**Fig. 2:**
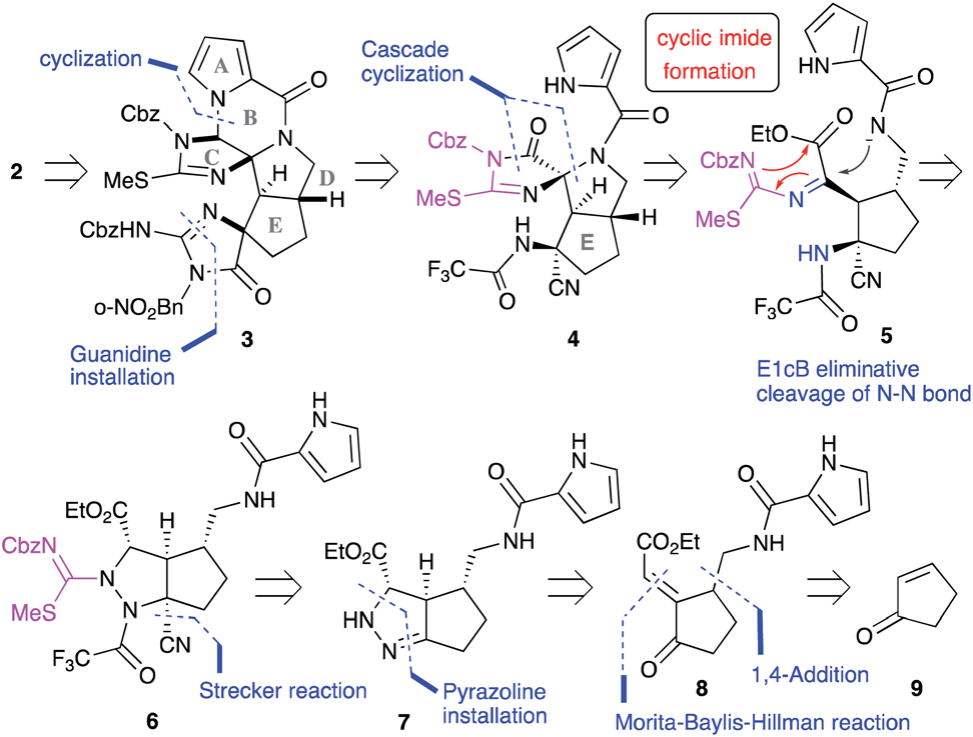
Synthetic plan of palau'amine analog **2**.

Our first-generation total synthesis required many steps to construct the C and F rings after the construction of the ABDE tetracyclic ring core. Thus, we designed a new synthetic route to construct the C and F rings efficiently. In particular, the C-ring would be constructed in a single step as follows (Fig. 2): **2** would be obtained by the functional group transformations of hexacyclic ring core **3**. The B and F rings of **3** would be constructed from **4** by the reduction of the imide moiety on the C ring and the introduction of urea into the aminonitrile moiety, respectively. The CDE tricyclic ring core **4** would be formed by a cascade cyclization reaction of **6**, in which the N–N bond cleavage giving **5** followed by the nucleophilic addition of an amide anion would form the D ring. In this substrate, subsequent nucleophilic addition would have also occurred from the isothiourea group to form the C ring. Thus, the cascade reaction of **6** could construct the C ring in a single step, in contrast to our previous C ring formation which required many steps. Since the formation of cyclic imides basically requires harsh conditions, construction of the C ring in this cascade reaction could be a challenging task. Moreover, cyclization from the carbamate-protected nitrogen of isothiourea, which has a very weak nucleophilicity, was anticipated to be particularly difficult. The short-step preparation of the cascade reaction precursor **6** is also key to the efficient synthesis of **2**. The tetra-substituted carbon center of **6** would be constructed by the Strecker reaction of the pyrazoline **7**. The pyrazoline ring of **7** would be constructed in one step by the 1,4-addition of hydrazine to enone **8** and hydrazone formation. The side chains of **8** would be introduced by the 1,4-addition and the Morita–Baylis–Hillman reaction of 2-cyclopenten-1-one **9**.

### Synthesis of the cascade cyclization precursor **6**

**Scheme 1:**
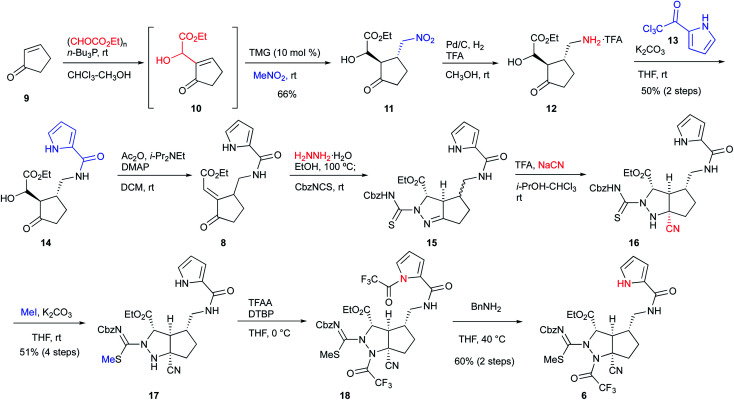
Synthesis of the cascade reaction precursor.

We started the synthesis of the cyclization precursor **6** with 2-cyclopenten-1-one **9**, which was the same starting material as that for the first-generation synthesis. The Morita–Baylis–Hillman reaction of **9** with ethyl glyoxylate afforded **10**,^10,11^ and subsequent direct addition of nitromethane and tetramethylguanidine (TMG) to the reaction mixture induced the 1,4-addition reaction of nitromethane to give **11** in good yield.^10^ The hydrogenative reduction of the nitro group in the presence of trifluoroacetic acid (TFA) followed by treatment of the crude amine **12** with pyrrole trichloromethyl ketone **13** afforded the desired pyrrole amide **14**.^10^ The acylation of the secondary alcohol of **14** induced the E1cB elimination to give the desired enone **8** as a single stereoisomer. Next, treatment of **8** with hydrazine at 100 °C produced the pyrazolidine ring by 1,4-addition and hydrazone-forming reactions,^12^ and because the resulting ring was not sufficiently stable for isolation and purification, benzyloxycarbonylthioisocyanate (CbzNCS) was directly added to introduce the thiourea, yielding **15** as an inseparable diastereomeric mixture.^3*e*,10^ Treatment of the crude **15** with an excess amount of TFA and NaCN initiated the Strecker reaction of the desired diastereomer that possesses an α-aminomethyl side chain to give nitrile **16**,^13^ while the undesired diastereomer did not give the corresponding nitrile due to the steric repulsion of the β-side chain on the concave face. Development of the Strecker reaction on the pyrazoline ring enabled efficient construction of the tetra-substituted carbon center possessing nitrogen, which previously required many steps in the first-generation synthesis. Next, thiourea **16** was converted into methylisothiourea **17**, and the trifluoroacetyl group as a strong electron-withdrawing group was introduced to the nitrogen on the tetra-substituted carbon center.^10^ Because an excess amount of trifluoroacetic anhydride needed to be introduced to the desired nitrogen, the pyrrole nitrogen was also acetylated to give **18**. Finally, the trifluoroacetyl group on the pyrrole nitrogen was selectively removed by transfer to benzylamine to afford the desired cascade cyclization precursor **6** in acceptable yield (Scheme 1).

### The first application of **6** to the cascade cyclization reaction

**Scheme 2:**
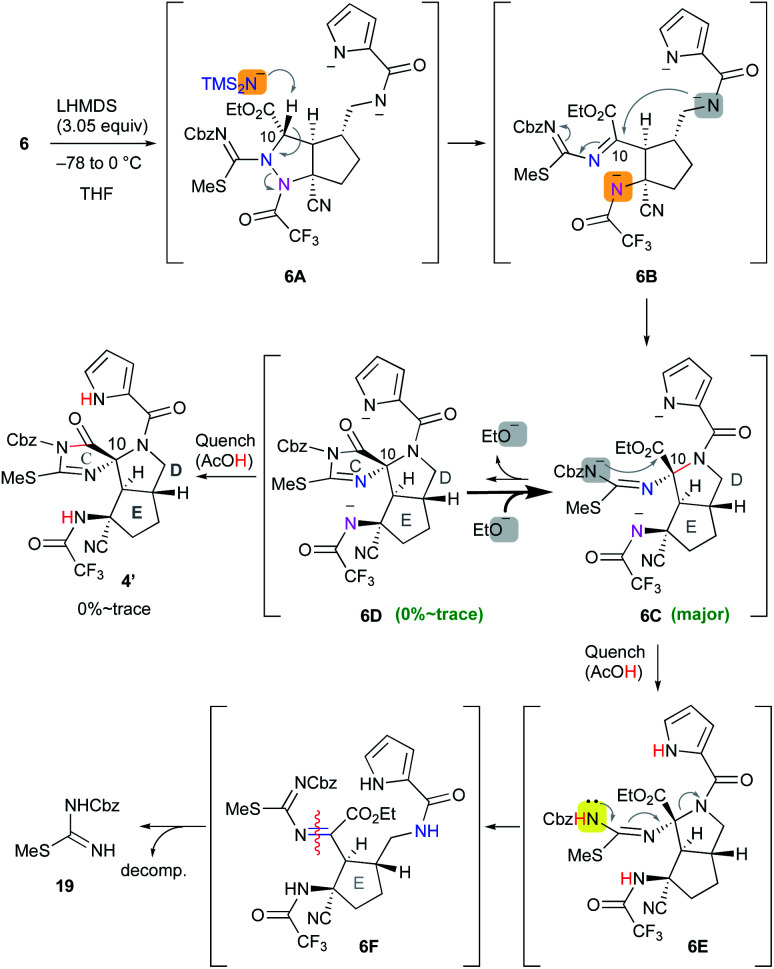
Application of **6** to the cascade cyclization reaction.

Having prepared the cascade cyclization precursor **6**, we next attempted single-step construction of the CDE tricyclic ring system. First, treatment of **6** with 3.05 equiv. of lithium hexamethyldisilazide (LHMDS) under conditions similar to those used in the first-generation synthesis^10^ afforded a trace amount of the tricyclic compound **4′**, which was later found to be a diastereomer at the C10 position. However, the major isolable product was isothiourea **19**, and several trials of this reaction always yielded **19** as the major product and often resulted in less than even a trace amount of **4′**. The mechanism for the formation of isothiourea **19** was considered to be the following: the cascade reaction of N–N bond cleavage of **6A** followed by addition of the amide anion to the imine moiety of **6B** proceeded smoothly to give **6C**. However, it was considered that the further cyclization reaction of **6C** to give **6D** hardly proceeded, because the highly reactive ethoxide generated by this conversion readily attacked the unstable cyclic imide moiety of **6D** to return to the stable carbamate anion of **6C**. This means that the equilibrium between **6C** and **6D** greatly favored the former. Quenching the reaction by protonation of the equilibrium mixture afforded **6E** and a trace amount of **4′**, and the former immediately opened the highly strained D ring by the electron-donating effect of the isothiourea moiety to give **6F**. Subsequent hydrolysis of **6F** afforded isothiourea **19** along with the decomposition of other fragments (Scheme 2). On the other hand, tricyclic **4′** could be isolated, suggesting that the progress of the conversion of **6C** into **6D** is essential to obtain the *trans*-azabicylo[3.3.0]octane skeleton (D/E ring junction). Thus, we focused on proceeding with this disadvantageous conversion, considering that it might later be possible to invert the stereocenter at the C10 position.^5*t*^

### Application of selective protonation of the most reactive anion

**Scheme 3:**
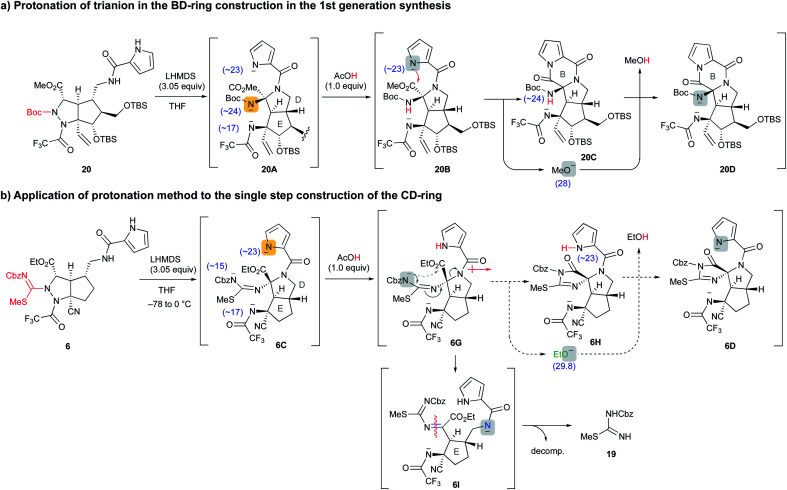
Application of the selective protonation method to the cascade reaction of **6**.

A similar situation was observed in the first-generation synthesis,^10^ and we solved this problem as follows (Scheme 3a). The cascade reaction of **20** proceeded smoothly to construct the D ring through a similar N–N bond cleavage followed by the addition of an amide anion. After checking the conversion of **20** to **20A** by TLC, an exact 1.0 equiv. of acetic acid was added to the reaction mixture. The intermediate **20A** possesses three nitrogen anions, and the most reactive Boc-carbamate anion was protonated (**20A** → **20B**). Then, addition of the remaining pyrrole anion to the methyl ester formed a B ring along with the generation of the methoxide (**20B** → **20C**). In this case, the methoxide did not attack the pyrrole amide of the B ring due to the active NH proton of Boc-carbamate, *i.e.*, the methoxide preferentially abstracted the NH proton and was quenched (**20C** → **20D**). In the case of the cascade reaction of **6** (Scheme 3b), the addition of 1.0 equiv. of acetic acid after the formation of **6C** would have protonated the pyrrole anion to give **6G**, because the basicity of the three nitrogen anions of **6C** decreased in the order pyrrole (<23), trifluoroacetamide (∼17), and acylisothiourea (∼15) based on a comparison of the p*K*_a_ values of the protonated NH functional groups.^14^ Then, subsequent addition of the remaining acylisothiourea anion to the ethyl ester was expected to give **6H**, simultaneously generating the ethoxide, which would not attack the cyclic imide moiety but rather would abstract the NH proton of pyrrole.

However, the protonation of the pyrrole anion induced an increase in the inductive effect of the pyrrole amide, so the acylisothiourea anion actually extruded the amide nitrogen of the D ring faster than the addition to the ethyl ester, opening the D ring to give **6I**. The resulting **6I** was readily decomposed, giving isothiourea **19** as with the previous reaction that did not add acetic acid. Therefore, it was found that the previous results of the first-generation synthesis were not applicable to the cascade reaction of **6** in this study. As far as our study is concerned, the formation of cyclic imide from the less nucleophilic isothiourea was difficult, and we had to find another strategy to proceed with the unfavorable equilibrium reaction from **6C** to **6D**. In addition, in the case of the active esters, such as hexafluoroisopropyl ester, which generates a less reactive alkoxide instead of the ethoxide, the D-ring formation did not proceed because the reaction of the amide anion with the active esters took precedence. Further investigation of various precursors with other functional groups did not afford the desired CDE tricyclic compound (see Fig. S1 in the ESI†).

### Discovery of Ph_2_NLi as the base for proceeding with the reaction from **6C** to **4′**

**Scheme 4:**
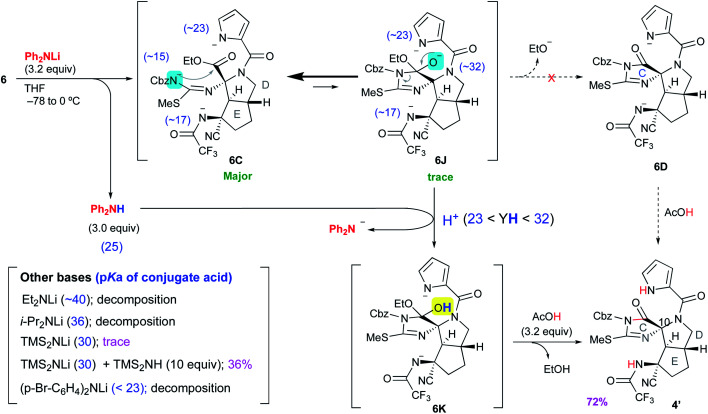
Successful conversion of **6** to **4′**.

We carefully reconsidered the reaction mechanism and focused on the alkoxide intermediate **6J** (Scheme 4). Although the alkoxide intermediates are often omitted when considering the addition reaction to carbonyl groups, we utilized this intermediate to procced with this disadvantageous cascade reaction as follows. The use of 3.2 equiv. of lithium diphenylamide (Ph_2_NLi) led to a similar D-ring forming reaction to give **6C**, while simultaneously generating 3.0 equiv. of diphenylamine (Ph_2_NH). The equilibrium between **6C** and **6J** greatly favored the former because the anion of acylisothiourea **6C** was much more stable than the alkoxide of **6J**, but the small amount of **6J** generated was protonated by the coexisting Ph_2_NH. This selective protonation of the alkoxide removed **6J** from equilibrium to give **6K**, and the equilibrium mixture of **6C** and **6J** finally converged into **6K**. Quenching the reaction with 3.2 equiv. of acetic acid induced the elimination of ethanol to give **4′** in 72% yield. Although Ph_2_NLi has rarely been used as a base, we came up with the use of this base as follows. The key to the cascade reaction forming the CDE ring core in a single step was the selective protonation of the generated alkoxide of **6J**. Because the protonation of other anions, such as the pyrrole anion, induces rapid decomposition as described in Scheme 3, the protonation of only the alkoxide of **6J** was essential despite the extremely low abundance of **6J** in the equilibrium of **6C** and **6J**. Thus, we investigated the coexistence of appropriate acids that can protonate only a trace amount of the alkoxide of **6J** and do not protonate other major anions. Comparing the p*K*_a_ of conjugate acids of the six anions in the equilibrium between **6C** and **6J**, the most basic anion is the alkoxide of **6J** (∼32) and the second basic anion is the pyrrole anion (∼23). Therefore, the appropriate p*K*_a_ for a coexisting acid (YH) that can protonate the alkoxide but not the pyrrole anions was expected to be 23–32, and the diphenyl amine with a p*K*_a_ of 25 in organic solvent was considered suitable as such an acid. Based on the above consideration, Ph_2_NLi was adopted as the base that can supply Ph_2_NH *in situ* after the abstraction of protons. As we expected, the use of Ph_2_NLi afforded the CDE-tricyclic ring core in good yield, and we established an efficient and interesting reaction system in which the employed base functions not only as a base but also as an acid (Scheme 4).

Next, to confirm our p*K*_a_ concept for proceeding with the unfavorable equilibrium reaction, similar reactions using other bases that generate conjugate acids with p*K*_a_ beyond the range of 23–32 were examined. The use of lithium diethylamide (Et_2_NLi) or lithium diisopropylamide (LDA) as the base resulted in a complex mixture including isothiourea **19** as the major product, and the desired **4′** was not detected. The p*K*_a_ values of the conjugate acids of Et_2_NLi and LDA are 40 and 36, respectively, which are much higher than that of the alkoxide of **6J** (∼32), and the conjugate acids generated *in situ*, such as Et_2_NH and ^*i*^Pr_2_NH, were not able to protonate the alkoxide of **6J**. Thus, the reaction stopped at **6C**, which was decomposed after workup. Next, in the case of 3.0 equiv. of LHMDS, a trace amount of **4′** was obtained as described in Scheme 2. Since the p*K*_a_ value of 30 for conjugate acid (Me_3_Si)_2_NH is close to that of the conjugate acid of the alkoxide of **6J** (∼32), (Me_3_Si)_2_NH could protonate the alkoxide to give **6K**, but the regenerated anion LHMDS also abstracted the hydrogen of the hydroxy group of **6K**. Thus, the equilibrium between **6C** and **6J** was extended to **6K**, but the stable anion **6C** was greatly favored in this equilibrium so **6K** was present in very small amounts, which resulted in a trace amount of **4′** after workup. To increase the abundance ratio of **6K** in equilibrium, a similar reaction was conducted in the presence of an excess amount of (Me_3_Si)_2_NH as the co-existing acid, and the yield of **4′** was improved to 36%. On the other hand, when lithium di-*p*-bromophenylamide ((*p*-Br-C_6_H_4_)_2_NLi), which possesses an electron withdrawing bromo group on the phenyl ring, was adopted as the base having a p*K*_a_ of the conjugate acid of less than 23, the reaction afforded only decomposition because the pyrrole anion of **6C**, which is highly abundant in equilibrium, could be protonated by the generated conjugate acid (*p*-Br-C_6_H_4_)_2_NH, and the protonated **6C** was readily decomposed to **6G** as shown in Scheme 3. Therefore, it was confirmed that the p*K*_a_ of the conjugate acid was the significant factor for proceeding with this disadvantageous reaction.

### Concept of p*K*_a_ for proceeding with the unfavorable equilibrium reaction

**Fig. 3:**
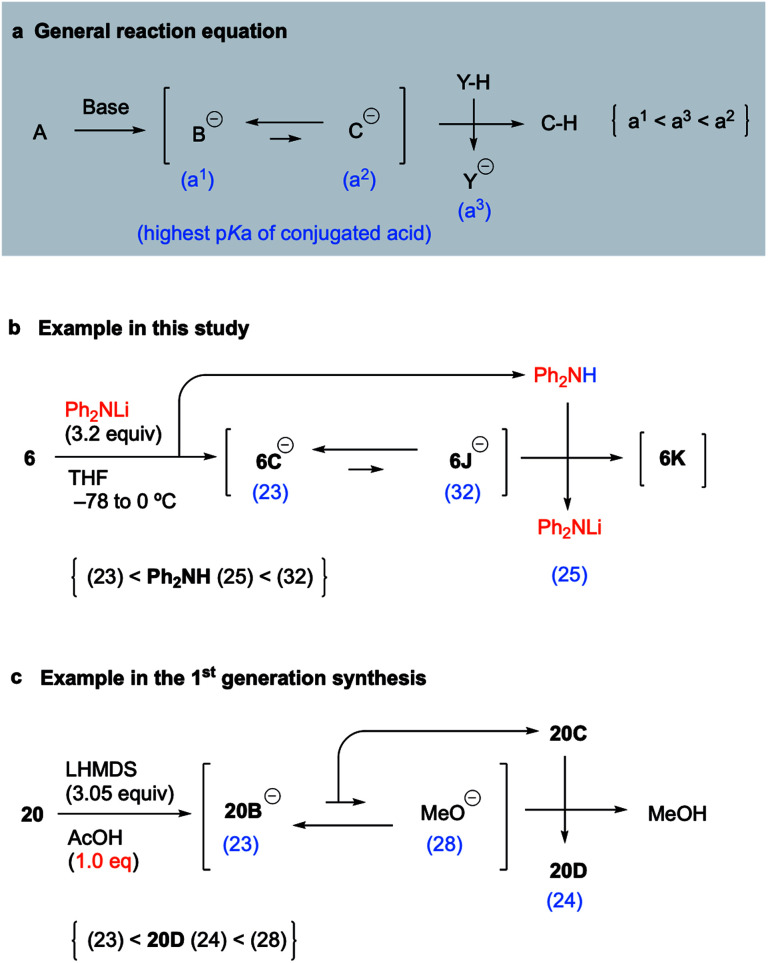
General equation for proceeding with an unfavorable equilibrium reaction.

Because the above concept of p*K*_a_ was considered to be applicable to various unfavorable equilibrium reactions, we proposed the following equation as a general concept for proceeding with such reactions (Fig. 3a). When treatment of **A** with a base provides the equilibrium between anions **B** and **C** in which the p*K*_a_ of the conjugate acid of **B** is much lower than that of **C**, the equilibrium of the two anions is much more favorable to **B** as a stable anion. If anion **C** was the intermediate that leads to the desired product **C–H** by protonation, a simple workup will afford **B–H** as the major product instead of **C–H**. To obtain **C–H** as the major product, only the anion of **C** should be quenched by the coexistence of a suitable acid that has a lower p*K*_a_ than the conjugate acid of **C** and a higher p*K*_a_ than the conjugate acid of **B**. Thus, assuming that the p*K*_a_ values of the conjugate acids of **B** and **C**, and that of the coexisting acid (**Y–H**), are a^1^, a^2^, and a^3^, respectively, the adequate p*K*_a_ of the coexisting acid (**Y–H**) holds the relationship a^1^ < a^3^ < a^2^ (Fig. 3a). Of course, this p*K*_a_ concept is observed as a matter of course by synthetic chemists, but it is generally mastered based on the knowledge and experience of the individual synthetic chemist and has not been made available as a general equation. Thus, we suggested the general equation based on the results of key reactions in the 1st generation synthesis and this synthetic study of palau'amine. In this synthetic study, the p*K*_a_ of Ph_2_NH generated *in situ* as the coexisting acid is 25, and the p*K*_a_ order is **6C** (23) < Ph_2_NH (25) < **6J** (32), which satisfies the general equation (Fig. 3b). In addition, in the first-generation synthesis, although the equilibrium between the pyrrole anion of **20B** and methoxide favored the former, the carbamate moiety (NHBoc) of cyclized **20C** can be regarded as a coexisting acid that can protonate the methoxide. The p*K*_a_ of Boc-carbamate (NHBoc) is estimated to be around 24, and the p*K*_a_ order is the pyrrole anion of **20D** (23) < NHBoc (24) < methoxide (28), which also satisfies the general equation (Fig. 3c). We are currently investigating the scope of the general equation.

### Inversion of the stereocenter at the C10 position

**Scheme 5:**
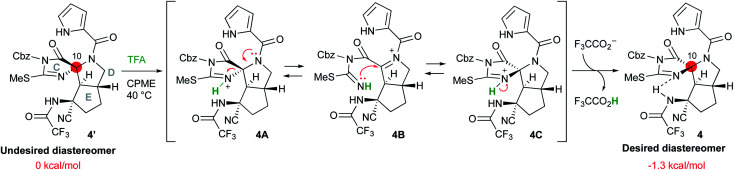
Inversion of the stereocenter at the C10 position.

Having synthesized the CDE tricyclic compound **4′** in acceptable yield, we attempted the stereo inversion of the C10 position (Scheme 5). DFT studies have suggested that the desired configuration at the C10 position is thermodynamically 1.3 kcal mol^−1^ more stable than that of **4′** due to the hydrogen bonding of the NH proton of trifluoroamide to the nitrogen of the C-ring. Thus, epimerization under acidic conditions was investigated,^5*t*^ and it was found that treatment of **4′** with 100 equiv. of TFA in cyclopentylmethylether (CPME) afforded the desired **4** in good yield. The inversion reaction was considered to proceed as follows: the most basic nitrogen of the C-ring was initially protonated to give **4A**, and the C-ring was subsequently opened by the electron donation from the amide nitrogen of the D-ring so that the acyliminium intermediate **4B** was formed. Ring closure of the C-ring by the addition of isothiourea to the acyliminium moiety afforded **4C**, which was converted into the desired **4** by deprotonation. These reversible sequential reactions finally converged into thermodynamically stable **4**.

### Reduction of the C-ring carbonyl group

**Scheme 6:**
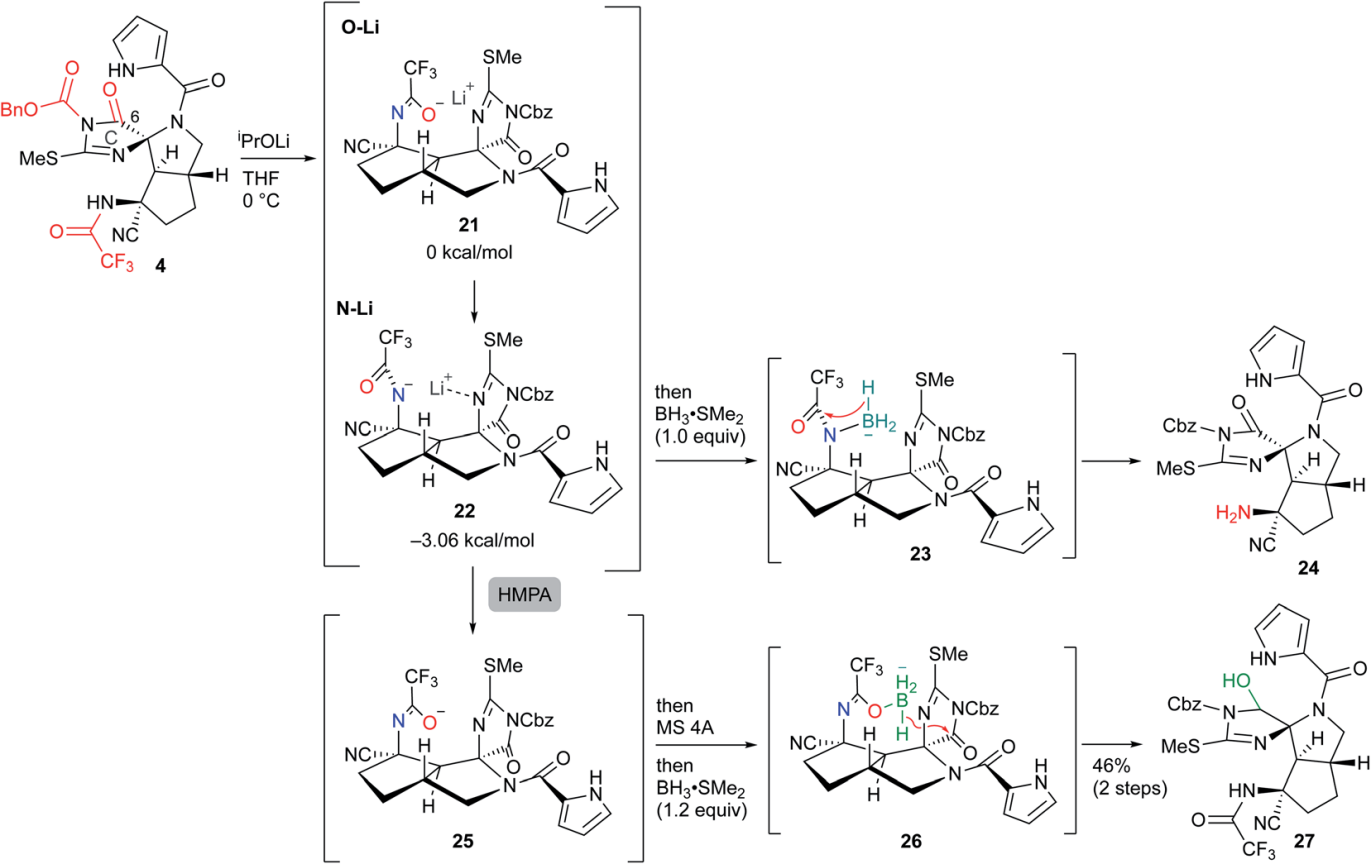
Selective reduction of the least reactive carbonyl group at the C6 position.

Next, selective reduction of the C-ring carbonyl group was required to form the B-ring. However, despite various examinations of the reducing agents and conditions, the reductive elimination of the Cbz group or trifluoroacetyl group proceeded in preference to the reduction of the imide carbonyl group of the C-ring, suggesting that it was the least reactive carbonyl group among the three carbonyl groups of **4** (see Scheme S1 in the ESI†). Moreover, even after various conversions of **4**, the selective reduction was still difficult (see Scheme S2 in the ESI†). Thus, to overcome the low reactivity, we planned to utilize the neighboring effect of the trifluoroacetyl group as follows: after the generation of the amide anion by basic treatment, subsequent addition of BH_3_ would form the borohydride with the oxygen of the aza enolate, which would selectively reduce the adjacent carbonyl group of the C-ring. However, sequential treatment of ^*i*^PrOLi and BH_3_·SMe_2_ mainly induced the reductive elimination of the trifluoroacetyl group to give **24**, and the yield of the desired hemiaminal **27** was very low. The main reason for this result was considered to be that the anion of aza enolate was located on the nitrogen and therefore formed the borohydride **23**, reducing the trifluoroacetyl group. Indeed, DFT studies demonstrated that the lithium–nitrogen bond shown in **22** was 3.06 kcal mol^−1^ more stable than the lithium–oxygen bond shown in **21** due to the stabilization of the lithium cation by the adjacent nitrogen of the C-ring. Thus, after the treatment of ^*i*^PrOLi, hexamethylphosphoric triamide (HMPA) was added to form oxygen anion **25**, and direct addition of molecular sieves 4A (MS 4A) followed by BH_3_·SMe_2_ resulted in the formation of **26**, which was converted into the desired hemiaminal **27** (Scheme 6). MS 4A was required to remove the trace amount of water in THF that gradually decomposed the resulting **27** in the reaction mixture.

### Synthesis of palau'amine analog **2**

**Scheme 7:**
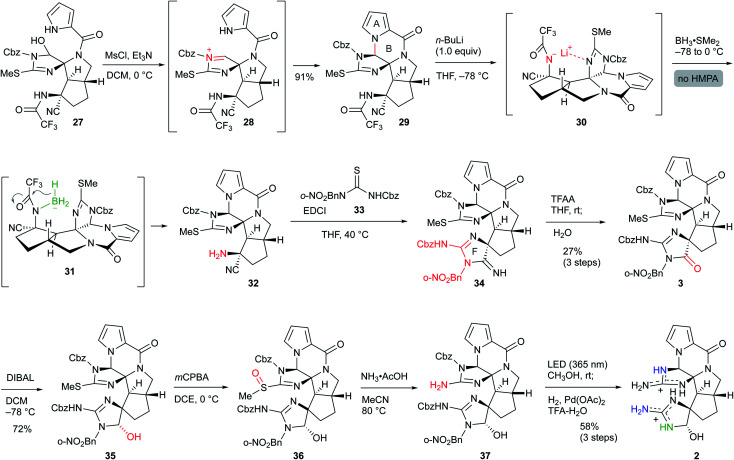
Synthesis of palau'amine analog **2**.

Having synthesized the CDE tricyclic core **27**, we finally converted **27** to palau'amine analog **2** (Scheme 7). The mesylation of **27** induced the elimination of the hydroxy group to give the reactive acyliminium cation **28**, which subsequently formed the B-ring by the addition of the pyrrole nitrogen, and ABCDE pentacyclic core **29** was obtained in 91% yield. Next, removal of the trifluoroacetyl group was required for the construction of the F-ring. This time, the findings in Scheme 6 were applied to the selective reductive elimination of the trifluoroacetyl group. The nitrogen anion was initially generated by the addition of ^*n*^BuLi, and subsequent treatment of BH_3_·SMe_2_ without HMPA formed borohydride **30**, which induced the reduction of the trifluoroacetyl group to give **32**. Because the aminonitrile **32** was not sufficiently stable for the purification step, the crude product was treated directly with isothiourea **33** to give **34** with the F-ring through the guanidine formation followed by the intramolecular addition to the nitrile.^15^ The crude **34** was also directly used for the next reaction due to its instability and high polarity. The amidine moiety of **34** was trifluoroacetylated for activation, and subsequent hydrolysis afforded amide **3** in 27% three-step yield from **29**. The other methods and other analogs were unable to construct the F-ring (see Scheme S3 in the ESI†). DIBAL reduction of the amide moiety of **3** gave the aminal **35** in 72% yield.^16^ Finally, the isothiourea group of the C-ring was oxidized to sulfoxide **36**, and subsequent treatment with ammonium acetate afforded guanidine **37**.^17^ The crude **37** was directly deprotected by the sequential reactions of photoirradiation and hydrogenation for removal of the *o*-nitrobenzyl group and Cbz group, respectively,^10^ and the palau'amine analog **2** was obtained without significant byproducts. The HPLC purification using a Hydrosphere C18 gave pure **2** in 58% isolated yield from **35**.^18^ The structure of **2** was confirmed by NMR, HRMS, and comparison with palau'amine **1**.

### Biological test of palau'amine analog **2**

**Fig. 4:**
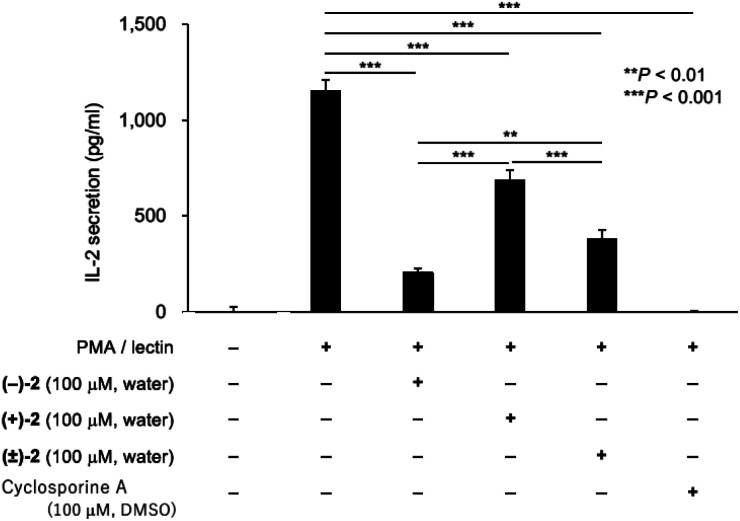
Immunosuppressive activity of palau'amine analog **2**. Splenic lymphocytes from BALB/c mouse (male, 5 weeks old, Japan SLC, Shizuoka, Japan) were treated with an aqueous solution of (−)-**2** (100 μM), (+)-**2** (100 μM), (±)-**2** (100 μM), and cyclosporine A (100 μM) at 37 °C for 1 h, and then the cells were incubated with phorbol 12-myristate 13-acetate (PMA, 10 nM) and lectin (1.0 μg ml^−1^). After incubation for 24 h, the IL-2 in the culture supernatant was measured by using an ELISA kit. Values are means with standard deviation indicated by error bars (*n* = 3, **P < 0.01, ***P < 0.001, *F* = 345.9, 95% CI, df = 10). Statistical differences between the groups were evaluated by one-way analysis of variance (ANOVA) with the Tukey *post-hoc* test using Prism 8 software (GraphPad Software, San Diego, CA, USA). The immunosuppressive activities were measured 2 times using independent splenic lymphocytes.

The immunosuppressive activity of palau'amine analog **2** was examined (Fig. 4). To evaluate the effect of enantiomer on activity, enantiomers of synthetic intermediate **3** were separated by the chiral HPLC, and each enantiomer was similarly converted to (+)-**2** and (−)-**2**, respectively. We still have a small amount of previously synthesized palau'amine **1** in stock, but since synthetic **1** is highly valuable (45-step synthesis), we have left it for future mechanistic studies. We previously found that synthetic **1** and cyclosporine A (CSA) showed equivalent immunosuppressive activity at 100 μM.^10^ Therefore, CSA was used as an activity index instead of valuable synthetic palau'amine **1** in this experiment. Lymphocytes derived from a mouse spleen were treated with 100 μM of an aqueous solution of synthetic (−)-**2**, (+)-**2**, or (±)-**2** for 1 h and then the cells were incubated with phorbol 12-myristate 13-acetate and lectin.^10,19^ After incubation for 24 h, the interleukin-2 (IL-2) in the culture supernatant was measured by using an enzyme-linked immunosorbent assay kit. The palau'amine analog (±)-**2**·2TFA salt was confirmed to retain immunosuppressive activity, as shown in Fig. 4. Thus, it was found that the chloride and the aminomethyl groups are not crucial for immunosuppressive activity. However, the immunosuppressive activity of (±)-**2** was lower than that of synthetic racemic palau'amine, which showed activity similar to that of cyclosporine A,^10^ and these substituents were not completely independent of activity. Not surprisingly, one enantiomer, (−)-**2**, was more active than (±)-**2**, but the opposite enantiomer (+)-**2** also interestingly showed immunosuppressive activity. The activity of racemic (±)-**2** was just between those of the two enantiomers. Detailed mechanistic analysis of these immunosuppressive activities is the next research subject.

## Conclusions

We have achieved a reduction in the number of steps required for the construction of the ABCDEF hexacyclic ring core of palau'amine as a second-generation synthesis. To shorten the number of steps for the formation of the C-ring, the isothiourea group was previously introduced to the cascade cyclization precursor. The cascade reaction of this precursor had to proceed through a very unfavorable intermediate in the equilibrium mixture. Thus, we established the p*K*_a_ concept to overcome the disadvantageous reaction, which selectively protonated the unfavorable intermediate to remove it from equilibrium so that the equilibrium mixture converged into its protonated form. Since we considered that the p*K*_a_ concept in this cascade reaction is applicable to various disadvantageous reactions, a general equation was suggested. Although this p*K*_a_ concept is observed as a matter of course by synthetic chemists, it is generally mastered based on the knowledge and experience of the individual chemist and has not been made available as a general equation. The scope and limitation of this p*K*_a_ concept are currently being investigated in our laboratory. The p*K*_a_ concept established in this study clearly enhanced the short-step construction of the ABCDEF tetracyclic ring core, and the palau'amine analog **2** was actually obtained in only 20 steps from cyclopentenone **9** (longest linear sequence). Compared with the 45 steps required for the first-generation synthesis of palau'amine **1**, this constitutes a substantial reduction in steps. Indeed, this short-step synthesis allowed us to obtain over 100 mg of **3**. However, large-scale synthesis of **2** has not been easy so far due to the instability of **2** under HPLC purification conditions. Only the Hydrosphere C18 among various columns was applicable to **2**, but it also still induced partial decomposition. The development of the purification method such as recrystallization is the next issue to achieve large scale synthesis. Although the chloride and the aminomethyl groups were omitted, the immunosuppressive activity was retained. Thus, another point to be mentioned is that the aminomethyl group of palau'amine can serve as a potential position for the labeling of palau'amine analogs for functional studies, and the removal of the chloride group improved the stability of various probes. Another analog with an aminomethyl group could be synthesized based on this study without significantly increasing the number of steps. For example, a protected aminomethyl or hydroxymethyl group would be pre-introduced at the 4-position of cyclopentenone, and its conversion in a sequence similar to that used in this study would provide the desired analog for a palau'amine probe. An application of this study to asymmetric synthesis, synthesis of related pyrrole–imidazole alkaloids, and development of palau'amine probes is currently underway in our laboratory.

## Methods

General information, experimental details of the synthesis and biological test, spectral data, and ^1^H and ^13^C NMR charts are included in the ESI.† S.E.E., who performed the biological test in Fig. 4, has been educated in Animal Care and Animal Ethics in Tokushima University. Experiments using mice in Fig. 4 were approved by the Animal Care and Animal Ethics Committees of Tokushima University (No. T2019-47).

## Data availability

Experimental or computational data associated with this article have been provided in the ESI.†

## Author contributions

K. N. conceived the experiments and analyzed the results. E. O. performed the laboratory experiments and optimized the reaction conditions, and K. Takeuchi and A. N. helped with experiments. S. E. E., H. A. and T. I. performed a biological test of immunosuppressive activity. S. K. performed the DFT analysis of key intermediates. K. N. wrote the paper.

## Conflicts of interest

There are no conflicts to declare.

## Supplementary Material

SC-012-D1SC03260G-s001

## References

[cit1] Forte B., Malgesini B., Piutti C., Quartieri F., Scolaro A., Paeo G. (2009). Mar. Drugs.

[cit2] Kinnel R. B., Gehrken H.-P., Scheuer P. J. (1993). J. Am. Chem. Soc..

[cit3] Grube A., Köck M. (2007). Angew. Chem., Int. Ed..

[cit4] Lansdell T. A., Hewlett N. M., Skoumbourdis A. P., Fodor M. D., Seiple I. B., Su S., Baran P. S., Feldman K. S., Tepe J. J. (2012). J. Nat. Prod..

[cit5] Overman L. E., Rogers B. N., Tellew J. E., Trenkle W. C. (1997). J. Am. Chem. Soc..

[cit6] Starr J. T., Koch G., Carreira E. M. (2000). J. Am. Chem. Soc..

[cit7] Gaich T., Baran P. S. (2010). Org. Chem..

[cit8] Seiple I. B., Su S., Young I. S., Lewis C. A., Yamaguchi J., Baran P. S. (2010). Angew. Chem., Int. Ed..

[cit9] Seiple I. B., Su S., Young I. S., Nakamura A., Yamaguchi J., Jørgensen L., Rodriguez R. A., O'Malley D. P., Gaich T., Köck M., Baran P. S. (2011). J. Am. Chem. Soc..

[cit10] Namba K., Takeuchi K., Kaihara Y., Oda M., Nakayama A., Nakayama A., Yoshid M., Tanino K. (2015). Nat. Commun..

[cit11] Namba K., Kaihara Y., Yamamoto H., Imagawa H., Tanino K., Williams R. N., Nishizawa M. (2009). Chem. – Eur. J..

[cit12] Zohry M. F. E., Awad I. M. A., Hafez A. A. A. (1993). Arch. Phrma..

[cit13] Namba K., Shinada T., Teramoto T., Ohfune Y. (2000). J. Am. Chem. Soc..

[cit14] It is assumed that the p*K*_a_ value in an organic solvent is close to literature values measured in not water but DMSO. To the best of our knowledge, literature values in organic solvents other than DMSO were rare. For p*K*_a_ values, see p*K*_a_ values compilation https://organicchemistrydata.org/hansreich/resources/pka/pka_data/evans_pKa_table.pdf (by D. Evans and D. H., Ripin) and p*K*_a_ values in DMSO compilation (by Reich and Bordwell) https://organicchemistrydata.org/hansreich/resources/pka/#pka_dmso_compilation, and references are therein. The p*K*_a_ values in the figures were estimated based on the above table and the order of p*K*_a_ values within the compound was confirmed by DFT calculation [B3LYP/6-31+G(d,p)]

[cit15] Linton B. R., Carr A. J., Orner B. P., Hamilton A. D. (2000). J. Org. Chem..

[cit16] Tang L., Romo D. (2007). Heterocycles.

[cit17] Takeuchi K., Nakayama A., Tanino K., Namba K. (2016). Synlett.

[cit18] Araki A., Kubota T., Tsuda M., Mikami Y., Fromont J., Kobayashi J. (2008). Org. Lett..

[cit19] Granelli-Pipemoand A., Nolan P. (1991). J. Immunol..

