# Supplementary material for: Efficient construction of the hexacyclic ring core of palau'amine: the pKa concept for proceeding with unfavorable equilibrium reactions
Source: Chem Sci. 2021 Aug 11;12(36):12201–10. doi: 10.1039/d1sc03260g (PMC8457368; doi:10.1039/d1sc03260g)
Supplement: SC-012-D1SC03260G-s001 [file SC-012-D1SC03260G-s001.pdf]

Electronic Supplementary Material (ESI) for Chemical Science.
This journal is © The Royal Society of Chemistry 2021

Supporting Information

Efficient Construction of Hexacyclic Ring Core of Palau'amine: Concept of pKa for Proceeding with Unfavorable Equilibrium Reactions

Eisaku Ohashi,[a] Sangita Karanjit,[a,b] Atsushi Nakayama,[a,b] Kohei Takeuchi,[a] Sherif E. Emam,[a] Hidenori Ando,[a] Tatsuhiro Ishida,[a] Kosuke Namba*[a,b]

[a]Graduate School of Pharmaceutical Sciences, Tokushima University. 1-78 Shomachi, Tokushima 770-8505 (Japan)

[b]Research Cluster on "Innovative Chemical Sensing", Tokushima University. 1-78 Shomachi, Tokushima 770-8505 (Japan)

E-mail: namba@tokushima-u.ac.jp

General Procedures and Methods

The reactions were performed using flame-dried glassware under a positive pressure of argon. Et_3N and $^iPr_2NEt$ were distilled from CaH_2 under argon atmosphere and stored over KOH. HMPA was distilled from CaH_2 under argon atmosphere and stored over MS 4A. Solution and solvent were introduced by hypodermic syringe through a rubber septum. During the reaction, the vessel was kept under a positive pressure of argon. Anhydrous $CHCl_3$, DCM, EtOH, MeOH, THF were purchased from Kanto Chemical Co. Inc. Anhydrous DCE, ^iPrOH, MeCN and $MeNO_2$ were purchased from FUJIFILM Wako Pure Chemical Corporation. Anhydrous CPME was purchased from Tokyo Chemical Industry Co. Ltd. All other reagents were used as received from commercial sources without further purification.

Infrared (IR) spectra were recorded on JASCO FT/IR-4100 spectrophotometer using 5 mm KBr plate. Wavelengths of maximum absorbance are quoted in cm^{-1}. ^{1}H-NMR spectra were recorded on a Bruker AV–400 (400 MHz), and Bruker AV–500 (500 MHz) in $CDCl_3$, CD_3CN and CD_3OD. Chemical shifts are reported in part per million (ppm), and signal are expressed as singlet (s), doublet (d), triplet (t), quartet (q), multiplet (m) and broad (br). ^{13}C-NMR spectra were recorded on a Bruker AV–400 (100 MHz), and Bruker AV–500 (125 MHz) in $CDCl_3$, CD_3CN and CD_3OD. Chemical shifts are reported in part per million (ppm). High resolution mass (HRMS) spectra were

recorded on a Thermo Scientific Exactive, High performance liquid chromatography (HPLC) was recorded on ChromNAV (JASCO Corporation). Analytical thin layer chromatography (TLC) was performed using 0.25 mm E. Merck Silica gel (60F-254) plates. Reaction components were visualized phosphomolybdic acid or ninhydrin or *p*-anisaldehyde in 10% sulfuric acid in ethanol. Kanto Chem. Co. Silica Gel 60N (particle size 0.040–0.050 mm) and Merch Aluminium oxide 90 active basic (0.063-0.200 mm) was used for column chromatography. The photoirradiation was carried out by using PER-AMP (LED-UV, 365 nm, Techno Sigma Co., Ltd.).

• Figure S1 S3

• Scheme S1-S3 S3-S5

• Experimental Detail (Synthesis) S6-S19

• Experimental Detail (Biological test) S20

• Computational Detail S21-S29

• ^{1}H NMR and ^{13}C NHR S30-S59

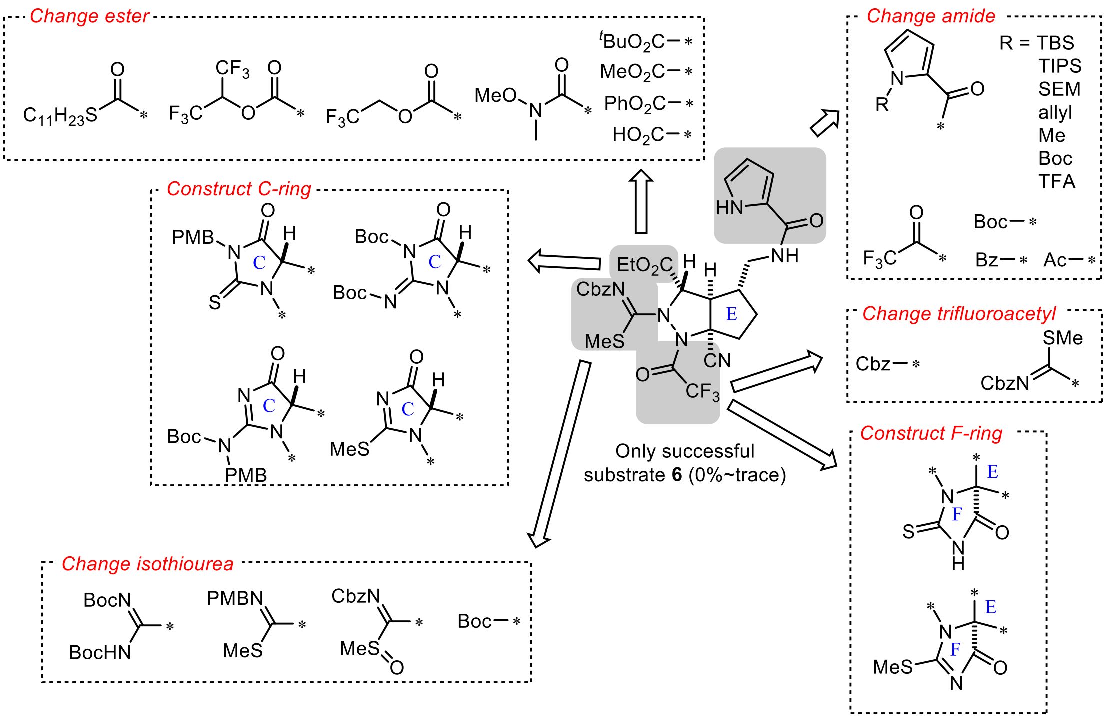

Figure S1. Other precursors unsuitable for the cascade cyclization reaction. Various precursors other than **6**, in which ester, isothiourea, trifluoroacetyl group or pyrrole amide was changed, were also examined for the cascade cyclization reaction. All analogs did not afford desired compounds as the major product.

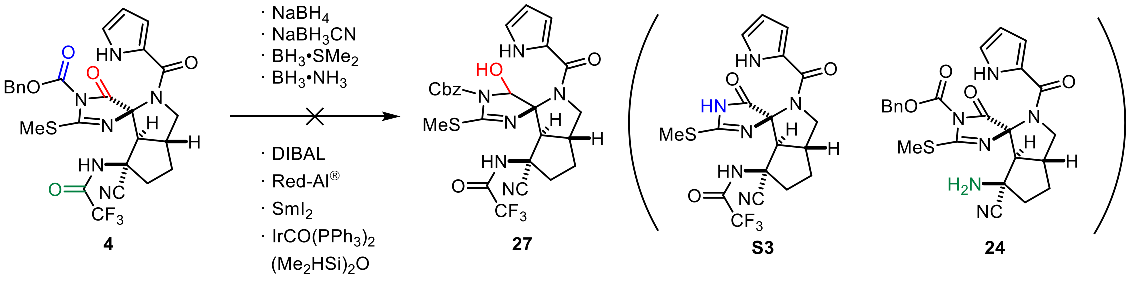

Scheme S1. Investigation of reductants for the selective reduction of the carbonyl group on the C-ring. Various reductants were investigated for the selective reduction of the carbonyl group on the C-ring. However, the reductive elimination of the Cbz group or trifluoroacetyl group proceeded in preference to the reduction of the carbonyl group of the C-ring.

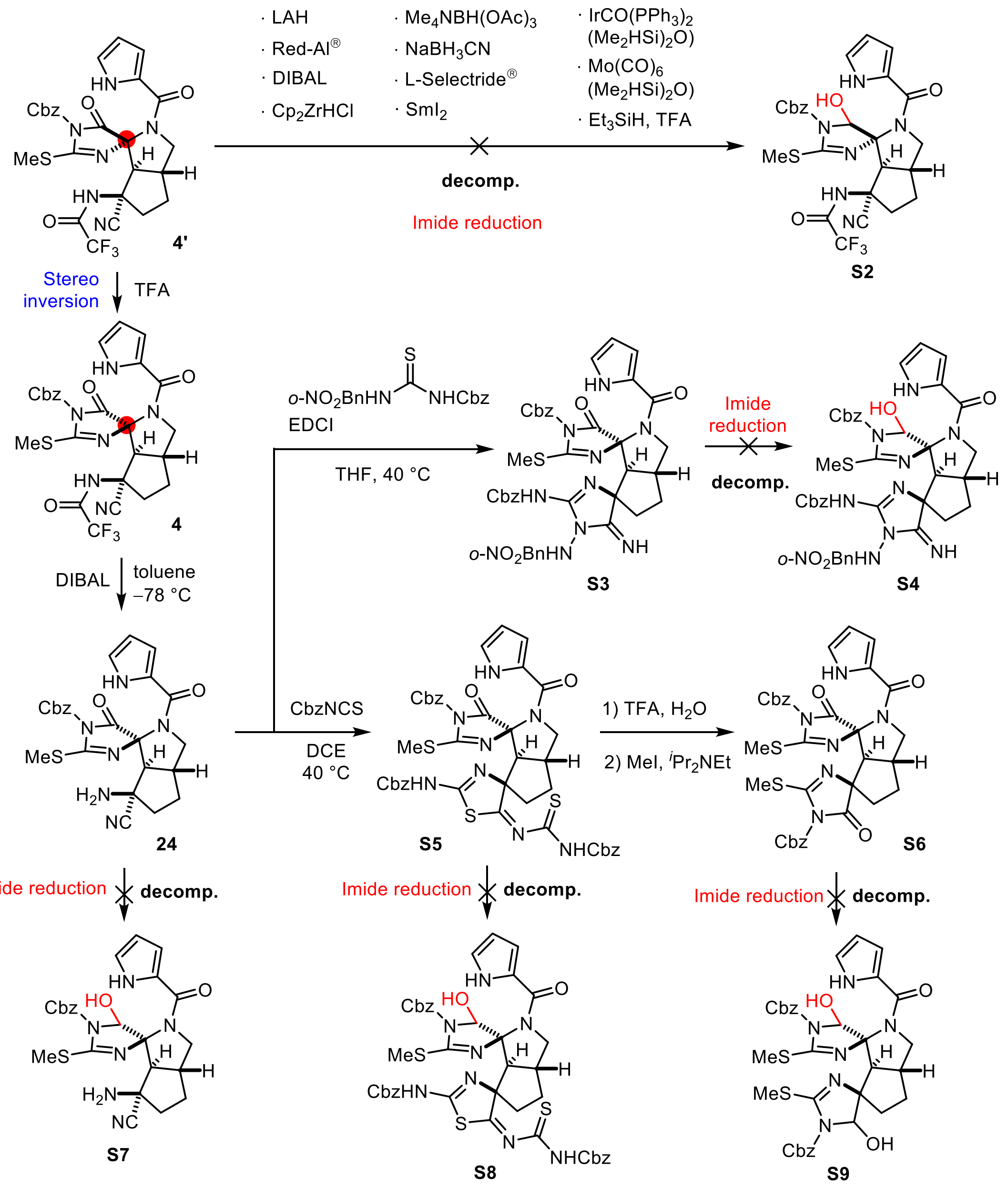

Scheme S2. Substrate investigation for reduction of the carbonyl group on the C-ring. Various compounds **4'**, **24**, **S3**, **S5**, **S6** were also examined for the reduction of the carbonyl group on the C-ring. All substrates lead to decomposition. Compounds **24**, **S5**, **S6** were synthesized from **4'** by stereo inversion, a removal of the trifluoroacetyl group, installation of isothiourea, hydrolysis, and methylthiolation. Compound **S3** was synthesized from **24** by installation of guanidine.

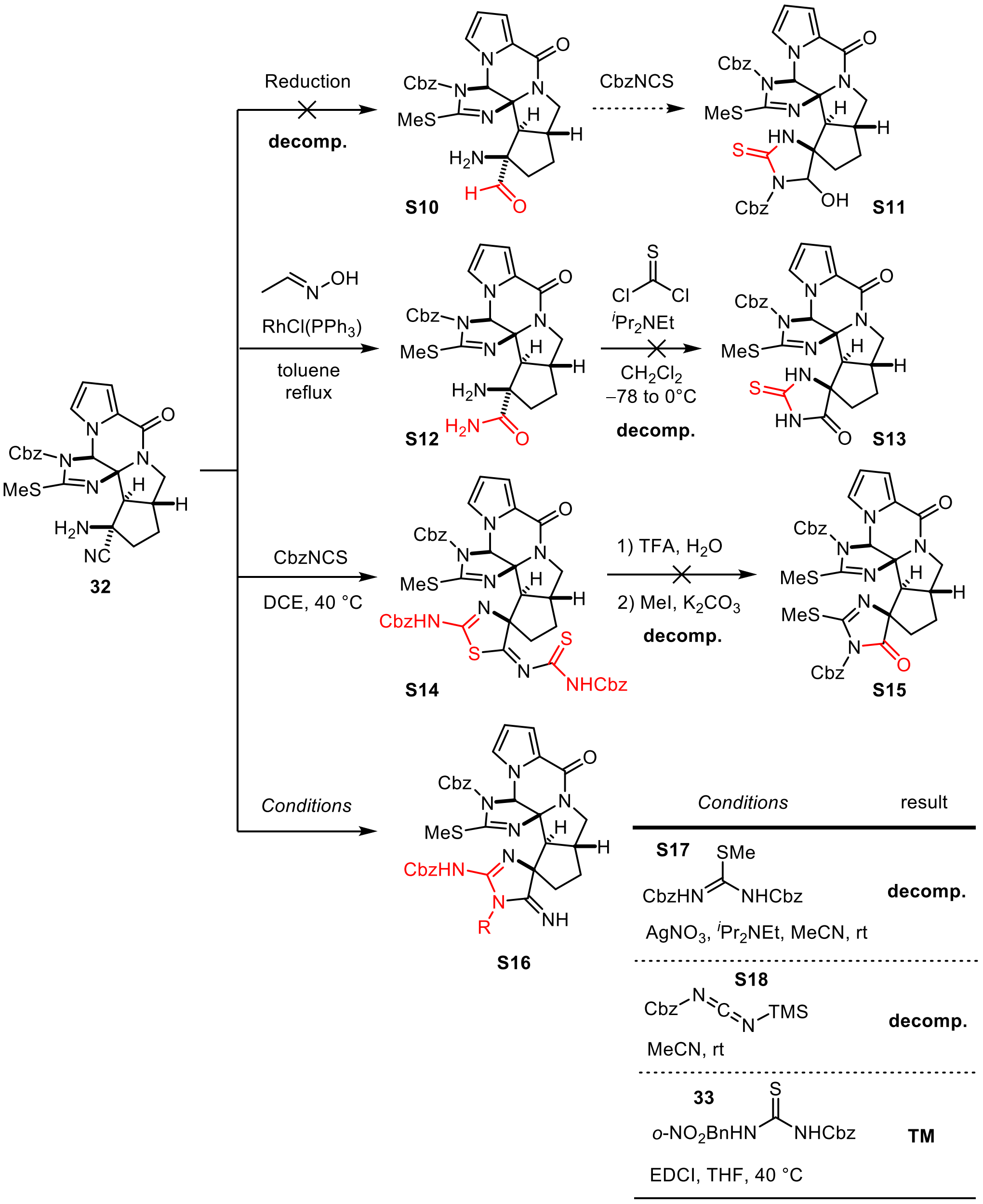

Scheme S3. Investigation for construction of the hexacyclic ring core. Hexacyclic compounds **S10**, **S12**, **S14** could not be synthesized through the reduction of nitrile, hydrolysis of nitrile, or installation of CbzNCS. Hexacyclic compound **S16** could be synthesized through only treatment with thiourea **33** and EDCI.

Experimental Detail

Compound (11)

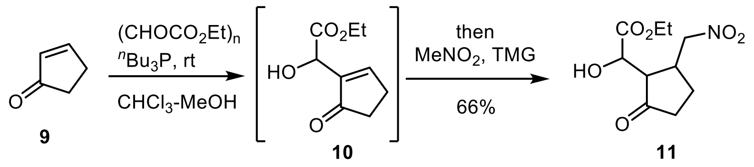

To a solution of cyclopentenone (**9**) (1.00 mL, 12.3 mmol) and 47% toluene solution glyoxylate (3.20 mL, 30.7 mmol) in $CHCl_3$ (30 mL) and MeOH (10 mL) was added dropwise $^{n}Bu_3P$ (0.30 mL, 1.2 mmol) at 0 °C, and the mixture was stirred for 45 minutes. To the mixture was added $^{n}Bu_3P$ (0.60 mL, 2.4 mmol) at 0 °C, and the mixture was stirred for 30 minutes. After the mixture was warmed up to room temperature, to the mixture were added nitromethane (10 mL) and tetramethyl guanidine (0.26 mL, 2.1 mmol) at room temperature, and the mixture was stirred for 45 minutes. To the mixture was added tetramethyl guanidine (0.26 mL, 2.1 mmol) at room temperature, and the mixture was stirred for 30 minutes. The reaction was quenched with saturated aqueous solution of NH_4Cl at 0 °C, and the mixture was extracted with AcOEt (x3). The combined organic layers were dried over anhydrous $MgSO_4$, filtered, and concentrated under reduced pressure. The residue was purified by silica gel column chromatography (Hexane/AcOEt = 2/1 to 1/1) to give **11** (1.99 g, 8.11 mmol, 66%) as a pale yellow oil. ^{1}H NMR (500 MHz, $CDCl_3$): δ 4.74 (dd, *J* = 5.3, 12.6 Hz, 1H), 4.51 (dd, *J* = 8.1, 12.6 Hz, 1H), 4.34 (d, *J* = 2.8 Hz, 1H), 4.33 (q, *J* = 7.2 Hz, 1H), 4.28 (q, *J* = 7.1 Hz, 1H), 3.18 (br s, 1H), 3.07-2.99 (m, 1H), 2.56 (dd, *J* = 1.7, 11.4 Hz, 1H), 2.42 (dd, *J* = 8.1, 18.4 Hz, 1H), 2.35-2.20 (m, 2H), 1.74-1.63 (m, 1H), 1.30 (t, *J* = 7.1 Hz, 3H); ^{13}C NMR (125 MHz, $CDCl_3$): δ 213.5, 172.9, 78.5, 68.4, 62.6, 54.8, 37.7, 37.1, 25.3, 14.2; IR (KBr): 3421, 2984, 1748, 11645, 1558, 1385 cm^{-1}; HRMS (ESI, m/z): $[M+H]^+$ calculated for $C_{10}H_{16}NO_6$, 246.0978; found, 246.0973.

Compound (14)

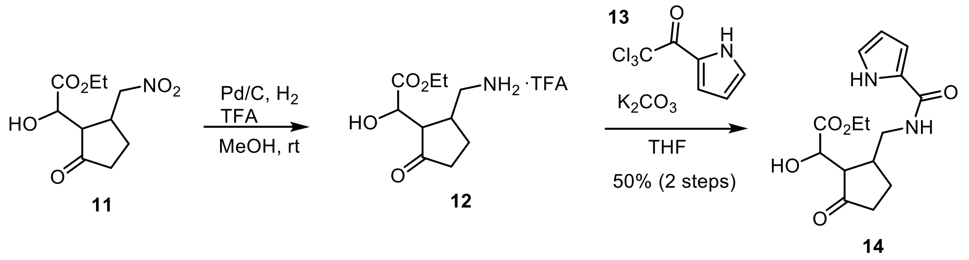

To a solution of **11** (2.1 g, 8.4 mmol) in MeOH (42 mL) were added 10% Pd/C (1.0 g) and TFA (1.90 mL, 25.4 mmol) at room temperature, and the mixture was heated to 40 °C under hydrogen atmosphere for 1 days. The mixture was filtered through Celite, and concentrated under reduced pressure to give crude **12**. The crude **12** was used for the next reaction without further purification.

To a solution of the crude **12** in THF (42 mL) were added K_2CO_2 (36.2mL, 260 mmol) and **13** (33.0 g, 173 mmol) at 0 °C, and the mixture was stirred at room temperature for 24 hours. The reaction was quenched with saturated aqueous solution of NH_4Cl at 0 °C, and the mixture was extracted with AcOEt (x3). The combined organic layers were washed with brine, dried over anhydrous $MgSO_4$, filtered, and concentrated under reduced pressure. The residue was purified by silica gel column chromatography (Hexane/AcOEt = 1/1 to 0/1) to give **14** (1.3 g, 4.2 mmol, 50%) as a pale yellow amorphous material. ^{1}H NMR (500 MHz, $CDCl_3$): δ 9.75 (br s, 1H), 6.92-6.91 (m, 1H), 6.77 (br t, *J* = 5.4 Hz, 1H), 6.63-6.61 (m, 1H), 6.21 (dt, *J* = 2.6, 3.6 Hz, 1H), 4.63 (s, 1H), 4.28 (dq, *J* = 7.2, 10.7 Hz, 1H), 4.19 (dq, *J* = 7.2, 10.7 Hz, 1H), 3.88 (br s, 1H), 3.85-3.80 (m, 1H), 3.41-3.36 (m, 1H), 2.48 (dd, *J* = 2.2, 11.0 Hz, 1H), 2.44-2.35 (m, 2H), 2.32-2.15 (m, 2H), 1.65-1.55 (m, 1H), 1.25 (t, *J* = 7.2 Hz, 3H); ^{13}C NMR (125 MHz, $CDCl_3$): δ 215.5, 173.2, 161.6, 125.8, 121.8, 110.0, 109.4, 69.3, 62.4, 56.8, 42.8, 39.9, 37.9, 25.4, 14.2; IR (KBr): 3379, 1739, 1621, 1566 cm^{-1}; HRMS (ESI, m/z): [M+Na]$^+$ calculated for $C_{15}H_{20}N_2NaO_5$, 331.1270; found, 331.1260.

Compound (17)

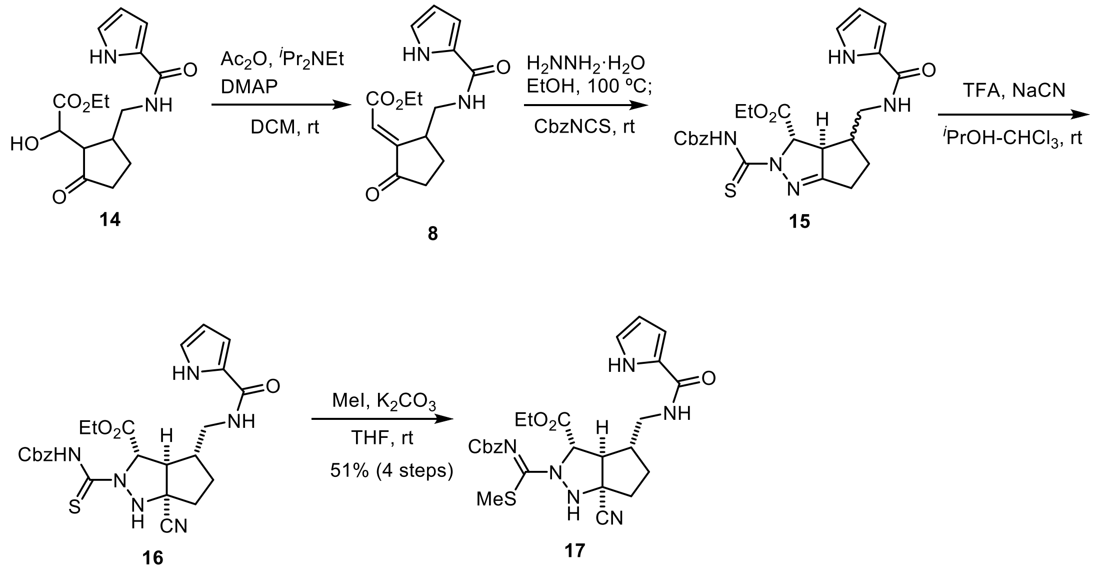

To a solution of **14** (11.6 g, 37.6 mmol) in CH_2Cl_2 (10.3 mL) were added ^{i}Pr_2NEt (10.3 mL, 113 mmol), Ac2O (4.3 mL, 45 mmol) and DMAP (459 mg, 3.80 mmol) at room temperature, and the mixture was stirred for 4 hours. The reaction was quenched with saturated aqueous solution of NH_4Cl at 0 °C, and the mixture was extracted with DCM (x3). The combined organic layers were dried over anhydrous $MgSO_4$, filtered, and concentrated under reduced pressure. The residue was passed through a short pad of silica gel to give the crude **8**. The crude **8** was used for the next reaction without further purification. To a solution of the crude **8** in EtOH (752 mL) was added $H_2NNH_2 \cdot H_2O$ (2.70 mL, 56.4 mmol) at room temperature, and the mixture was stirred at 100 °C for 1 hour. To the mixture was added CbzNCS (14.5 g, 75.2 mmol) at room temperature, and the

mixture was stirred at this temperature for 5 minutes. The mixture was filtered through Celite, and concentrated under reduced pressure. The residue was passed through a short pad of silica gel to give the crude **15**. The crude **15** was used for the next reaction without further purification. To a solution of the crude **15** in $CHCl_3$ (79 mL) and ^{i}PrOH (79 mL) were added NaCN (23.2 g, 474 mmol) and TFA (24.2 mL, 316 mmol) at 0 °C, and the mixture was stirred at room temperature for 2 hours. The reaction was quenched with saturated aqueous solution of $NaHCO_3$ at 0 °C, and the mixture was extracted with AcOEt (x3). The combined organic layers were dried over anhydrous $MgSO_4$, filtered, and concentrated under reduced pressure. A solution of the residue in AcOEt was washed with H_2O, and the aqueous layer was extracted with AcOEt (x3). The combined organic layers were dried over anhydrous $MgSO_4$, filtered, and concentrated under reduced pressure. The residue was passed through a short pad of silica gel to give the crude **16**. The crude **16** was used for the next reaction without further purification. To a solution of grinded K_2CO_3 (3.50 g, 25.1 mmol) and the crude **16** in THF (100 mL) was added MeI (1.60 mL, 25.1 mmol) at room temperature. The mixture was stirred at room temperature for 3.5 hours, and the reaction was quenched with saturated aqueous solution of NH_4Cl, and the mixture was extracted with AcOEt (x3). The combined organic layers were dried over anhydrous $MgSO_4$, filtered, and concentrated under reduced pressure. The residue was purified by silica gel column chromatography (Hexane/AcOEt = 1/2 to 1/9) to give **17** (10.2 g, 19.0 mmol, 51%) as a pale yellow amorphous material. ^{1}H NMR (500 MHz, $CDCl_3$): δ 9.27 (br s, 1H), 7.38-7.31 (m, 5H), 6.91-6.90 (m, 1H), 6.65-6.64 (m, 1H), 6.24 (dt, *J* = 2.6, 3.8 Hz, 1H), 6.13 (t, *J* = 6.4 Hz, 1H), 5.15 (br s, 1H), 5.06 (s, 2H), 4.63 (d, *J* = 1.4 Hz, 1H), 4.22 (dq, *J* = 7.2, 10.7 Hz, 1H), 4.14 (dq, *J* = 7.2, 10.7 Hz, 1H), 3.67-3.61 (m, 1H), 3.46-3.41 (m, 1H), 3.03 (dd, *J* = 1.2, 7.8 Hz, 1H), 2.48-2.41 (m, 1H), 2.27 (s, 3H), 2.13-2.08 (m, 1H), 2.02-1.92 (m, 2H), 1.59-1.48 (m, 1H), 1.21 (t, *J* = 7.1 Hz, 3H); ^{13}C NMR (125 MHz, $CDCl_3$): δ 169.0, 161.3, 136.3, 128.6, 128.5, 128.3, 125.6, 121.9, 119.5, 110.1, 109.5, 68.3, 68.0, 65.9, 62.7, 62.6, 47.5, 42.3, 38.3, 30.5, 15.2, 14.0 (two peaks missing); IR (KBr): 3350, 3249, 2977, 2936, 2246, 1738, 1630, 1563, 1529 cm^{-1}; HRMS (ESI, m/z): $[M+Na]^+$ calculated for $C_{26}H_{30}N_6NaO_5S_1$, 561.1896; found, 561.1893.

Compound (6)

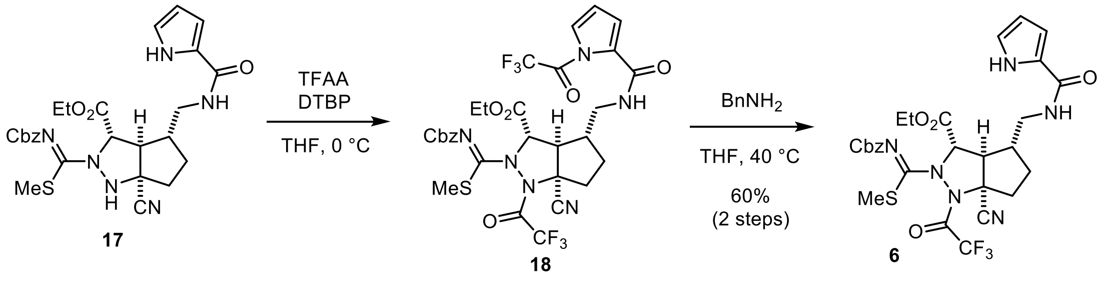

To a solution of **17** (10.2 g, 19.0 mmol) in THF (100 mL) were added DTBP (25.0 mL, 114 mmol) and TFAA (13.4 mL, 95.1 mmol) at 0 °C, and the mixture was stirred at room temperature for 25

minutes. The reaction was quenched with saturated aqueous solution of $NaHCO_3$ at 0 °C, and the mixture was extracted with AcOEt (x3). The combined organic layers were dried over anhydrous $MgSO_4$, filtered, and concentrated under reduced pressure. The residue was passed through a short pad of silica gel to give the crude **18**. The crude **18** was used for the next reaction without further purification. To a solution of the crude **18** in THF (281 mL) was added $BnNH_2$ (1.50 mL, 14.1 mmol) at room temperature, and the mixture was stirred at 40 °C for 24 hours. Additional $BnNH_2$ (0.42 mL, 3.9 mmol) was added to the mixture, and the mixture was stirred for 14 hours. Further $BnNH_2$ (0.10 mL, 0.94 mmol) was added to the mixture, and the mixture was concentrated under reduced pressure. The residue was purified by silica gel column chromatography (Hexane/AcOEt = 3/2 to 1/1 to 1/2) to give **6** (7.20 g, 11.4 mmol, 60%) as a pale yellow amorphous material. ^1H NMR (500 MHz, $CDCl_3$): δ 9.48 (br s, 1H), 7.37-7.32 (m, 5H), 6.94-6.93 (m, 1H), 6.62 (br s, 1H), 6.36 (t, *J* = 6.3 Hz, 1H), 6.24 (dt, *J* = 2.5, 3.7 Hz, 1H), 5.16 (s, 2H), 5.01 (s, 1H), 4.30-4.16 (m, 2H), 3.64-3.60 (m, 1H), 3.53-3.43 (m, 1H), 3.50 (d, *J* = 10.3 Hz, 1H), 2.80 (dd, *J* = 7.1, 14.3 Hz, 1H), 2.68 (dt, *J* = 6.7, 13.0 Hz, 1H), 2.28 (s, 3H), 2.21-2.09 (m, 2H), 1.72-1.63 (m, 1H); ^{13}C NMR (125 MHz, $CDCl_3$): δ 167.1, 166.3, 161.7, 158.5, 135.3, 128.7, 128.6, 128.5, 125.3, 122.2, 117.1, 115.3 (q, *J* = 286.4 Hz), 110.2, 109.6, 68.9, 68.7, 67.0, 63.1, 61.1, 45.0, 41.8, 38.7, 31.2, 15.2, 13.9 (one peak missing in $CDCl_3$) (one peak is broadened due to the rotamer); IR (KBr): 3388, 3259, 2960, 2250, 1738, 1711, 1631, 1562, 1524 cm^{-1}; HRMS (ESI, m/z): $[M+H]^+$ calculated for $C_{28}H_{30}F_3N_6O_6S_1$, 635.1900; found, 635.1905.

Note: The use of $BnNH_2$ was required to selectively remove the trifluoroacetyl group on the pyrrole ring. The selective removal did not proceed in acceptable yield by using methanol, isopropanol, and diethylamine.

Compound (19)

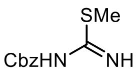

19

^1H NMR (400 MHz, $CDCl_3$): δ 7.42-7.28 (m, 5 H), 5.16 (s, 2H), 2.47 (s, 3H); ^{13}C NMR (100 MHz, $CDCl_3$): δ 174.2, 162.0, 136.7, 128.6, 128.3, 128.1, 67.4, 13.6; IR (KBr): 3383, 3289, 1660, 1594, 1497 cm^{-1}; HRMS (ESI, m/z): $[M+Na]^+$ calculated for $C_{10}H_{12}N_2NaO_2S_1$, 247.0517; found, 247.0523.

Compound (7')

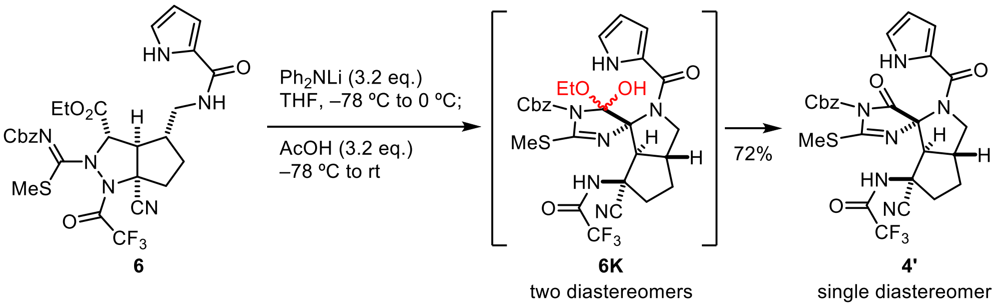

To a solution of **6** (800 mg, 1.26 mmol) in THF (42 mL) was slowly added 0.3 M THF solution of Ph_2NLi (13 mL, 3.9 mmol) at −78 °C while immersing the tip of the syringe needle in the solution, and the mixture was stirred for 10 minutes. The mixture was warmed up to 0 °C and the resulting orange solution was further stirred at this temperature for 15 minutes. After the mixture was cooled to −78 °C, the reaction was quenched with 0.5 M THF solution of AcOH (7.8 mL, 3.9 mmol). The mixture was warmed up to room temperature and stirred for 12 hours. The conversion of hemiorthoamide tetrahedral intermediate (**6K,** two diastereomers) to **7'** (single diastereomer) was checked by ^{19}F-NMR ($CDCl_3$) of the reaction solution (checking by TLC was difficult due to the similar behavior of **6K** and **4'** on the TLC). After brine was added to the mixture, the mixture was extracted with AcOEt (x3). The combined organic layers were dried over anhydrous $MgSO_4$, filtered, and concentrated under reduced pressure. The residue was purified by silica gel column chromatography (Hexane/AcOEt = 2/1 to 1/1, previously treated with *N*, *N*-dimethylaniline) to give **4'** (536 mg, 0.911 mmol, 72%) as a pale yellow amorphous. ^{1}H NMR (400 MHz, $CDCl_3$): δ 9.82 (br s, 1H), 7.56 (s, 1H), 7.42-7.41 (m, 2H), 7.38-7.34 (m, 3H), 6.96-6.95 (m, 1H), 6.64-6.63 (m, 1H), 6.24 (dt, *J* = 2.5, 3.8 Hz, 1H), 5.36 (d, *J* = 12.2 Hz, 1H), 5.30 (d, *J* = 12.2 Hz, 1H), 4.29 (dd, *J* = 6.3, 9.1 Hz, 1H), 3.69 (t, *J* = 9.2 Hz, 1H), 3.14-3.05 (m, 1H), 3.13 (d, *J* = 14.8 Hz, 1H), 2.93 (ddd, *J* = 3.4, 11.5, 15.2 Hz, 1H), 2.80 (ddd, *J* = 6.5, 8.8, 15.5 Hz, 1H), 2.47 (s, 3H), 2.00-1.93 (m, 1H), 1.74-1.65 (m, 1H); ^{13}C NMR (125 MHz, $CDCl_3$): δ 176.0, 164.9, 160.8, 156.7 (q, *J* = 38.8 Hz), 148.6, 133.9, 128.8, 128.7, 128.4, 123.4, 123.3, 116.5, 115.0 (q, *J* = 286.6 Hz), 114.2, 110.5, 81.9, 70.0, 63.9, 51.5, 49.4, 45.0, 43.4, 22.0, 14.7; IR (KBr): 3432, 3268, 2253, 1787, 1738, 1593, 1547 cm^{-1}; HRMS (ESI, m/z): $[M+H]^+$ calculated for $C_{26}H_{24}F_3N_6O_5S_1$, 589.1481; found, 589.1490.

Failed conditions except for Ph$_2$NLi

Base: LHMDS or KHMDS, Et$_2$NLi , LDA, ^{t}BuOLi

Temperature: –78 °C or –70 °C, –60 °C, –50 °C , –40 °C, –30 °C, 0 °C, rt

Eq. of Base: 1.05 or 2.05, 2.95, 3.05, 4.5, 1.05 + 2.0, 2.05 + 1.0

Solvent: THF or Et$_2$O, Toluene

Order to add Base: After or Before adding SM

Tool to add Base: Syringe or Teflon cannula

How to add Base: Add dropwise or Add along the wall, Add in the solv.

Additive: HMPA or H$_2$O

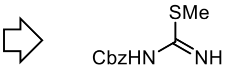

19

Major product under all conditions

a) ^{19}F-NMR (CDCl$_3$) of the reaction solution Immediately after quenching by AcOH

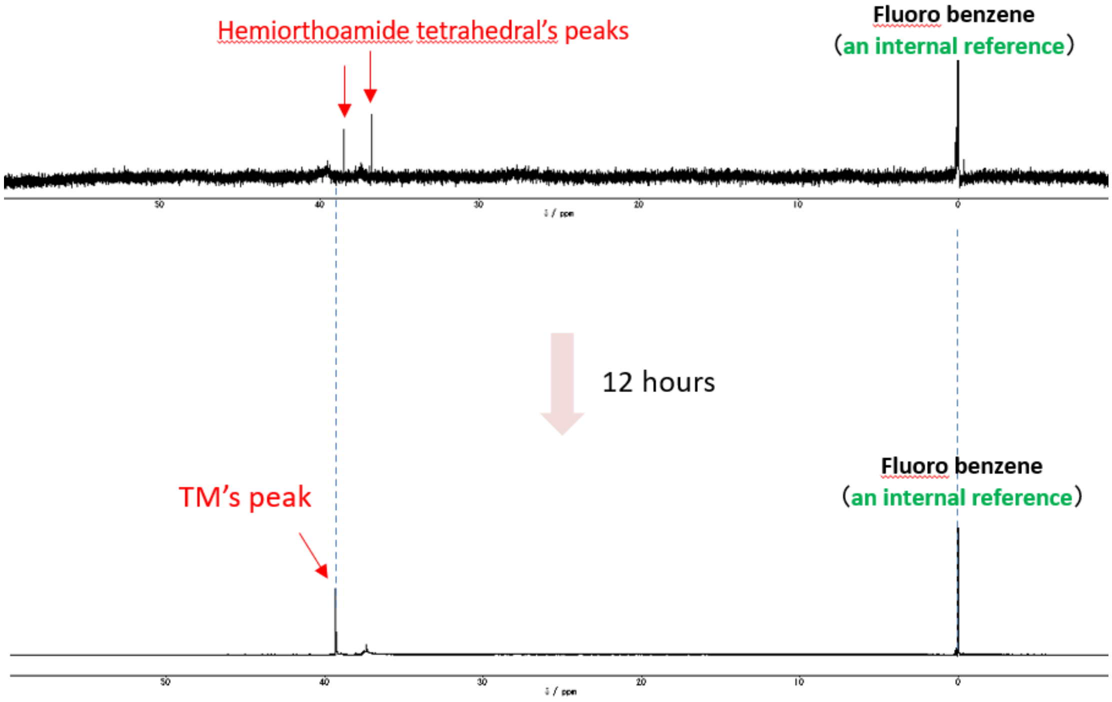

b) ^{19}F-NMR (CDCl$_3$) of the reaction solution after 12 hours

Preparation of Ph_2NLi

After addition of Ph_2NH (930 mg, 5.50 mmol) into the flask, argon gas was aerated inside the flask for 1minute. The Ph_2NH was dissolved in THF (13.7 mL), and to the mixture was slowly added 2.51 M hexane solution of ^{n}BuLi (2.0 mL, 5.0 mmol) at 0 °C, and the resulting pale yellow solution was stirred at this temperature for 45 minutes.

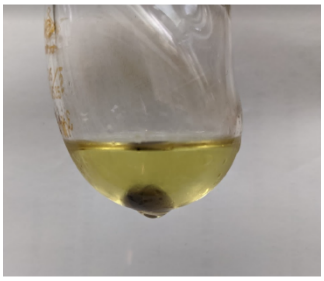

Activated Ph_2NLi
(Aeration with argon for 1 minute)
pale yellow solution

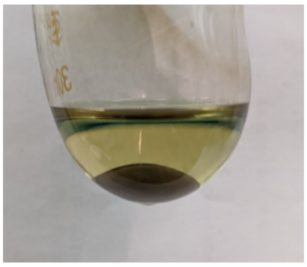

Deactivated Ph_2NLi
(No aeration with argon)
Pale green yellow solution

Compound (27)

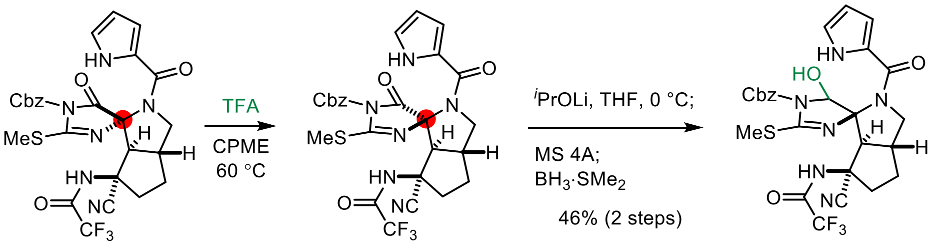

To a solution of **4’** (492 mg, 0.836 mmol) in cyclopentyl methyl ether (CPME) (28 mL) was added TFA (6.3 mg, 84 mmol) at room temperature, and the reaction was stirred at 60 °C for 1.5 hours. The reaction was quenched with saturated aqueous solution of $NaHCO_3$, and the mixture was extracted with AcOEt (x3). The combined organic layers were dried over anhydrous $MgSO_4$, filtered, and concentrated under reduced pressure. A solution of the residue in AcOEt was washed with H_2O, and the aqueous layer was extracted with AcOEt (x3). The combined organic layers were dried over anhydrous $MgSO_4$, filtered, and concentrated under reduced pressure. The crude **4** was used for the next reaction without purification. To a solution of the crude **4** in THF (17 mL) were added 0.2 M THF solution of LiO^{i}Pr (3.9 mL, 0.79 mmol) and HMPA (0.43 mL, 2.5 mmol) at 0 °C, and the mixture was stirred for 2 hours. To the mixture was added MS 4A, and the mixture was stirred for 10 minutes. To the mixture was added 0.5 M THF solution of $BH_3 \cdot SMe_2$ (0.50 mL, 0.99 mmol), and the mixture was stirred for 10 minutes. The reaction was quenched with saturated aqueous solution of NH_4Cl, and the mixture was extracted with AcOEt (x3). The combined organic layers were dried

over anhydrous $MgSO_4$, filtered, and concentrated under reduced pressure. The residue was purified by silica gel column chromatography (Hexane/AcOEt = 0/1) to give **27** (230 mg, 0.389 mmol, 46%) as an off-white amorphous material. ^{1}H NMR (500 MHz, $CDCl_3$): δ 9.39 (s, 1H), 7.39-7.31 (m, 5H), 7.13 (t, J = 1.7 Hz, 1H), 6.90 (dd, J = 1.5, 3.8 Hz, 1H), 6.24 (dd, J = 2.8, 3.6 Hz, 1H), 5.79 (d, J = 13.0 Hz, 1H), 5.69 (d, J = 13.0 Hz, 1H), 5.12 (s, 1H), 3.83 (dd, J = 7.6, 10.9 Hz, 1H), 3.23 (t, J = 10.5 Hz, 1H), 3.12 (dd, J =10.1, 15.5 Hz, 1H), 2.81 (dt, J = 8.9, 15.8 Hz, 1H), 2.61 (d, J = 14.0 Hz, 1H), 2.53-2.45 (m, 1H), 2.28 (s, 3H), 2.07-2.00 (m, 1H), 1.68-1.59 (m, 1H); ^{13}C NMR (125 MHz, $CDCl_3$): δ 158.0, 156.7 (q, J = 37.9 Hz), 153.4, 134.2, 129.3, 129.1, 129.0, 123.3, 122.3, 118.7, 115.4 (q, J = 284.2 Hz), 115.1, 151.9, 111.0, 83.9, 80.0, 69.0, 64.3, 50.2, 46.2, 45.7, 42.0, 24.9, 15.0; IR (KBr): 3330, 3210, 2247, 1726, 1620 cm^{-1}; HRMS (ESI, m/z): [M+H]$^+$ calculated for $C_{26}H_{26}F_3N_6O_5S_1$, 591.1637; found, 591.1623.

Compound (29)

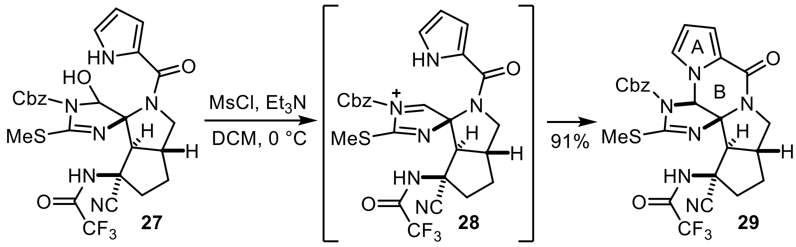

To a solution of **27** (419 mg, 0.709 mmol) in CH_2Cl_2 (14.2 mL) were simultaneously added dropwise MsCl (0.38 mL, 5.0 mmol) and Et_3N (0.99 mL, 7.1 mmol) at 0 °C,* and the mixture was stirred for 10 minutes. The reaction was quenched with saturated aqueous solution of $NaHCO_3$ at 0 °C, and the mixture was extracted with AcOEt (x3). The combined organic layers were dried over anhydrous $MgSO_4$, filtered, and concentrated under reduced pressure. The residue was purified by silica gel column chromatography (Hexane/AcOEt = 0/1) to give **29** (369 mg, 0.645 mmol, 91%) as an off-white amorphous material. ^{1}H NMR (500 MHz, CD_3CN): δ 7.54 (br s, 1H), 7.45 (d, J = 6.3 Hz, 2H), 7.40-7.34 (m, 3H), 6.98 (s, 1H), 6.75 (dd, J = 1.1, 3.7 Hz, 1H), 6.49 (s, 1H), 6.25 (t, J = 3.2 Hz, 1H), 5.38 (d, J = 11.9 Hz, 1H), 5.23 (d, J = 11.9 Hz, 1H), 3.97 (dd, J = 7.5, 10.2 Hz, 1H), 3.21 (t, J = 10.2 Hz, 1H) 3.19-3.09 (m, 1H), 2.99 (d, J = 13.9 Hz, 1H), 2.83 (ddd, J = 3.6, 11.5, 15.1 Hz, 1H), 2.77 (ddd, J = 6.9, 8.2, 15.1 Hz, 1H), 2.34 (s, 3H), 2.00-1.94 (m, 1H), 1.64 (ddd, J = 7.2, 11.3, 17.8 Hz, 1H); ^{13}C NMR (125 MHz, CD_3CN): δ 165.4, 157.1 (q, J = 38.1 Hz), 156.9, 150.7, 135.6, 129.7, 129.6, 125.2, 123.7, 118.7, 116.1 (q, J = 285.3 Hz), 113.8, 113.0, 87.5, 72.8, 70.5, 64.7, 64.6, 51.2, 47.1, 44.9, 41.0, 23.2, 15.5 (one peak missing); IR (KBr): 3341, 2246, 1730, 1643, 1553, 1511, 1502 cm^{-1}; HRMS (ESI, m/z): [M+Na]$^+$ calculated for $C_{26}H_{23}F_3N_6NaO_4S_1$, 595.1351; found, 595.1348.

*Note: Adding Et_3N before MsCl reduced the yield of **29**.

Compound (3)

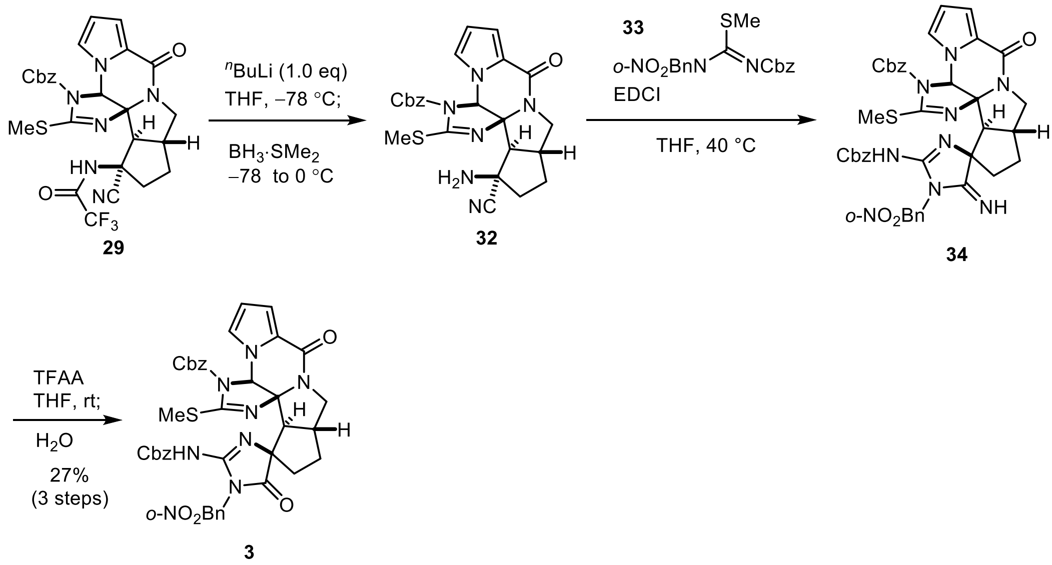

To a solution of **29** (102 mg, 0.177 mmol) in THF (3.6 mL) was added 2.51 M hexane solution of ^{n}BuLi at −78 °C, and the mixture was stirred for 15 minutes. To the mixture was added 2M THF solution of $BH_3·SMe_2$ (0.160 mL, 0.319 mmol), and the mixture was stirred for 15 minutes. The mixture was warmed up to 0 °C and further stirred at this temperature for 5 minutes. The reaction was quenched with AcOH (0.20 mL, 3.6 mmol). The mixture was warmed up to room temperature, and to the mixture was added MeOH (1.0 mL, 18 mmol). After the mixture was stirred for 1.5 hours, to the mixture was added H_2O (0.32 mL, 18 mmol), and the mixture was stirred for 1.5 hours. The reaction was quenched with saturated aqueous solution of $NaHCO_3$ at 0 °C, and the mixture was extracted with AcOEt (x3). The combined organic layers were dried over anhydrous $MgSO_4$, filtered, and concentrated under reduced pressure. The crude **32** was used for the next reaction without purification. To a solution of the crude **32** in THF (0.32 mL) were added thiourea **33** (220 mg, 0.637 mmol) and EDCI (122 mg, 0.637 mmol) at room temperature, and the mixture was stirred at 40 °C for 12 hours. After saturated aqueous solution of $NaHCO_3$ was added to the mixture at 0 °C, the mixture was extracted with AcOEt (x3). The combined organic layers were dried over anhydrous $MgSO_4$, filtered, and concentrated under reduced pressure. The crude **34** was used for the next reaction without purification. To a solution of the crude **34** in THF (12.7 mL) was added TFAA (0.21 mL, 1.5 mmol) at 0 °C, and the mixture was warmed up to room temperature mixture. After the mixture was stirred for 10 minutes, to the mixture was added H_2O (3.2 mL), and the mixture was stirred for 48 hours. The reaction was quenched with saturated aqueous solution of $NaHCO_3$ at 0 °C, and the mixture was extracted with AcOEt (x3). The combined organic layers were dried over anhydrous $MgSO_4$, filtered, and concentrated under reduced pressure. The residue was purified by silica gel column chromatography (Hexane/AcOEt = 1/2 to 1/4 to 0/1, previously treated with *N,N*-dimethylaniline) to give **3** (38 mg, 0.048 mmol, 27%) as a colorless oil**.** ^{1}H NMR (500 MHz,

$CDCl_3$): δ 8.62 (s, 1H), 8.15 (dd, J = 1.1, 8.2 Hz, 1H), 7.59 (t, *J* = 8.2 Hz, 1H), 7.52 (d, *J* = 6.6 Hz, 2H), 7.47 (t, *J* = 8.2 Hz, 1H), 7.42-7.35 (m, 5H), 7.32-7.27 (m, 3H), 7.05 (d, *J* = 8.2 Hz, 1H), 6.86 (dd, *J* = 1.7, 4.0 Hz, 1H), 6.68 (br s, 1H), 6.16 (br s, 1H), 6.05 (s, 1H), 5.56 (br s, 1H), 5.49 (br s, 1H), 5.29 (d, *J* = 17.0 Hz, 1H), 5,19 (d, *J* = 17.0 Hz, 1H), 5.14 (d, *J* = 12.6 Hz, 1H), 5.07 (d, *J* = 12.6 Hz, 1H), 4.16 (dd, *J* = 7.9, 10.9 Hz, 1H), 3.32 (t, *J* =10.3 Hz, 1H), 3.13-3.03 (m, 1H), 2.90 (ddd, *J* = 2.7, 11.1, 14.3 Hz, 1H), 2.65 (d, *J* = 14.4 Hz, 1H), 2.41 (dt, *J* = 8.2, 14.0 Hz, 1H), 2.21-2.14 (m, 1H), 1.98 (s, 3H), 1.69-1.58 (m, 1H); ^{13}C NMR (125 MHz, $CDCl_3$): δ 175.2, 164.0, 159.0, 156.4, 148.0, 136.8, 134.1×2, 131.0, 129.2×2, 129.0, 128.8, 128.6, 128.2, 128.0, 127.3, 125.8, 124.3, 113.8, 112.3, 77.7, 71.6, 70.1, 67.7, 64.0, 46.2, 45.2, 42.6, 40.5, 24.5, 15.3 (one peak missing in double bond region); IR (KBr): 3366, 2937, 1737, 1725, 1651, 1616, 1563, 1529 cm^{-1}; HRMS (ESI, m/z): [M+H]+ calculated for $C_{40}H_{37}N_8O_8S_1$, 789.2455; found, 789.2492.

Compound (35)

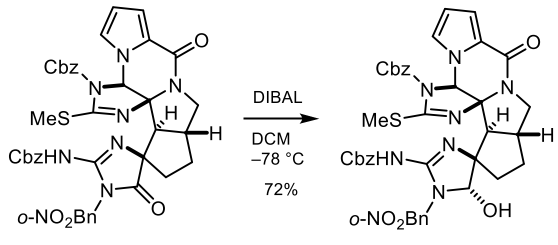

To a solution of **3** (6.8 mg, 8.6 μmol) in DCM (0.3 mL) was added 1M hexane solution of DIBAL (13 μL, 13 μmol) at −78 °C, and the mixture was stirred for 5 minutes. To the mixture was added 1M hexane solution of DIBAL (39 μL, 38.8 μmol), and the mixture was stirred for 10 minutes. To the mixture was added saturated aqueous solution of NH_4Cl, and the temperature was warmed to room temperature. After the mixture was diluted with Et_2O, the mixture was stirred for 30 minutes. To the mixture was added $MgSO_4$, and the mixture was stirred at room temperature for 30 minutes, filtered, and concentrated under reduced pressure. The residue was purified by silica gel column chromatography (Hexane/AcOEt = 9/1) to give **35** (4.9 mg, 6.2 μmol, 72%) as a white amorphous material. ^{1}H NMR (500 MHz, $CDCl_3$): δ8.10 (s, 1H), 7.95 (d, *J* = 8.0 Hz, 1H), 7.60 (t, *J* = 8.0 Hz, 1H), 7.48-7.38 (m, 4H), 7.35-7.32 (m, 2H), 7.29-7.22 (m, 6H), 6.93 (s, 1H), 6.74 (d, *J* = 2.4 Hz, 1H), 6.33 (br s, 1H), 6.17 (t, *J* = 3.4 Hz, 1H), 5.28 (d, *J* = 11.2 Hz, 1H), 5.15 (d, *J* = 12.9 Hz, 1H), 5.08-5.00 (m, 3H), 4.91 (d, *J* = 3.6 Hz, 1H), 4.56 (d, *J* = 16.2 Hz, 1H), 3.90 (t, *J* = 7.6 Hz, 1H), 3.02-2.89 (m, 2H), 2.34-2.26 (m, 1H), 2.19-2.13 (m, 1H), 2.08-2.00 (m, 1H), 2.04 (s, 3H), 1.89-1.86 (m, 1H), 1.07 (br s, 1H); ^{13}C NMR (125 MHz, $CDCl_3$): δ 164.6, 160.4, 157.0, 149.0, 137.9, 134.6, 134.0, 132.7, 129.1×2, 129.0×2, 128.9×2, 128.6, 128.4, 127.7, 127.6, 125.0, 123.5, 113.1, 112.2, 86.9, 77.7, 72.4, 69.3, 66.9, 66.7, 60.1, 48.0, 46.1, 42.2, 40.6, 24.1, 15.3, 134.8, 133.8, 133.3, 131.5,

130.8, 124.2, 117.4, 46.2, 43.2, 39.1, 32.5, 26.9 (one peak missing in double bond region); IR (KBr): 3372, 3237, 2928, 1727, 1650, 1593, 1566, 1525 cm^{-1}; HRMS (ESI, m/z): $[M+Na]^+$ calculated for $C_{40}H_{38}N_8NaO_8S_1$, 813.2431; found, 813.2448.

Compound (2)

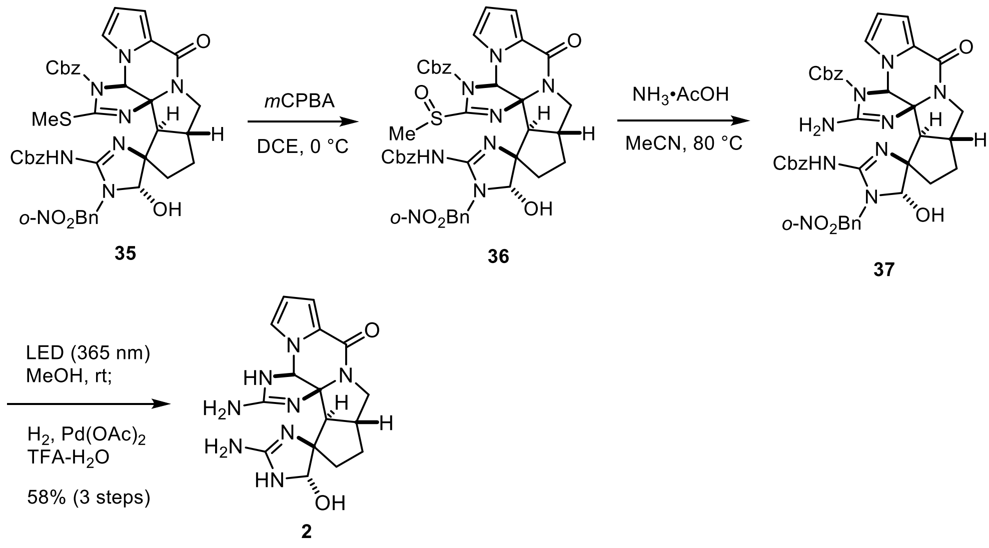

To a solution of **35** (5.8 mg, 7.3 μmol) in DCE (0.3 mL) was added 0.1 M DCM solution of *m*CPBA (0.10 mL, 11 μmol) at 0 °C, and the mixture was stirred for 1 hour. To the mixture was added 1M aqueous solution of Na_2SO_3 (20 μL), and the mixture was stirred for 10 minutes. To the mixture was added saturated aqueous solution of $NaHCO_3$. The mixture was extracted with AcOEt (x3). The combined organic layers were dried over anhydrous $MgSO_4$, filtered, and concentrated under reduced pressure. The residue was passed through aluminum oxide with AcOEt-MeOH (10:1) and concentrated under reduced pressure. The residue was dissolved in AcOEt, and the resulting solution was evaporated for azeotropic removal of the remaining MeOH. A solution of the residue in AcOEt was filtered through Minisart RC4 (pore size: 0.45 mm, filter diameter: 4 mm) to give the crude **36**. The crude **36** was used for the next reaction without purification. To a solution of the crude **36** in MeCN (0.15 mL) was added NH_3·AcOH (5.7 mg, 73 μmol) at room temperature, and the mixture was stirred at 80 °C for 13 hours. The mixture was concentrated under reduced pressure and repeated the evaporation in AcOEt for azeotropic removal of MeCN. A solution of the residue in AcOEt was filtered through Minisart RC4 (pore size: 0.45mm, filter diameter: 4 mm). The filtrate was concentrated under reduced pressure and repeated evaporation in MeOH. The crude **37** was used for the next reaction without purification. A solution of the crude **37** in MeOH (1.0 mL) was irradiated by PER-AMP (LED-UV, 365 nm) at room temperature in water bath (20 °C) covered by foil. After being stirred for 30 minutes, to the reaction mixture were added H_2O (0.5 mL), TFA (0.5 mL), and $Pd(OAc)_2$ (10 mg, 45 μmol). The hydrogen gas was bubbled through the mixture for 1 minute. After

being stirred under hydrogen atmosphere (balloon) at room temperature for 2 hours, the mixture was filtered through a cotton. The filtrate was concentrated under reduced pressure. The residue was purified by preparative HPLC (Hydrosphere C18, 12 mm, 250 × 10 mm, H_2O (with 0.1% TFA)/MeOH) linear gradient 99:1 to 60:40 in 30 min, flow rate 2.0 mL/min, t_R = 23.5 min) to afford **2・2TFA** (2.3 mg, 4.2 μmol, 58%) as a white amorphous material. ^{1}H NMR (500 MHz, CD_3OD): δ 7.05 (dd, *J* = 1.6, 2.7 Hz, 1H), 6.91 (dd, *J* = 1.5, 3.8 Hz, 1H), 6.40 (dd, *J* = 2.9, 3.8 Hz, 1H), 6.32 (s, 1H), 5.40 (s, 1H), 3.95 (dd, *J* = 7.0, 10.5 Hz, 1H), 3.19 (t, *J* = 10.5 Hz, 1H), 2.86-2.77 (m, 1H), 2.72 (d, *J* = 14.6 Hz, 1H), 2.66 (dd, *J* = 8.9, 14.8 Hz, 1H), 2.37-2.30 (m, 1H), 2.10 -2.05 (m, 1H), 1.52 (quint, *J* = 11.5 Hz, 1H); ^{13}C NMR (125 MHz, CD_3OD): δ 159.0, 158.6, 158.4, 123.8, 123.6, 114.8, 113.5, 87.9, 81.3, 70.0, 69.4, 60.2, 47.4, 46.9, 42.9, 25.2 (Peaks of CD_3CD_2OD contained in CD_3OD are mixed); IR (KBr): 3361, 2956, 2921, 2851, 1690, 1679, 1599, 1427, 1382 cm^{-1}; HRMS (ESI, m/z): $[M+Na]^+$ calculated for $C_{16}H_{21}N_8O_2$, 357.1787; found, 357.1779.

Determination of structure of 2:

The 2D-NMR charts including COSY, HMQC, HMBC and NOESY are put on S55-S61. The key correlations of NOESY are displayed in CD_3OD.

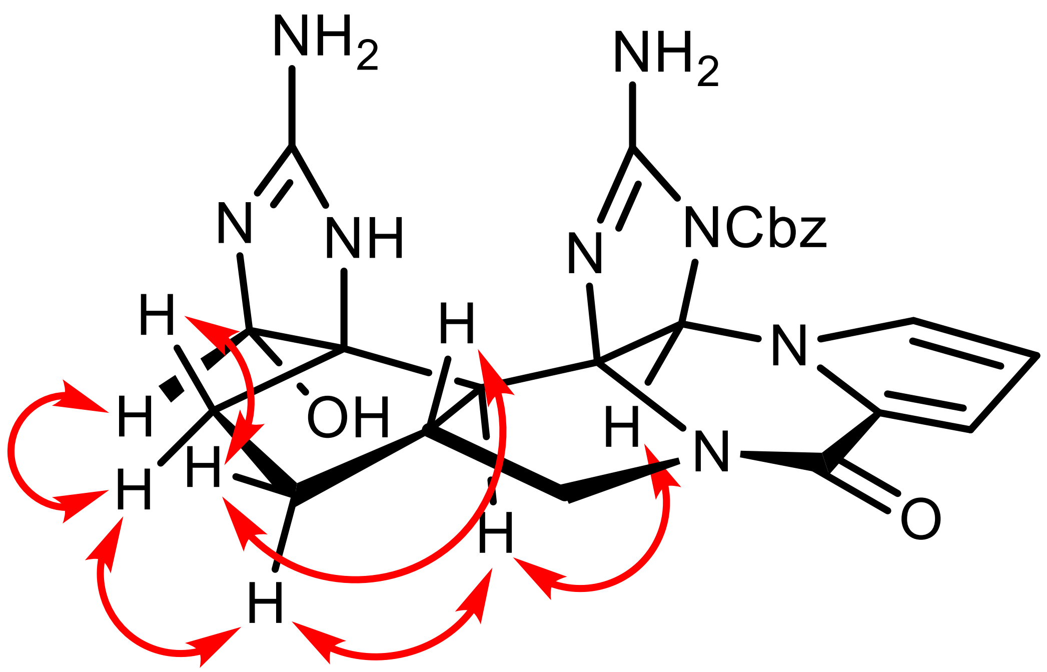

Chiral separation of 33 with chiral column:

3 (20 mg, 25 µmol) was separated by chiral column chromatography (CHIRALPAK IB, EtOH:*n*Hexane = 25:75) to give (–)-**3** (7.3 mg, 37%, tr = 17.2 min) and (+)-**3** (7.5 mg, 38%, t_R= 23.6 min) .

(–)-**3**: $[a]^{28}_D$ = –36.1 (*c* 0.5, $CHCl_3$)

(+)-**3**: $[a]^{28}_D$ = +38.1 (*c* 0.5, $CHCl_3$)

[Chiral HPLC analysis]

DAICEL, CHIRALPAK IB (4.6 x 250 mm)

eluent: EtOH:*n*Hexane = 25:75, flow rate; 1.0 mL/min, temperature 25 °C

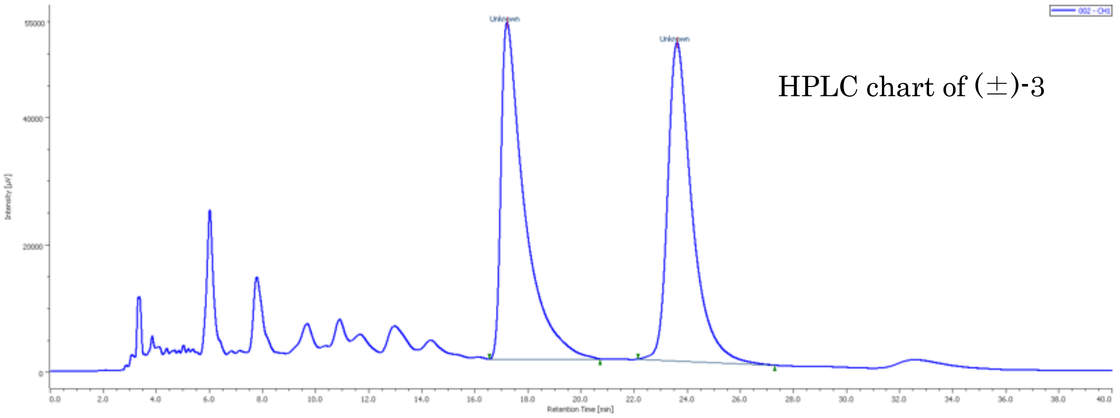

Compound	t_R	Area	Area (%)
(−)-3	17.21	3236318	50.396
(+)-3	23.608	3185399	49.604

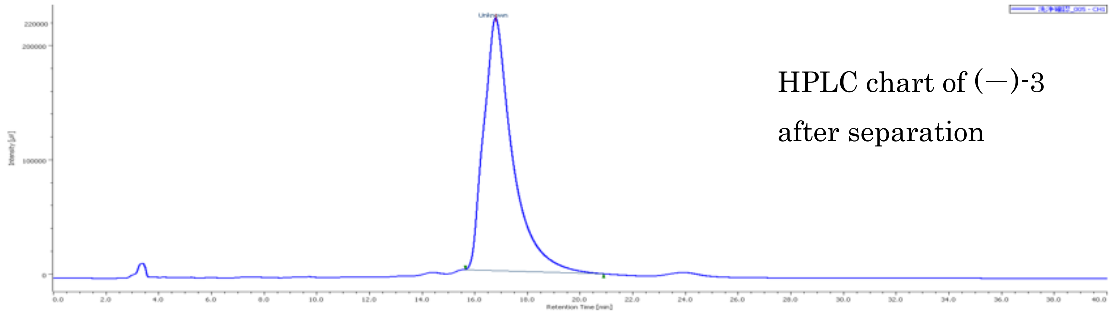

Compound	t_R	Area	Area (%)
(−)-3	16.788	16718078	100

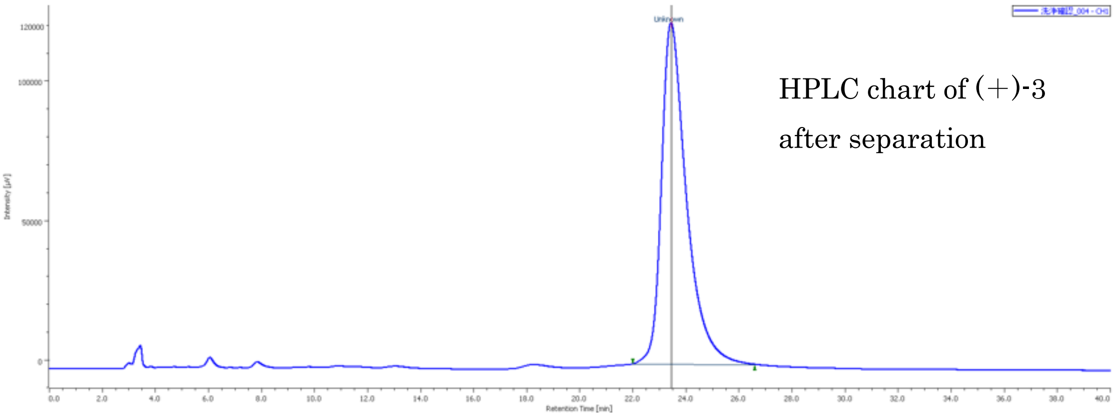

Compound	t_R	Area	Area (%)
(+)-3	23.433	8017777	100

(–)-**35**: Obtained from (–)-**3** (7.3 mg) in the manner similar to (±)-**35**.

$[a]^{28}_{D}$ = –19.0 (*c* 0.5, $CHCl_3$)

(+)-**35**: Obtained from (+)-**3** (7.5 mg) in the manner similar to (±)-**35**.

$[a]^{28}_{D}$ = +18.3 (*c* 0.5, $CHCl_3$)

(–)-**2**: Obtained from (–)-**35** (5.1 mg) in the manner similar to (±)-**2**.

$[a]^{28}_{D}$ = –34.8 (*c* 0.1, $CHCl_3$)

(+)-**2**: Obtained from (–)-**35** (5.2 mg) in the manner similar to (±)-**2**.

$[a]^{28}_{D}$ = +30.7 (*c* 0.1, $CHCl_3$)

Biological Test

Study for immunosuppressive activity of palau'amine analog 2

BALB/c mice (male, 5 weeks old) were purchased from Japan SLC (Shizuoka, Japan). The experimental animals were allowed free access to water and mouse chow, and were housed under controlled environmental conditions (constant temperature, humidity, and a 12-h dark–light cycle). The animal experiment was approved in advance by the Animal and Ethics Review Committee of Tokushima University (Approval No. T2019-47).

Splenic lymphocytes were prepared as previously described.[1] Spleens were collected from mice and suspended into RPMI-1640 medium by being pressed through a 100 µm cell strainer (Greiner Bio-One, Kremsmünster, Austria). The cell suspension was centrifuged at 300 *xg* for 5 min, and the cell pellet was resuspended in an ammonium chloride lysis buffer (0.83% NH_4Cl) followed by 3-min incubation to completely lyse the red blood cells. The resultant splenic lymphocytes were seeded onto a well of a 96-well plate (4×10^5 cells/well) and incubated at 37 °C for 1 h. The cells were further incubated with an aqueous solution of (-)-**2** (100 µM), (+)-**2** (100 µM), (±)-**2** (100 µM), or cyclosporine A (100 µM, FUJIFILM Wako Pure Chemical Corp., Osaka, Japan) at 37 °C for 1 h. To induce the interleukin-2 (IL-2) secretion, the cells were stimulated by incubation with phorbol 12-myristate 13-acetate (PMA, 10 nM, FUJIFILM Wako Pure Chemical Corp., Osaka, Japan) and lectin from Phaseolus vulgaris (PHA-E4, 1.0 µg/mL, J-Oil Mills, Tokyo, Japan).[2,3] After 24-h incubation, IL-2 levels in the culture supernatant were determined using an ELISA kit (M2000, R&D systems, Minneapolis MN, USA).

References

1. T. Shimizu, A.S. Abu Lila, Y. Kawaguchi, Y. Shimazaki, Y. Watanabe, Y. Mima, Y. Hashimoto, K. Okuhira, G. Storm, Y. Ishima, T. Ishida. A Novel Platform for Cancer Vaccines: Antigen-Selective Delivery to Splenic Marginal Zone B Cells via Repeated Injections of PEGylated Liposomes. *J. Immunol.* **201** (2018) 2969-2976.

2. K. Namba, K. Takeuchi, Y. Kaihara, M. Oda, A. Nakayama, A. Nakayama, M. Yoshida, K. Tanino. Total synthesis of palau'amine, *Nat. Commun.* **6** (2015) 8731.

3. A. Granelli-Piperno, P. Nolan, Nuclear transcription factors that bind to elements of the IL-2 promoter. Induction requirements in primary human T cells. *J. Immunol.* **147** (1991) 2734-2739.

Computational details

Density functional theory calculations were conducted employing the Gaussian 09 program package[1]. The geometry of the molecules considered were fully optimized in the gas phase at DFT level with B3LYP functional[2] in combination with 6-31G (d,p) basis set. We use the M062x/6-311g(d) method for single-point and frequency calculations. Electronic energies with zero point corrections were used to calculate relative energies and compare the stabilities.

For the compound (**4**)

Sum of electronic and zero-point Energies= -2406.488538 (-1.3 kcal/mol)

0 1

C	2.77061000	3.21896300	-0.40804800
C	2.99313400	-0.45763300	-0.78415600
H	2.75522300	3.62339100	-1.42729100
H	3.18460800	3.98341100	0.25173900
C	1.04821000	1.46068600	-0.19781600
C	4.65308100	1.41951100	-1.17404300
H	4.65788600	1.94113600	-2.13682700
H	5.61379200	1.61077500	-0.69074400
C	4.41377500	-0.11666800	-1.39201000
H	5.18231900	-0.71609200	-0.91226900
H	4.41843200	-0.37211600	-2.45415100
C	2.35810400	0.95660500	-0.86202700
H	2.24194900	1.17191300	-1.93241700
C	3.45564500	1.86638600	-0.32979300
H	3.63830800	1.62496400	0.72358700
N	1.39223100	2.87879500	0.04425500
C	0.47648000	3.94533900	0.02737300
C	-2.02545800	4.41196500	0.38960700
C	-2.98479500	3.97129900	1.32996800
H	-2.15998700	5.12681800	-0.40858300
C	-2.35503300	3.06584700	2.16884600
H	-4.01676200	4.28455900	1.39947400
H	-2.72439000	2.51877300	3.02346700
O	0.80930500	5.02596700	-0.44229900
C	-0.83588300	3.74931500	0.66039000

N	-1.06185800	2.93220500	1.75343500
H	-0.35170100	2.36696800	2.19563800
C	-0.14440500	1.28137000	-1.16504600
O	-0.29725300	1.78928900	-2.24159700
C	2.20359400	-1.41845700	-1.58458700
N	1.51199700	-2.14321700	-2.17182700
N	0.70626500	0.65351200	0.97670000
C	-0.40818800	0.04638200	0.73885100
N	-1.01507300	0.37192700	-0.49669200
S	-1.00840600	-1.14407700	1.89313900
C	-1.66359700	-2.47152700	0.80722800
H	-0.99033600	-2.64096200	-0.03450300
H	-1.66893100	-3.36324700	1.43696700
H	-2.67584400	-2.25621300	0.47062000
N	3.05191000	-0.89330700	0.62288900
H	2.33417800	-0.45837000	1.21027300
C	3.58608700	-1.99687600	1.23162300
O	3.34352400	-2.27817600	2.39064800
C	4.60682700	-2.88897500	0.47790100
F	4.31106300	-3.05261300	-0.83129400
F	4.66319600	-4.09256900	1.04121500
F	5.83994000	-2.33134500	0.54786000
C	-2.37288200	0.24582800	-0.92526200
O	-3.19719300	0.20480900	0.12671400
O	-2.67944700	0.24254400	-2.08873800
C	-4.63323700	0.18692200	-0.18134200
H	-5.07308500	0.71115600	0.66760600
H	-4.78458700	0.76607600	-1.09286300
C	-5.17346600	-1.21264900	-0.30881300
C	-5.14302900	-1.88093900	-1.54175100
C	-5.71109100	-1.86524500	0.80874100
C	-5.63917800	-3.17980700	-1.64941800
H	-4.72297600	-1.37982100	-2.40802900
C	-6.20867800	-3.16395500	0.70000000
H	-5.74388200	-1.35059200	1.76580500
C	-6.17245000	-3.82271300	-0.53020100

H	-5.61294100	-3.68857800	-2.60830600
H	-6.62736600	-3.65806300	1.57162300
H	-6.56205300	-4.83264100	-0.61752600

For the compound (**4’**)

Sum of electronic and zero-point Energies = -2406.487174 (0 kcal/mol)

0 1

C	-2.79854500	-3.19552500	-0.02981000
C	0.67420200	-2.12373000	0.72821300
H	-3.18911600	-3.52489500	0.94112000
H	-3.33624700	-3.73647400	-0.81040800
C	-1.85193200	-0.93522000	0.23628300
C	-0.45130000	-4.39297600	0.58391000
H	-0.99543600	-4.80168400	1.44153800
H	-0.21460700	-5.22627900	-0.08119900
C	0.84671000	-3.65910900	1.07333200
H	1.74240200	-4.04667200	0.59260400
H	0.98151600	-3.77163800	2.15144300
C	-0.87914400	-2.06738800	0.69031800
H	-1.17560600	-2.23539300	1.73333400
C	-1.28242100	-3.30085400	-0.10244500
H	-0.96977200	-3.18567600	-1.14680600
N	-3.00462300	-1.73555300	-0.22041300
C	-4.33563600	-1.29062000	-0.14517900
C	-5.61896400	0.93979700	-0.13297900
C	-5.52562700	2.13463900	-0.88871800
H	-6.31379500	0.71322600	0.66214100
C	-4.48850500	1.97270800	-1.79065000
H	-6.15034200	3.01165200	-0.79615800
H	-4.11703300	2.62787700	-2.56466600
O	-5.22242900	-2.06306100	0.19410500
C	-4.62171900	0.08575200	-0.57443500
N	-3.93287700	0.73980800	-1.58280400
H	-3.25409800	0.29979200	-2.18833900
C	-1.21286500	-0.02665900	-0.81997200
O	-0.97156200	-0.29654600	-1.98230800

C	1.17241900	-1.21979000	1.78604300
N	1.49944400	-0.48502700	2.62267700
N	-2.14015700	-0.04613300	1.36603200
C	-1.59421300	1.08799500	1.13714900
N	-0.93235400	1.16126200	-0.14464000
S	-1.57843100	2.38309500	2.33907400
C	-2.56436600	3.68377500	1.49045200
H	-3.53573200	3.28639800	1.19288600
H	-2.70386300	4.46032200	2.24569400
H	-2.01613000	4.08188600	0.63932100
N	1.26157100	-1.78391500	-0.58182500
H	0.64928400	-1.33887500	-1.26133900
C	2.55177300	-1.72026900	-1.02659600
O	2.83744400	-1.23777900	-2.10956400
C	3.70635600	-2.36721600	-0.21802000
F	3.54110800	-2.32729400	1.11918300
F	4.85723100	-1.75957200	-0.51010200
F	3.80997300	-3.66903500	-0.57283700
C	-0.01028800	2.15410800	-0.60312600
O	0.97262000	1.55211100	-1.25982100
O	-0.13592400	3.33663100	-0.39262400
C	2.01260300	2.40291700	-1.85910900
H	2.31905200	1.82906700	-2.73349400
H	1.54561100	3.34050700	-2.16390000
C	3.15535400	2.62296100	-0.90578300
C	3.18647500	3.75379900	-0.07957400
C	4.19203700	1.68311400	-0.82562900
C	4.24082700	3.94273400	0.81340700
H	2.37706500	4.47562200	-0.13101900
C	5.24207400	1.87102200	0.07234100
H	4.17165200	0.80320400	-1.46183100
C	5.26872600	3.00136500	0.89143600
H	4.25839100	4.82248500	1.44978600
H	6.03653000	1.13318000	0.13198200
H	6.08795700	3.14805400	1.58927400

The geometry optimization in solvent THF was conducted at DFT level with B3LYP functional2 in combination with 6-31+G (d,p) basis set with the self-consistent reaction field (SCRF) polarized continuum model (IEFPCM) [3]. Frequency calculations were performed to confirm the ground state geometry of calculated structures as zero imaginary frequency. The stability were compared from the relative energies of the molecules obtained from zero point corrected electronic energy.

For the intermediate (**21**)
Sum of electronic and zero-point Energies = -2413.629900 Hartree (0 kcal/mol)
0 1

C	-3.93115300	2.19551100	-0.09446100
C	-3.04840400	-1.20631300	1.09269800
H	-4.27396400	2.70501800	0.81328700
H	-4.41913200	2.65722500	-0.95540500
C	-1.71821000	1.09898000	0.12879500
C	-5.24730300	0.03022500	0.78571900
H	-5.63955700	0.71498200	1.54500500
H	-6.07892300	-0.25853300	0.13890600
C	-4.59395000	-1.21270300	1.47278200
H	-5.01263600	-2.16027200	1.13121000
H	-4.71516900	-1.16053000	2.55806700
C	-2.87161000	0.31586600	0.82240300
H	-2.97039000	0.80772800	1.80104600
C	-4.10336100	0.68860200	0.00543600
H	-4.01708400	0.23749300	-0.98820300
N	-2.45243600	2.33517500	-0.23858500
C	-1.94493500	3.62216700	-0.29961100
C	0.31848100	4.85576100	-0.37907900
C	1.46735600	4.74623300	-1.19999900
H	0.13597600	5.57980300	0.40228900
C	1.25694400	3.67991200	-2.06354800
H	2.34961500	5.37031200	-1.16940800
H	1.87227600	3.27714400	-2.85450600
O	-2.68062600	4.58618000	-0.05041500
C	-0.55746300	3.83843400	-0.74360200

N	0.04134300	3.13147700	-1.77125000
H	-0.40304200	2.38554000	-2.28905600
C	-0.56241600	1.31096700	1.13075200
O	-0.58870300	1.89202800	2.19096700
C	-2.29349900	-1.64177600	2.29321300
N	-1.77007200	-2.00124800	3.26960200
N	-1.11466500	0.40235900	-1.00591500
C	0.11815500	0.16911400	-0.73173400
N	0.53226900	0.63628700	0.55387800
S	1.17387700	-0.59000500	-1.92669700
C	2.06327300	-1.87136000	-0.96239400
H	1.33951400	-2.47961600	-0.41883900
H	2.55906300	-2.48258100	-1.71945700
H	2.81930300	-1.44011700	-0.30788700
N	-2.87002900	-2.05077500	-0.07490700
C	-1.82444800	-2.80268100	-0.20821400
C	-1.86962400	-3.72509200	-1.44897600
F	-2.80192000	-3.39836800	-2.36602100
F	-2.13198900	-5.00903300	-1.05968900
F	-0.67634500	-3.75851100	-2.09280500
C	1.82810500	0.72345700	1.15117600
O	2.74089900	1.11292000	0.26937200
O	1.99758000	0.50655500	2.32966000
C	4.11476900	1.33604200	0.76708200
H	4.46251600	2.16080500	0.14557000
H	4.04541800	1.65462000	1.80682600
C	4.97782000	0.11408800	0.60240100
C	5.14571700	-0.79357500	1.65935400
C	5.63345300	-0.12507800	-0.61493300
C	5.94761500	-1.92667200	1.49748100
H	4.64917700	-0.60924700	2.60754800
C	6.43353500	-1.25810400	-0.77846500
H	5.51860500	0.57977600	-1.43426900
C	6.59040600	-2.16176500	0.27787900
H	6.07510800	-2.62053800	2.32315900
H	6.93814000	-1.43205800	-1.72419200

H	7.21655300	-3.04034400	0.15321300
O	-0.77965300	-2.96316500	0.51328700
Li	-0.26582900	-3.25460700	2.26774300

For the intermediate (**22**)

Sum of electronic and zero-point Energies = -2413.634778 Hartree (-3.06 kcal/mol)

0 1

C	-3.07259000	3.03543900	0.28262900
C	-2.95394400	-0.54361700	1.23441300
H	-3.09294100	3.58650700	1.22952800
H	-3.56309400	3.64181200	-0.48082300
C	-1.19153500	1.42352200	0.25762300
C	-4.75801100	1.23533700	1.37927500
H	-4.80814100	1.92939600	2.22496000
H	-5.73395800	1.24162500	0.88809300
C	-4.36680200	-0.19780800	1.87605800
H	-5.08371600	-0.96338500	1.58440300
H	-4.29215200	-0.22331400	2.96619800
C	-2.42978500	0.90556300	1.04896600
H	-2.29729000	1.31406600	2.05998400
C	-3.62007000	1.62899500	0.43002100
H	-3.81832500	1.21411100	-0.56452200
N	-1.66210400	2.77407000	-0.13620800
C	-0.85819300	3.89621300	-0.31801300
C	1.60508500	4.52606800	-0.71905100
C	2.59135800	4.10901300	-1.64498700
H	1.69578500	5.29364900	0.03623900
C	2.03004900	3.10457200	-2.42123000
H	3.59708100	4.49378000	-1.73992800
H	2.43433200	2.53953800	-3.24816400
O	-1.29088800	5.01413000	-0.01871000
C	0.47047800	3.74960400	-0.93329600
N	0.75813900	2.88494100	-1.97517200
H	0.09079900	2.26107100	-2.40555500
C	0.05065800	1.46083400	1.17489800
O	0.20785100	2.11510600	2.17471900

C	-2.09611300	-1.29526200	2.17962800
N	-1.37578300	-1.85186000	2.90295500
N	-0.80212900	0.52942400	-0.84040400
C	0.38015600	0.06423800	-0.59600600
N	0.96782600	0.55400800	0.58800400
S	1.09826700	-1.09674300	-1.71234000
C	1.78011600	-2.39762600	-0.60903400
H	1.05750000	-2.66358200	0.16235400
H	1.93173500	-3.25419500	-1.26856100
H	2.73465800	-2.09922900	-0.18046400
N	-3.07204700	-1.25639500	-0.04289300
C	-3.63392100	-2.45414500	0.02728400
O	-4.05591100	-3.11582800	0.99575900
C	-3.80150600	-3.14407400	-1.35245400
F	-5.10353700	-3.33068600	-1.65752100
F	-3.20160600	-4.35229600	-1.38677600
F	-3.26555000	-2.42485400	-2.40297500
C	2.31406200	0.45742100	1.07570900
O	3.18243900	0.45847900	0.07167400
O	2.55458900	0.43540500	2.25811400
C	4.62186500	0.44995100	0.42184700
H	5.06121400	1.07785400	-0.35264300
H	4.72813900	0.92632500	1.39587700
C	5.19005500	-0.94256400	0.40055400
C	5.22456400	-1.72068100	1.56819500
C	5.69689000	-1.47736100	-0.79370200
C	5.74764200	-3.01597300	1.53817900
H	4.83986700	-1.31129200	2.49765400
C	6.22019100	-2.77206700	-0.82438300
H	5.68552200	-0.87634800	-1.69929100
C	6.24423600	-3.54385500	0.34201600
H	5.77049100	-3.60967300	2.44705700
H	6.61282600	-3.17510400	-1.75320300
H	6.65330200	-4.54964400	0.32005900
Li	-2.14374400	-0.74120800	-1.72453100

References

1. M. J. Frisch, et al., Gaussian 09, Gaussian, Inc., Wallingford, CT, 2009.

2. (a) A. D. Becke J. Chem. Phys., 1993, 98, 5648-5652.

(b) C. T. Lee, W. T. Yang, R. G. Parr, Phys. Rev. B 1988, 37, 785-789

3. J. Tomasi, B. Mennucci and R. Cammi, Chem. Rev., 2005, 105, 2999-3093.)

4. J. Phys. Chem. B, 113 (2009) 6378-96. DOI: 10.1021/jp810292n

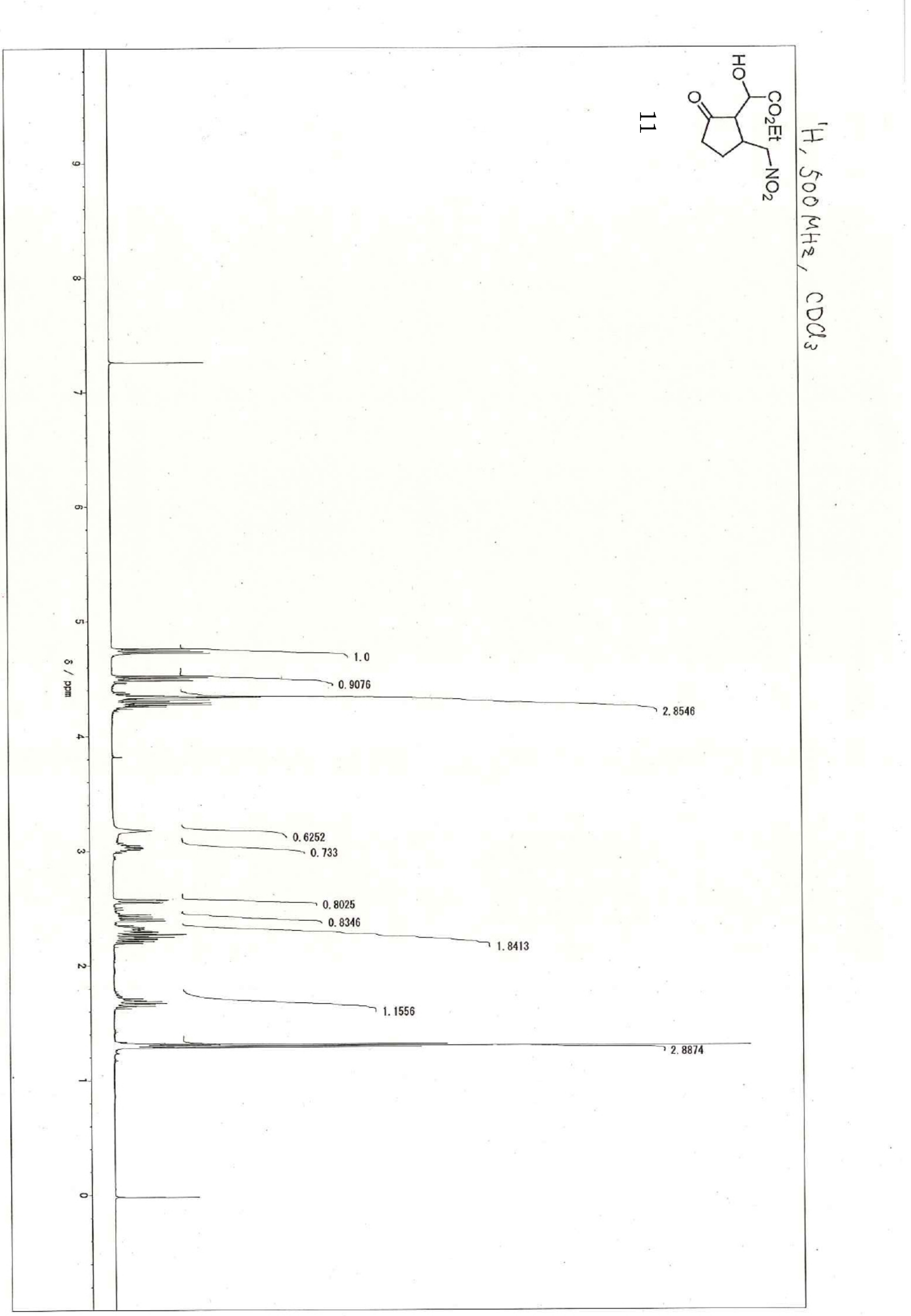
1H, 500 MHz, CDCl3
11
HO
CO2Et
NO2
δ / ppm
1.0
0.9076
2.8546
0.6252
0.733
0.8025
0.8346
1.8413
1.1556
2.8874

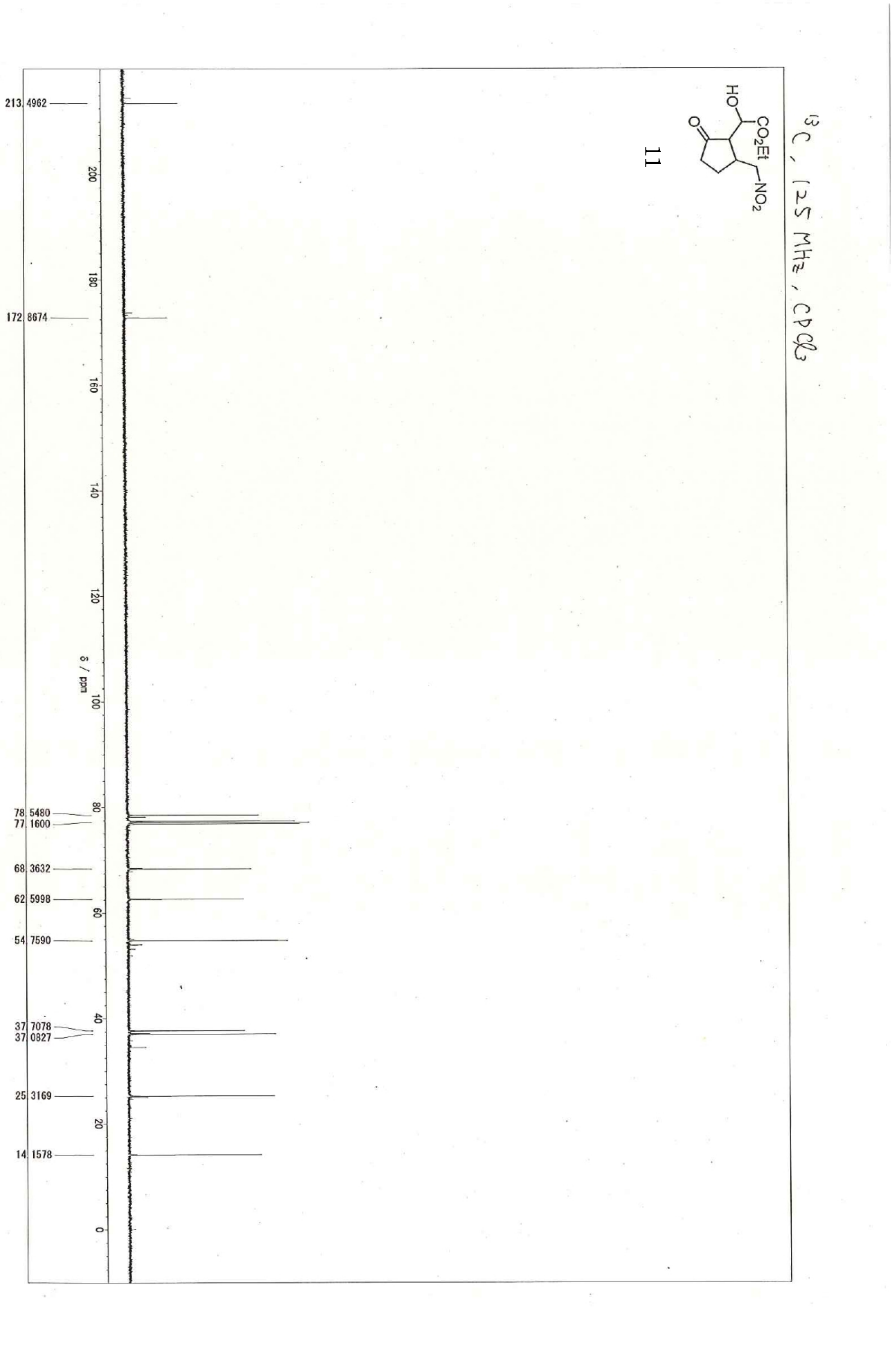
13C, 125 MHz, CDCl3
11
HO
CO2Et
NO2
O
213.4962
172.8674
78.5480
77.1600
68.3632
62.5998
54.7590
37.7078
37.0827
25.3169
14.1578
δ / ppm

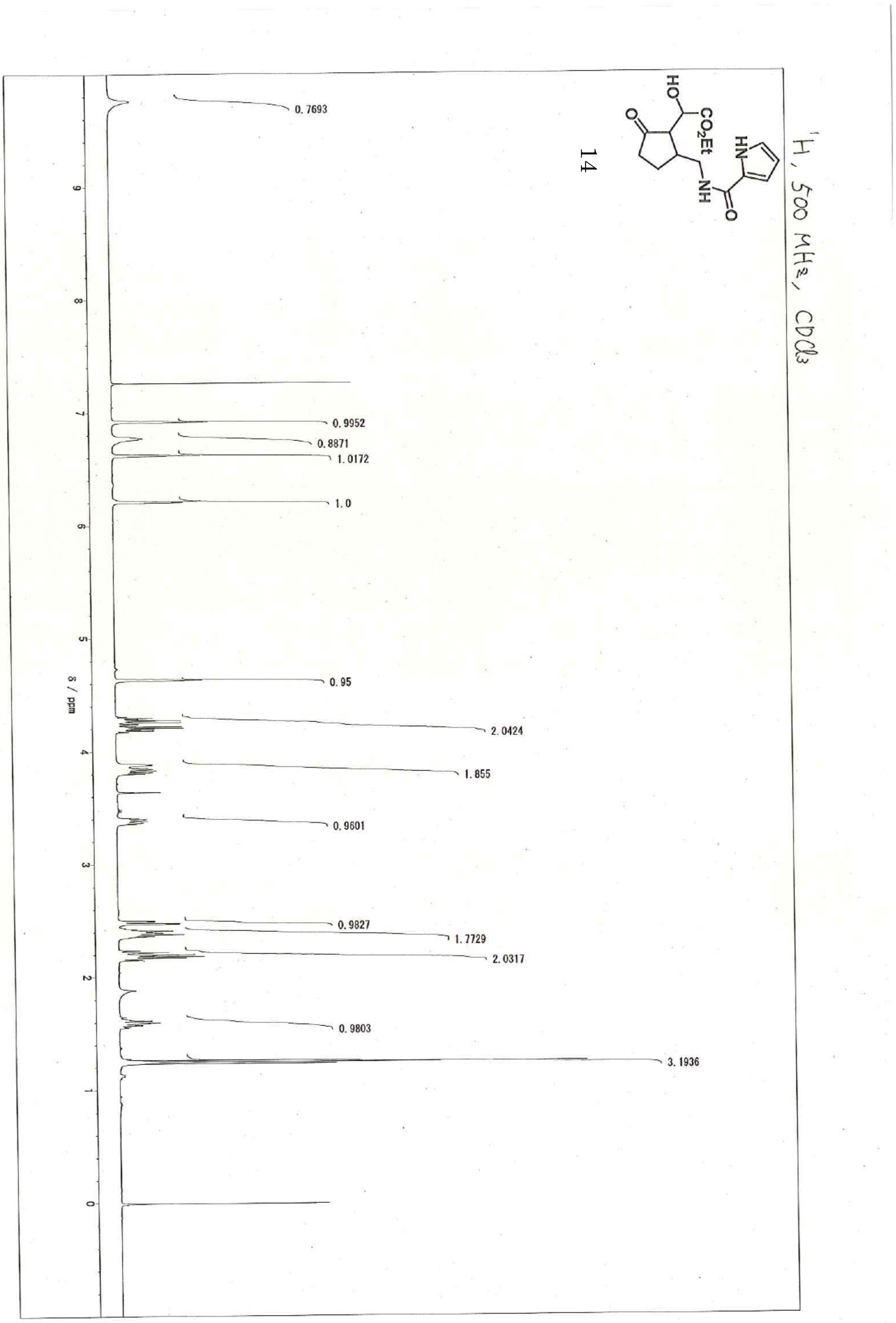
1H, 500 MHz, CDCl3
14
HN
NH
O
HO
CO2Et
O
0.7693
0.9952
0.8871
1.0172
1.0
0.95
2.0424
1.855
0.9601
0.9827
1.7729
2.0317
0.9803
3.1936
δ / ppm
9
8
7
6
5
4
3
2
1
0

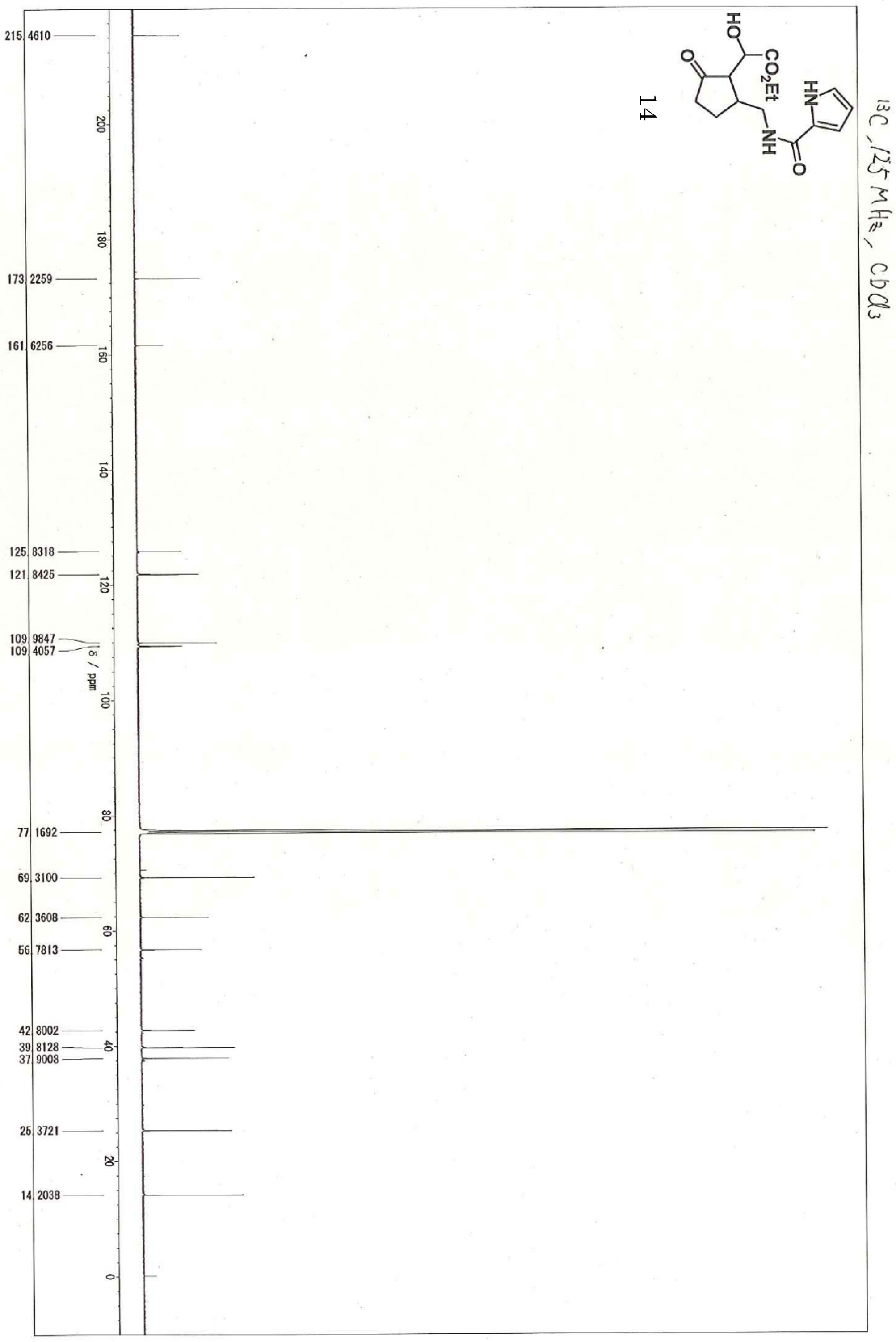

13C, 125 MHz, CDCl3
14
215.4610
173.2259
161.6256
125.8318
121.8425
109.9847
109.4057
77.1692
69.3100
62.3608
56.7813
42.8002
39.8128
37.9008
25.3721
14.2038
δ / ppm

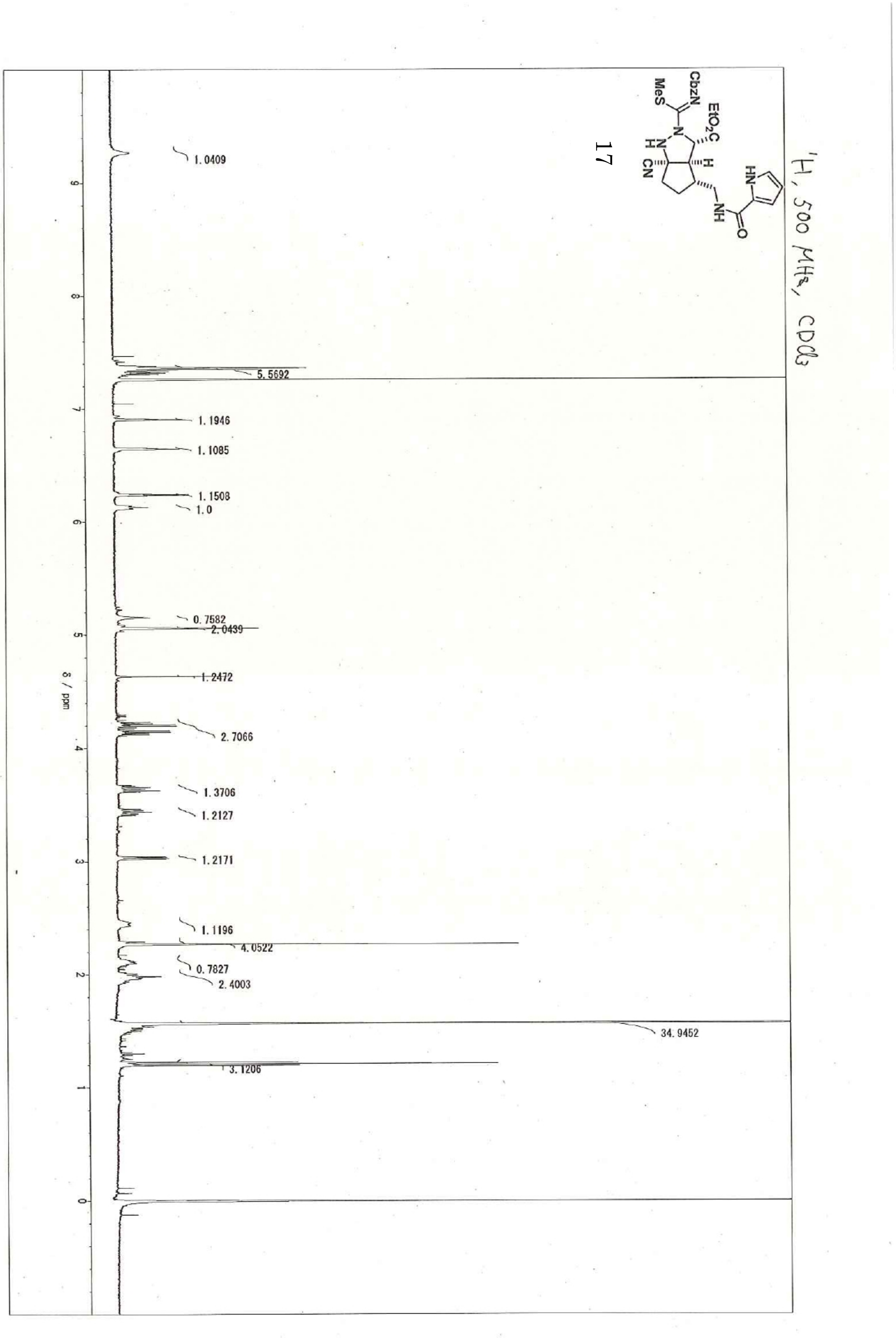

¹H, 500 MHz, CDCl3
17
EtO2C
CbzN
MeS
HN
NH
CN
δ / ppm
1.0409
5.5692
1.1946
1.1085
1.1508
1.0
0.7582
2.0439
1.2472
2.7066
1.3706
1.2127
1.2171
1.1196
4.0522
0.7827
2.4003
34.9452
3.1206

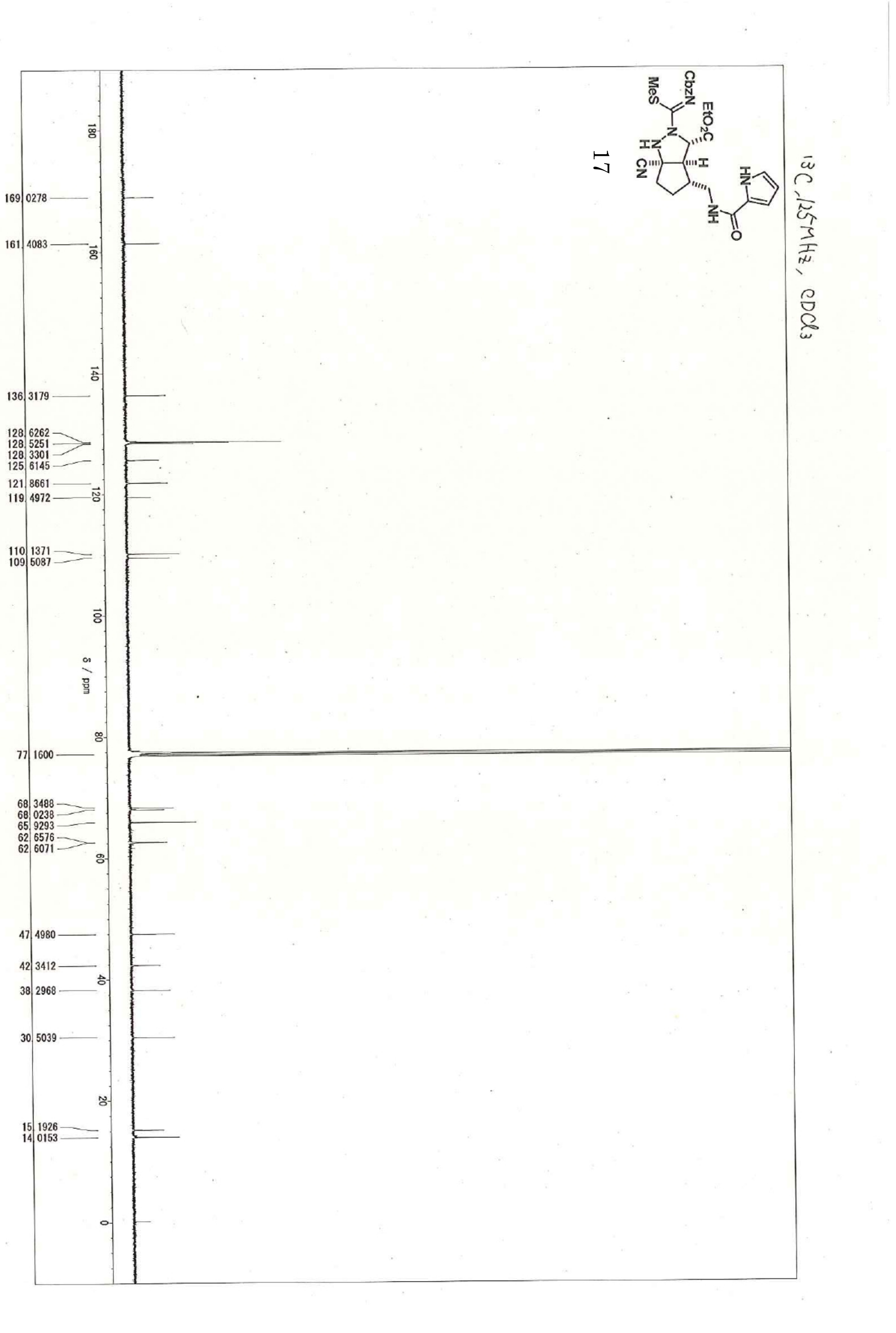

13C, 125MHz, CDCl3
17
EtO2C
CbzN
MeS
CN
NH
HN
O
169.0278
161.4083
136.3179
128.6262
128.5251
128.3301
125.6145
121.8661
119.4972
110.1371
109.5087
77.1600
68.3488
68.0238
65.9293
62.6576
62.6071
47.4980
42.3412
38.2968
30.5039
15.1926
14.0153
180
160
140
120
100
δ / ppm
80
60
40
20
0

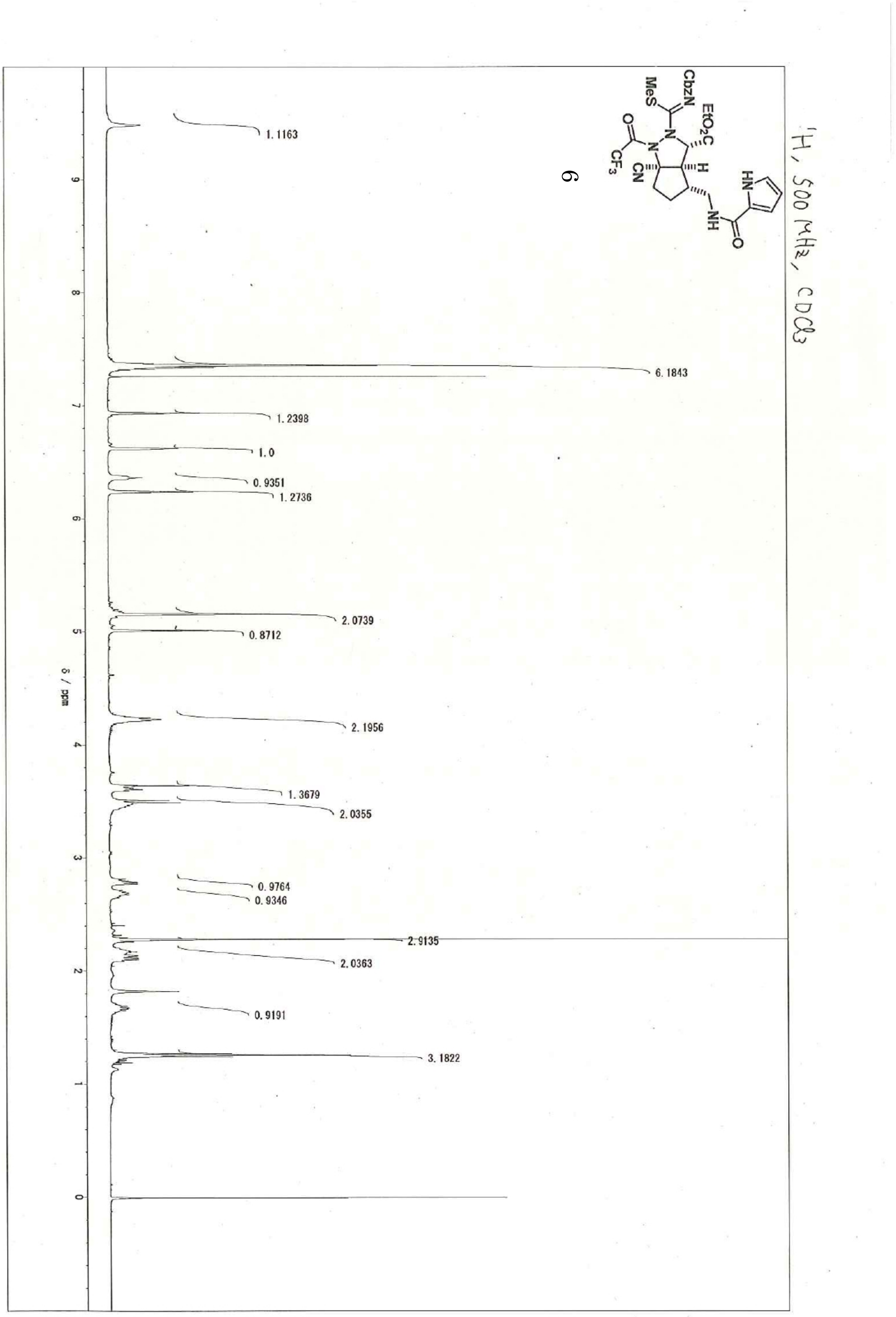

1H, 500 MHz, CDCl3
6
1.1163
6.1843
1.2398
1.0
0.9351
1.2736
2.0739
0.8712
2.1956
1.3679
2.0355
0.9764
0.9346
2.9135
2.0363
0.9191
3.1822
δ / ppm

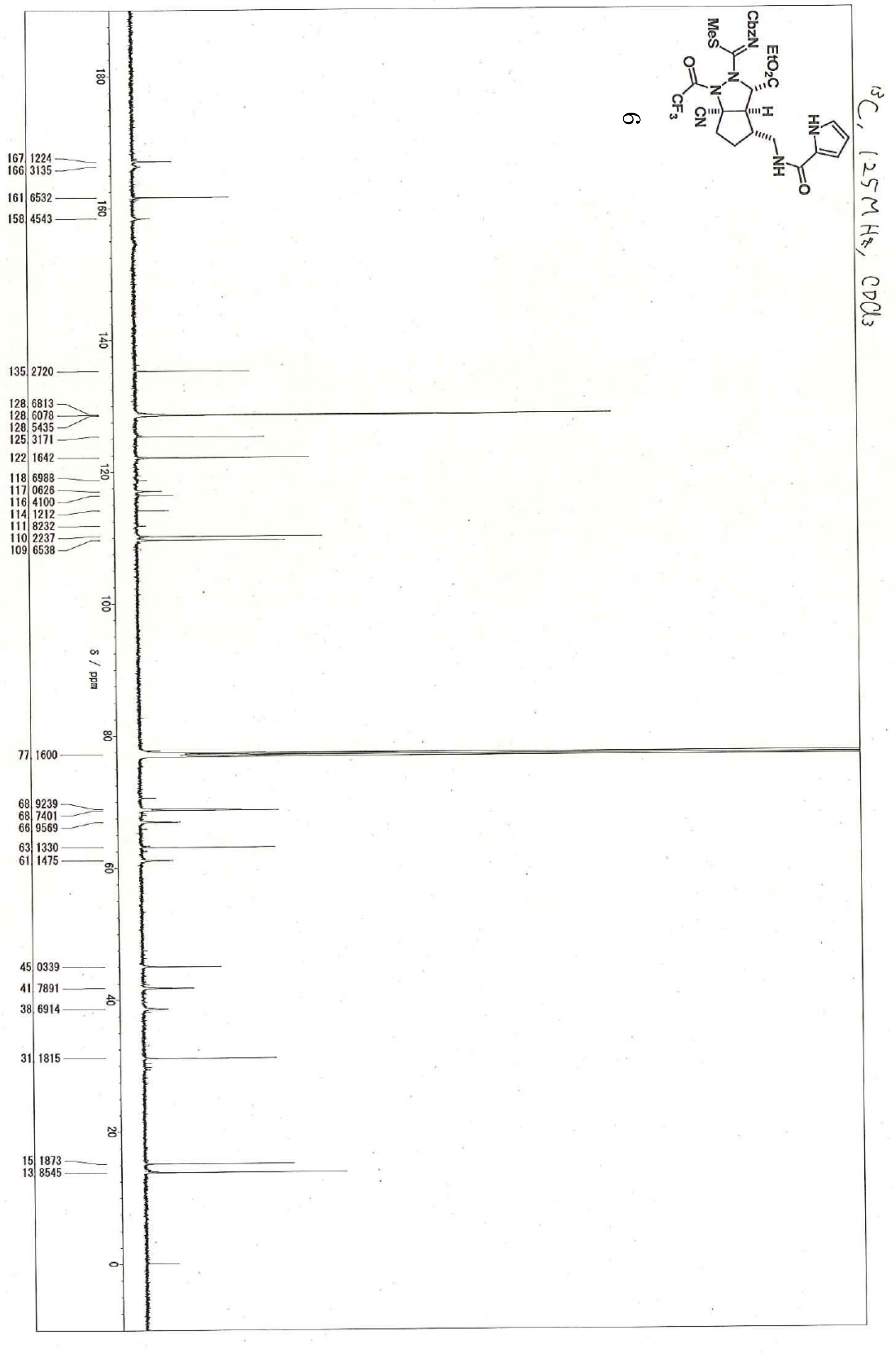
13C, 125 MHz, CDCl3
6
EtO2C
CbzN
MeS
N
H
HN
NH
O
CN
CF3
167.1224
166.3135
161.6532
158.4543
135.2720
128.6813
128.6078
128.5435
125.3171
122.1642
118.6988
117.0626
116.4100
114.1212
111.8232
110.2237
109.6538
77.1600
68.9239
68.7401
66.9569
63.1330
61.1475
45.0339
41.7891
38.6914
31.1815
15.1873
13.8545
δ / ppm

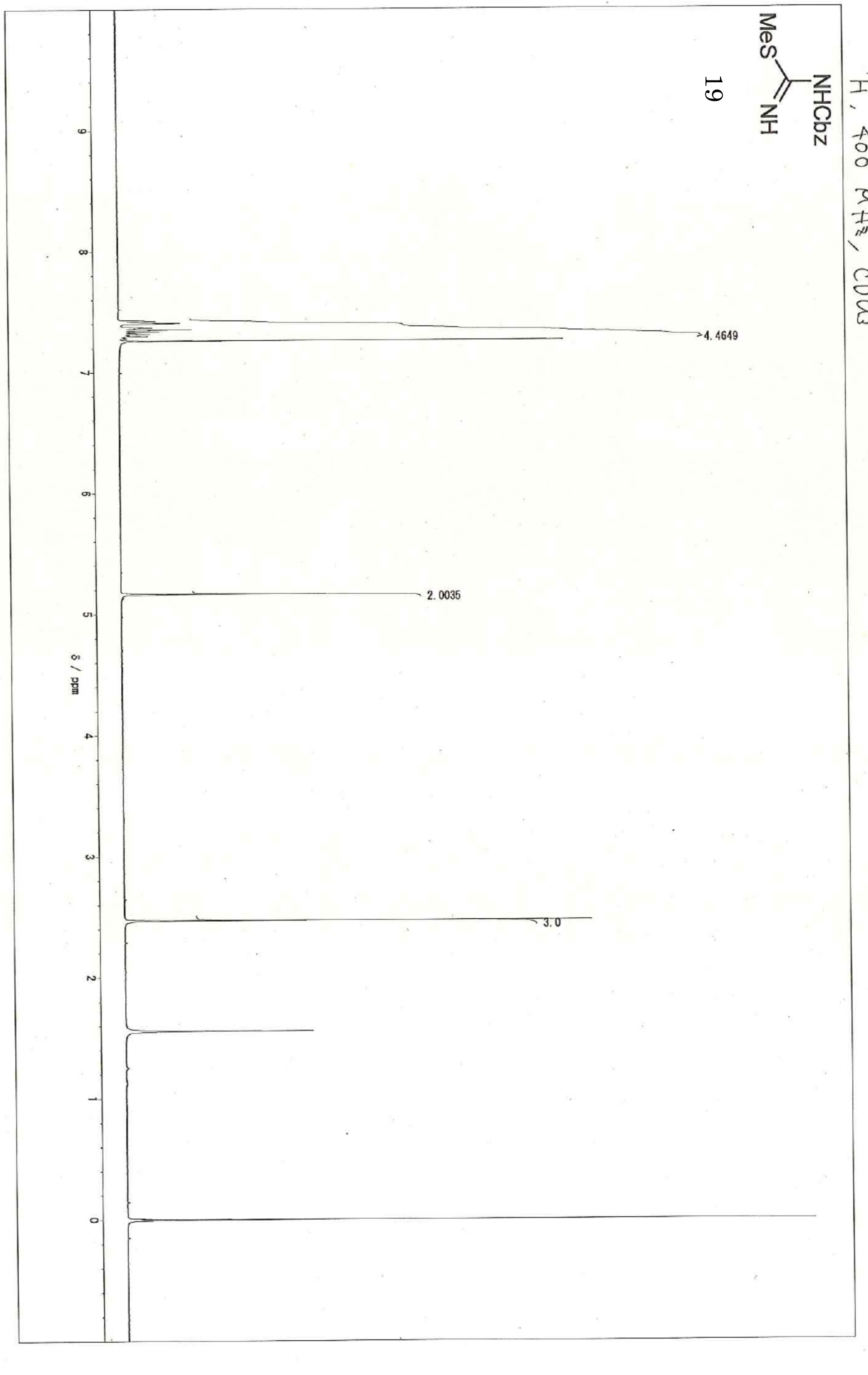

1H, 400 MHz, CDCl3
NHCbz
MeS
NH
19
4.4649
2.0035
3.0
δ / ppm
9
8
7
6
5
4
3
2
1
0

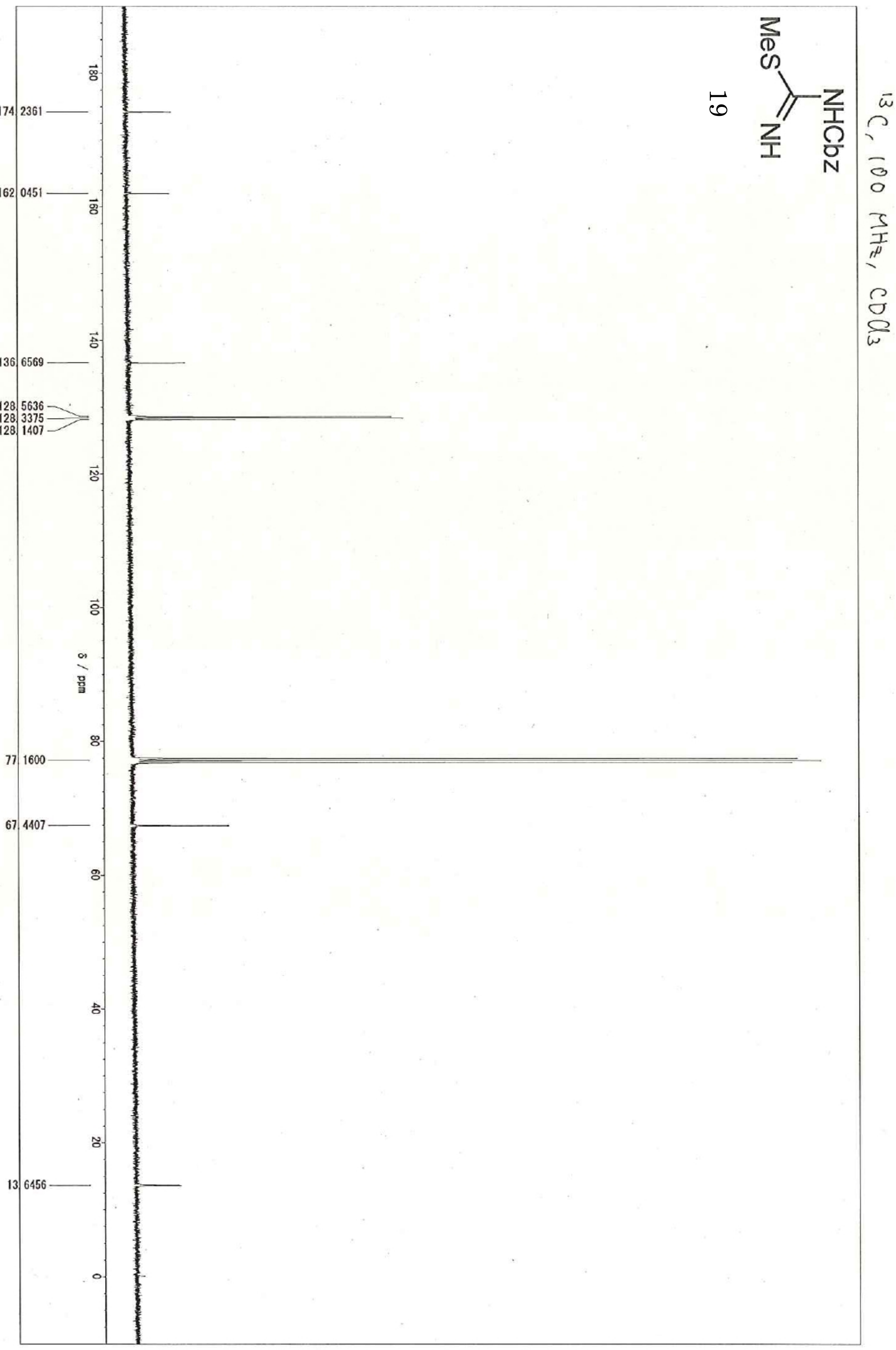
13C, 100 MHz, CDCl3
NHCbz
MeS
NH
19
174.2361
162.0451
136.6569
128.5636
128.3375
128.1407
77.1600
67.4407
13.6456
δ / ppm
180
160
140
120
100
80
60
40
20
0

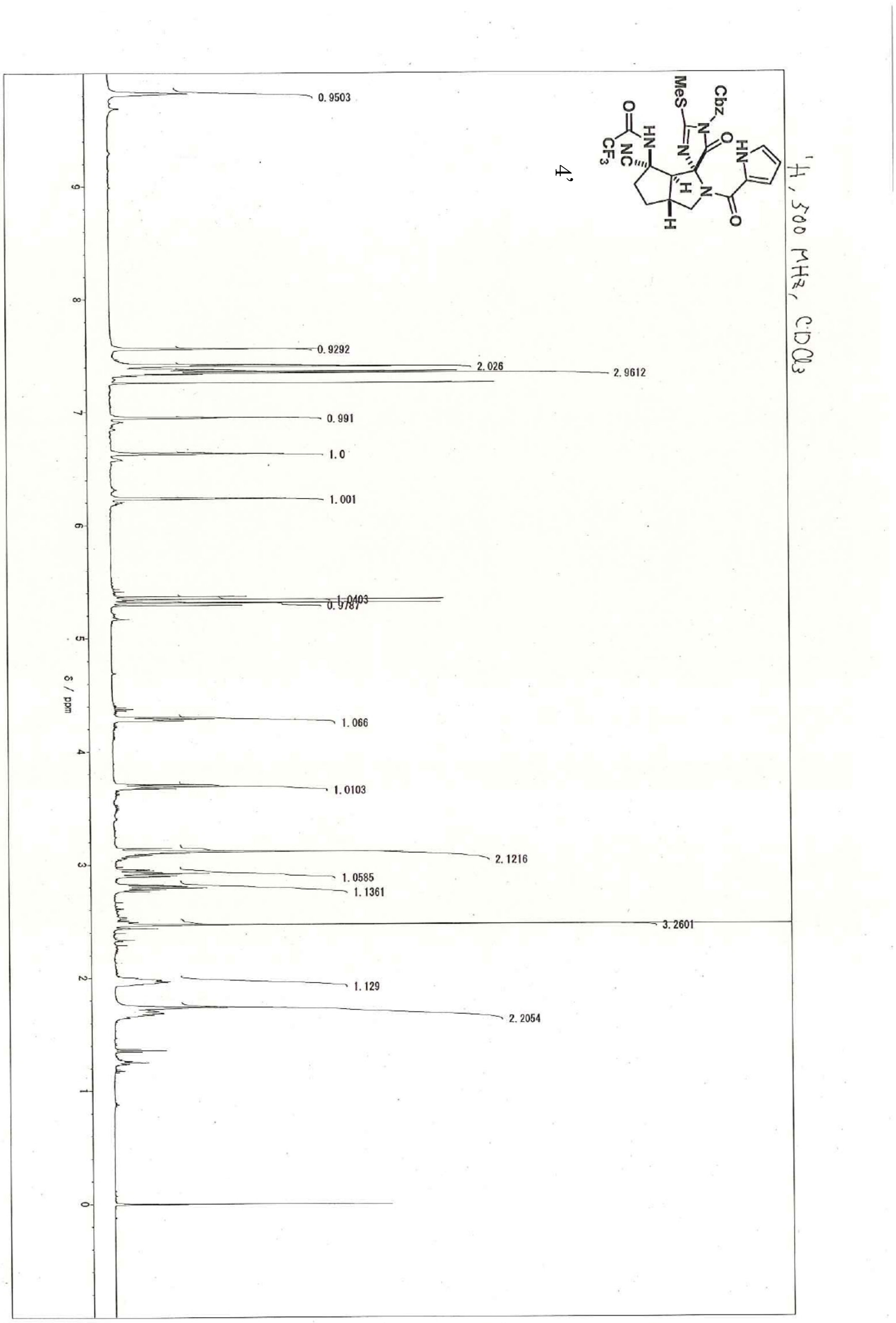

1H, 500 MHz, CDCl3
4'
Cbz
MeS
HN
NC
CF3
H
δ / ppm
0.9503
0.9292
2.026
2.9612
0.991
1.0
1.001
1.0403
0.9787
1.066
1.0103
2.1216
1.0585
1.1361
3.2601
1.129
2.2054

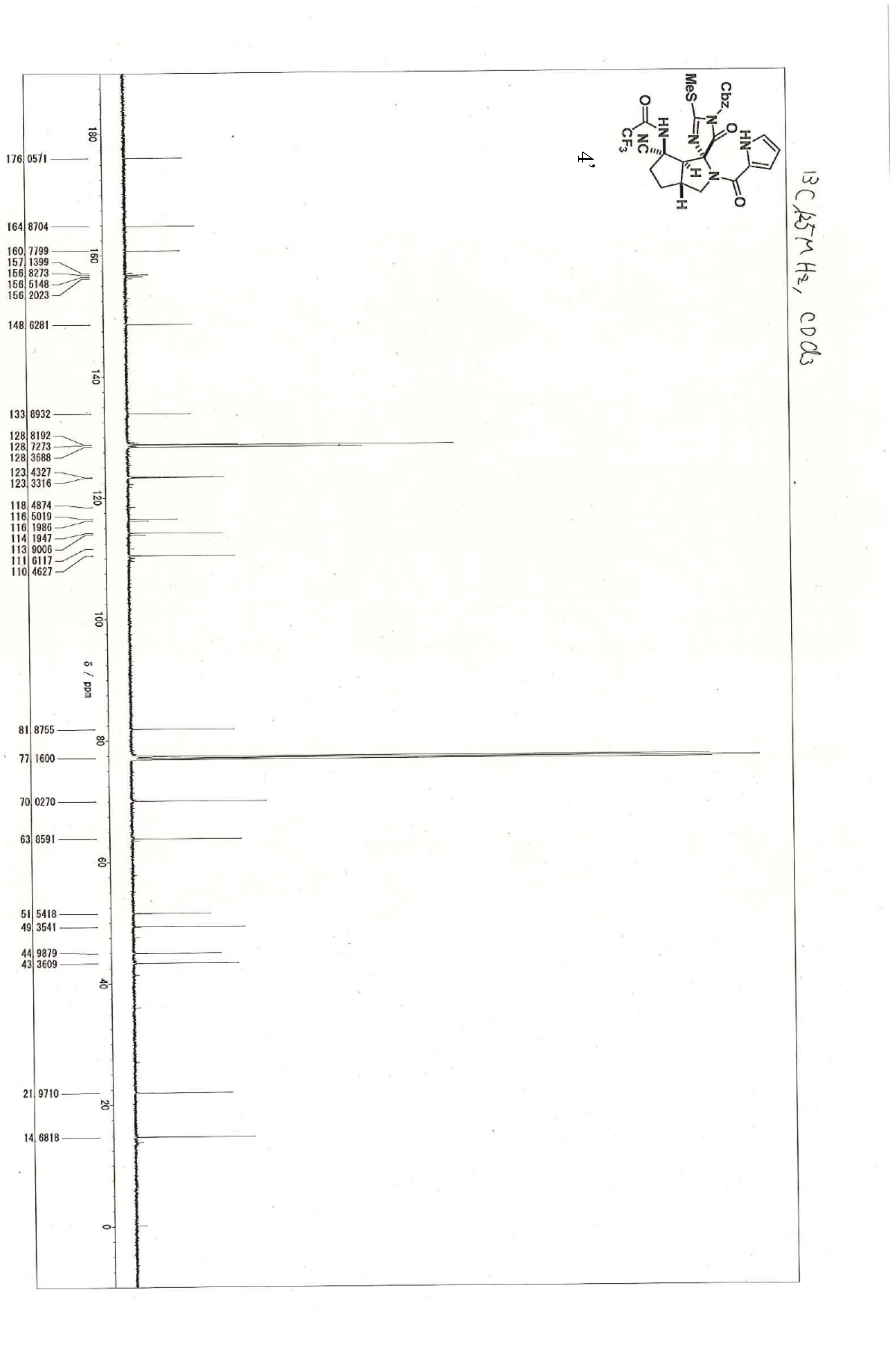
13C 125MHz, CDCl3
4'
Cbz
MeS
HN
NC
CF3
176.0571
164.8704
160.7799
157.1399
156.8273
156.5148
156.2023
148.6281
133.8932
128.8192
128.7273
128.3688
123.4327
123.3316
118.4874
116.5019
116.1986
114.1947
113.9006
111.6117
110.4627
81.8755
77.1600
70.0270
63.8591
51.5418
49.3541
44.9879
43.3609
21.9710
14.6818
δ / ppm

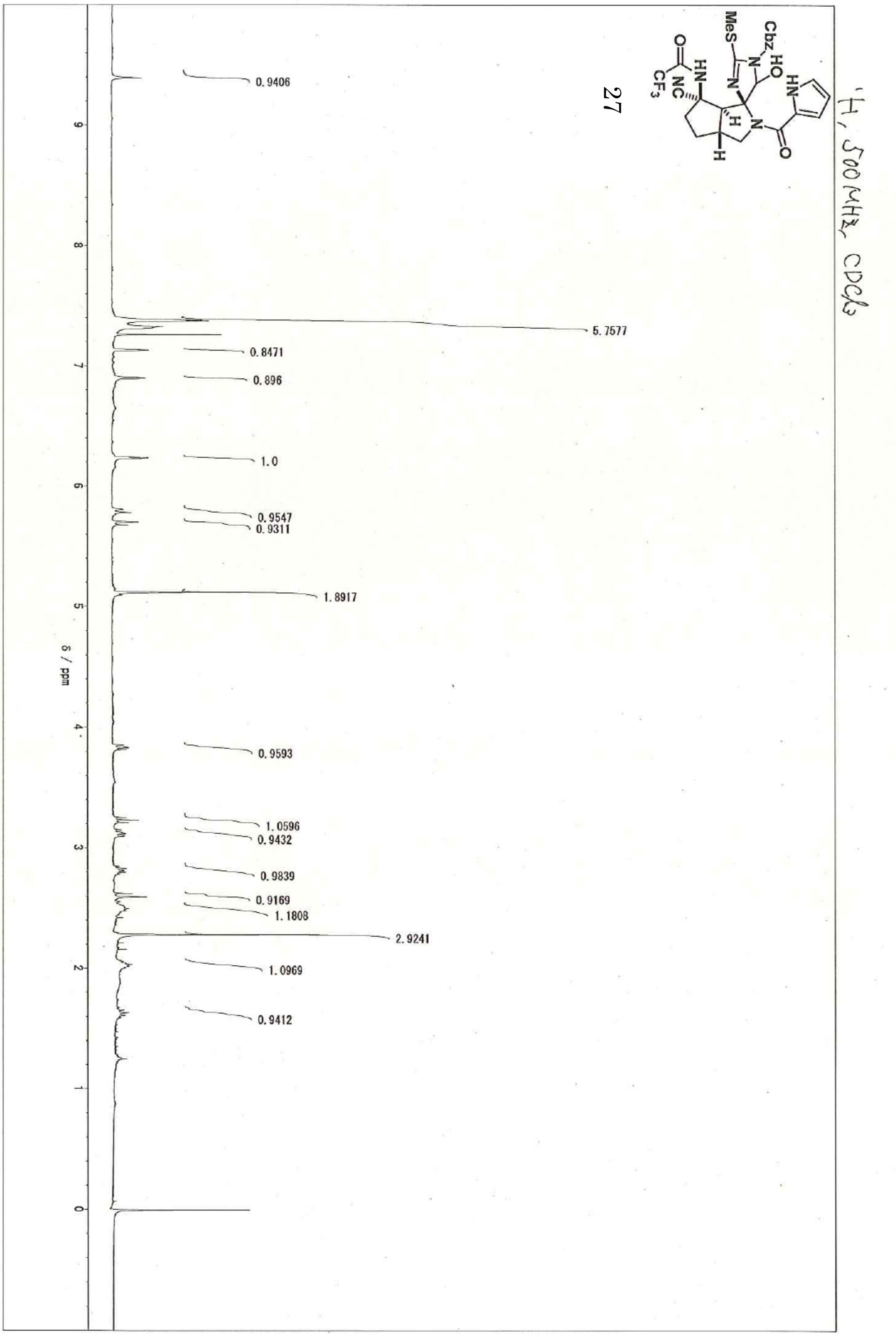
1H, 500MHz CDCl3
27
HN
O
N
HO
Cbz
N
N
MeS
H
H
HN
O
NC
CF3
0.9406
5.7577
0.8471
0.896
1.0
0.9547
0.9311
1.8917
0.9593
1.0596
0.9432
0.9839
0.9169
1.1808
2.9241
1.0969
0.9412
9
8
7
6
5
4
3
2
1
0
δ / ppm

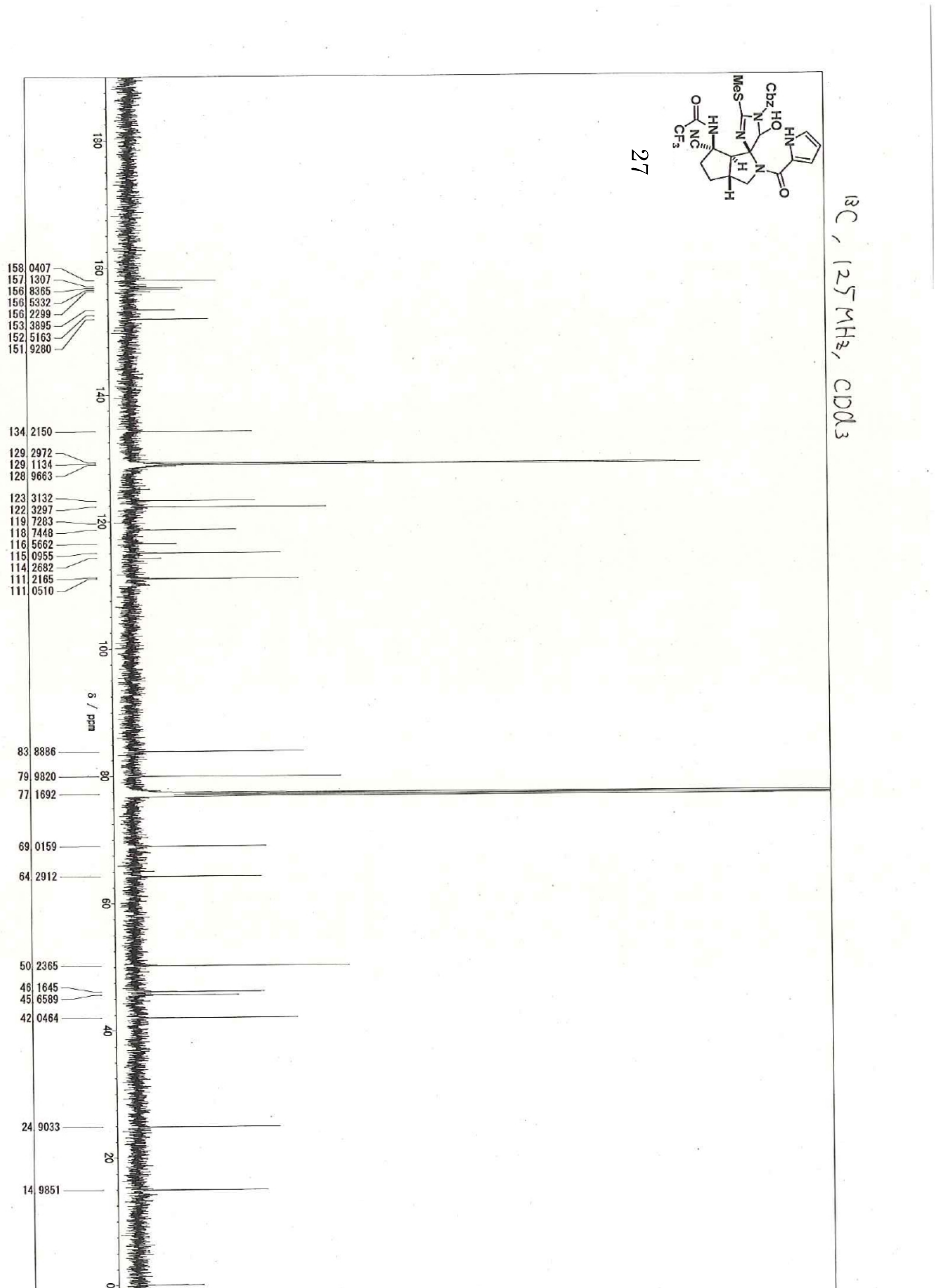
13C, 125 MHz, CDCl3
27
158.0407
157.1307
156.8365
156.5332
156.2299
153.3895
152.5163
151.9280
134.2150
129.2972
129.1134
128.9663
123.3132
122.3297
119.7283
118.7448
116.5662
115.0955
114.2682
111.2165
111.0510
83.8886
79.9820
77.1692
69.0159
64.2912
50.2365
46.1645
45.6589
42.0464
24.9033
14.9851
δ / ppm

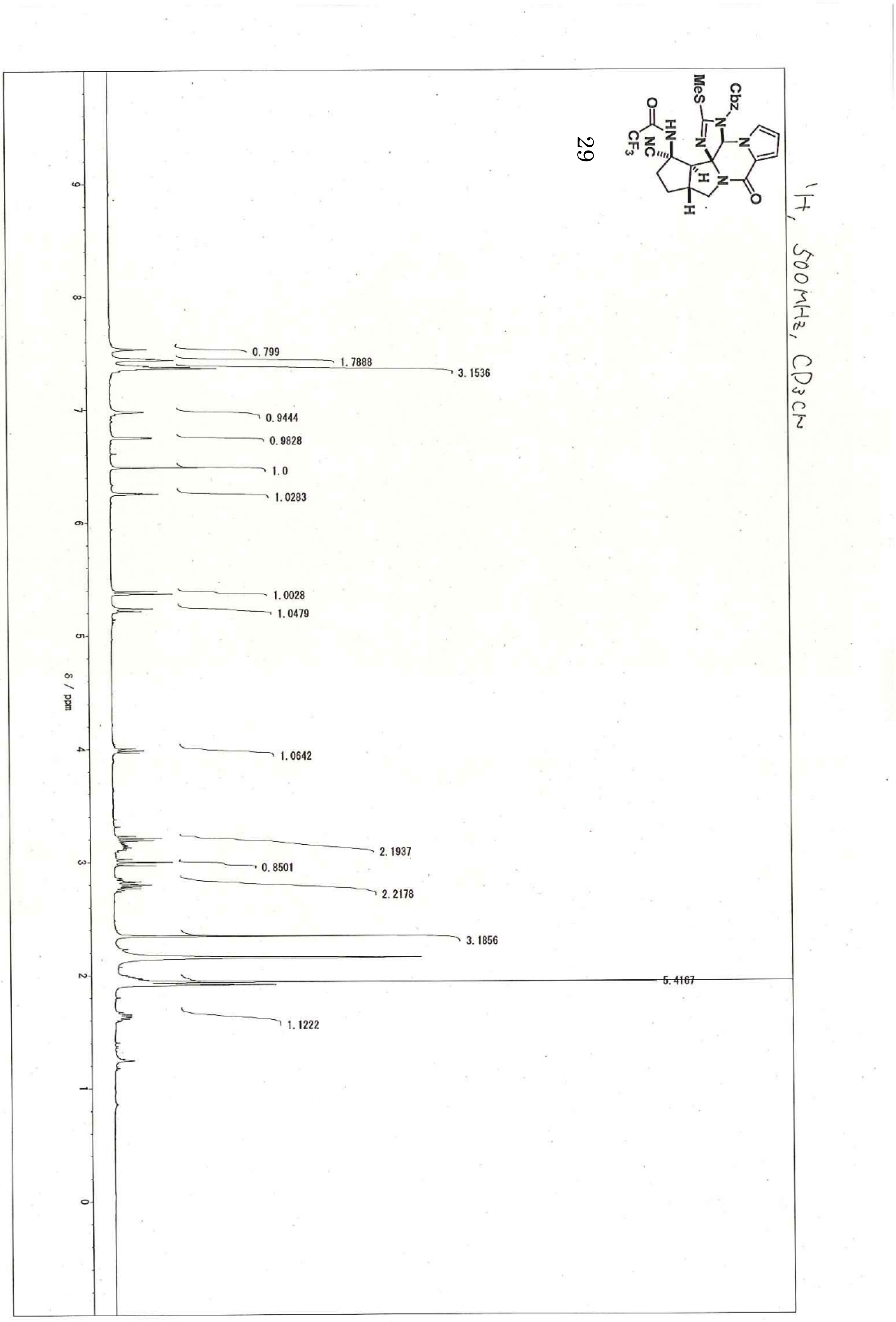

29
1H, 500MHz, CD3CN
δ / ppm
0.799
1.7888
3.1536
0.9444
0.9828
1.0
1.0283
1.0028
1.0479
1.0642
2.1937
0.8501
2.2178
3.1856
5.4167
1.1222

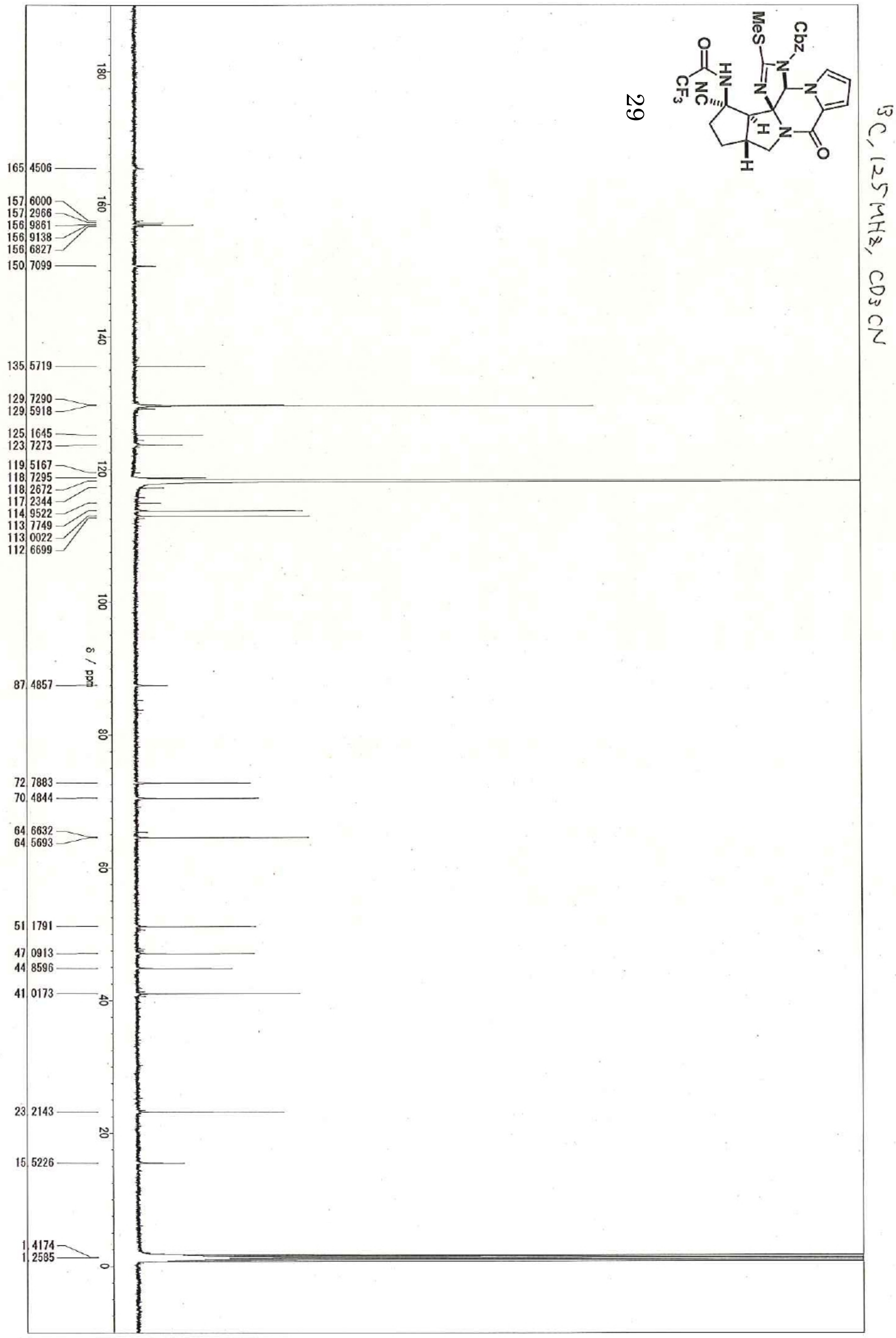

13C, 125 MHz, CD3CN
29
Cbz
MeS
HN
NC
CF3
H
O
165.4506
157.6000
157.2966
156.9861
156.9138
156.6827
150.7099
135.5719
129.7290
129.5918
125.1645
123.7273
119.5167
118.7295
118.2672
117.2344
114.9522
113.7749
113.0022
112.6699
87.4857
72.7883
70.4844
64.6632
64.5693
51.1791
47.0913
44.8596
41.0173
23.2143
15.5226
1.4174
1.2585
δ / ppm
180
160
140
120
100
80
60
40
20
0

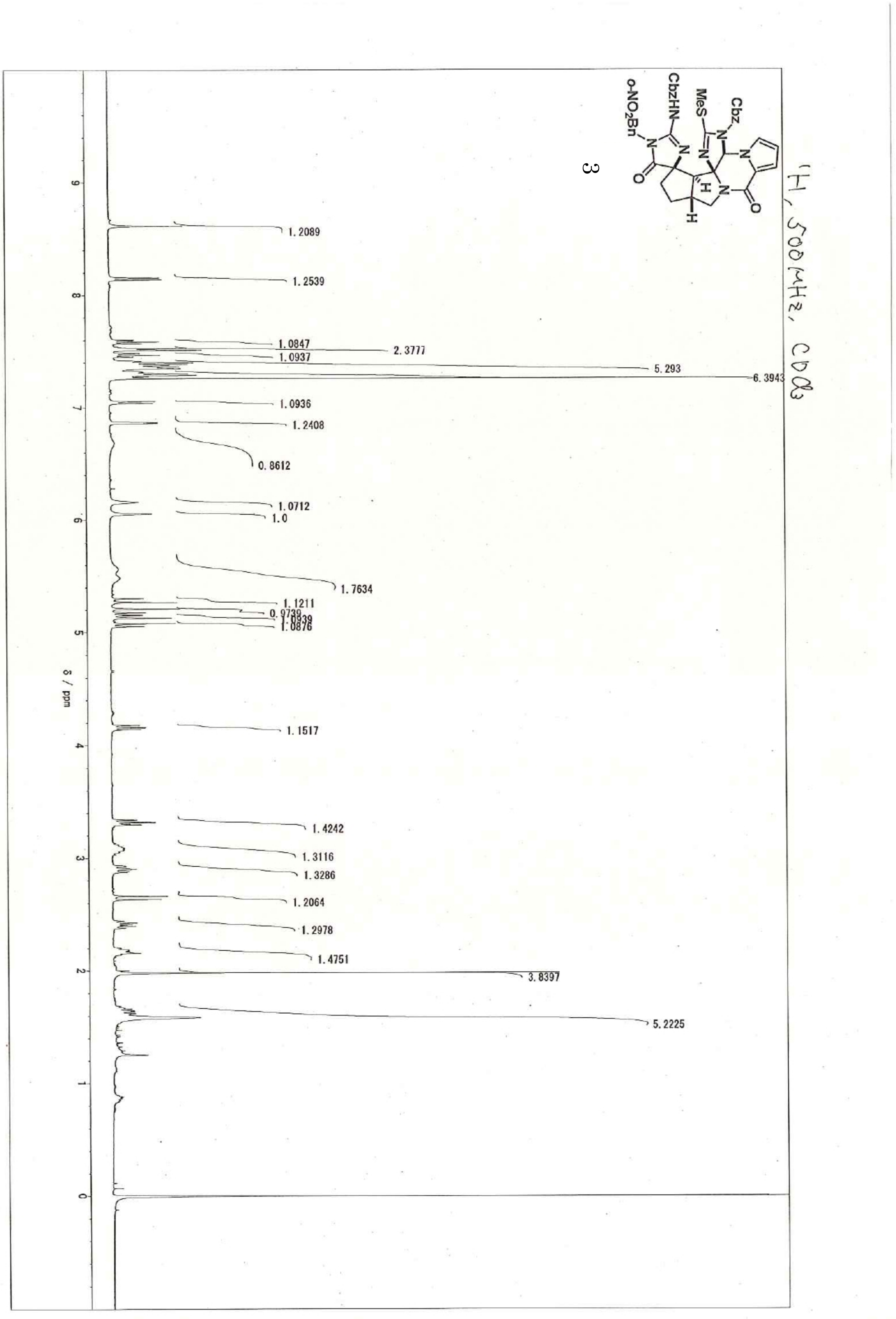
1H, 500MHz, CDCl3
3
Cbz
MeS
CbzHN
o-NO2Bn
δ / ppm

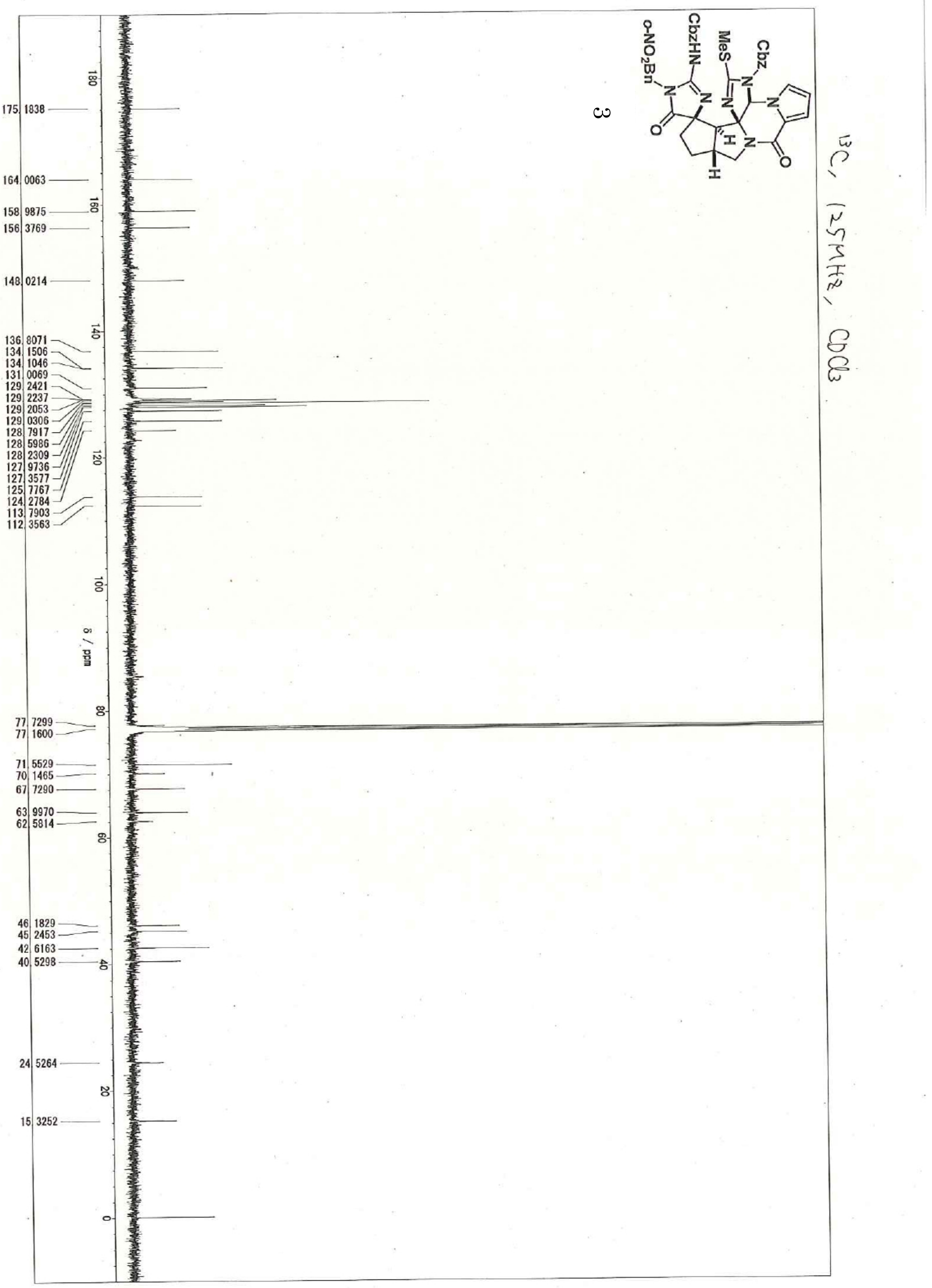
13C, 125MHz, CDCl3
3
CbzHN
MeS
Cbz
o-NO2Bn
175.1838
164.0063
158.9875
156.3769
148.0214
136.8071
134.1506
134.1046
131.0069
129.2421
129.2237
129.2053
129.0306
128.7917
128.5986
128.2309
127.9736
127.3577
125.7767
124.2784
113.7903
112.3563
77.7299
77.1600
71.5529
70.1465
67.7290
63.9970
62.5814
46.1829
45.2453
42.6163
40.5298
24.5264
15.3252
δ / ppm

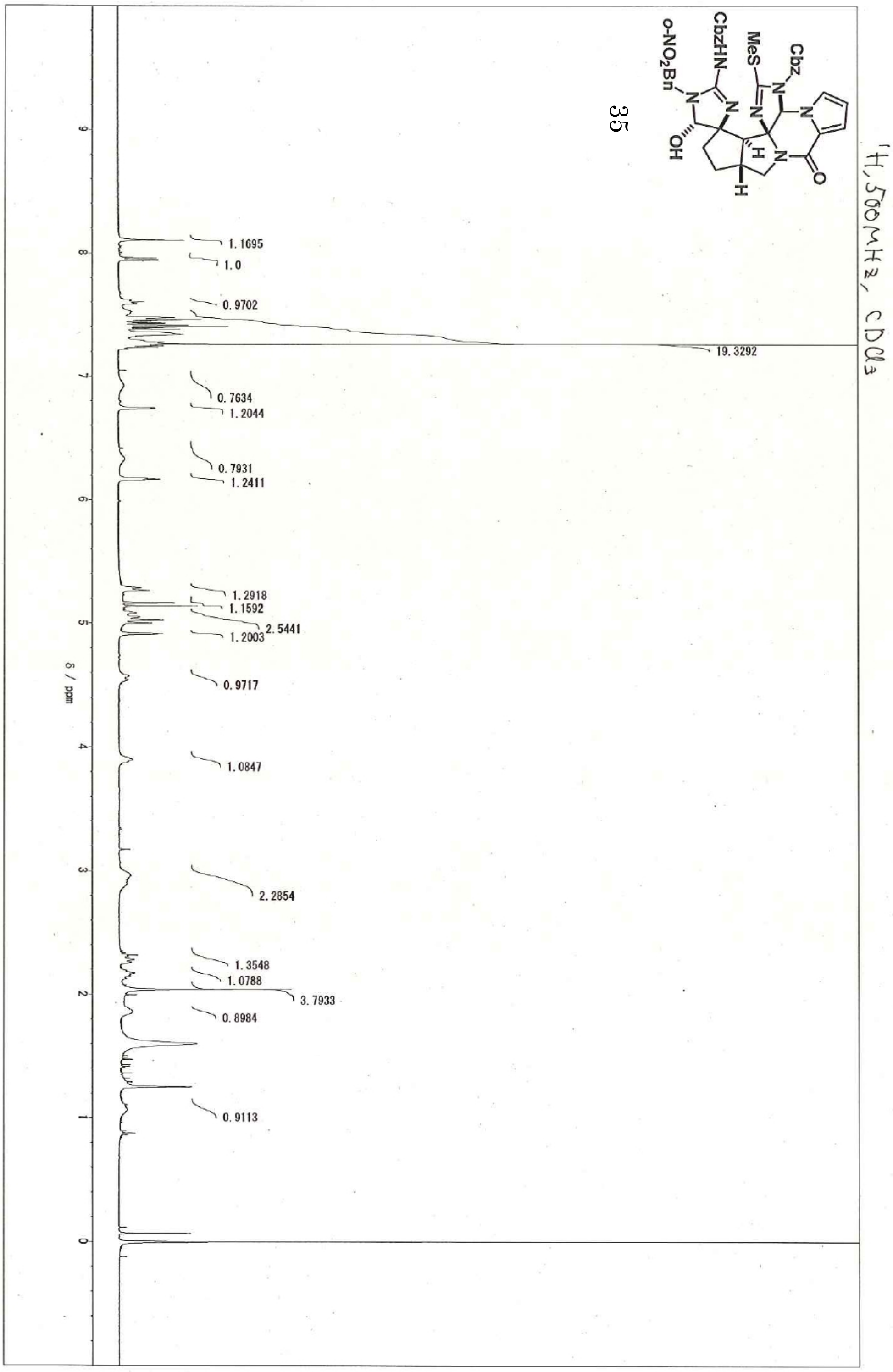

35
1H, 500MHz, CDCl3
1.1695
1.0
0.9702
19.3292
0.7634
1.2044
0.7931
1.2411
1.2918
1.1592
2.5441
1.2003
0.9717
1.0847
2.2854
1.3548
1.0788
3.7933
0.8984
0.9113
δ / ppm

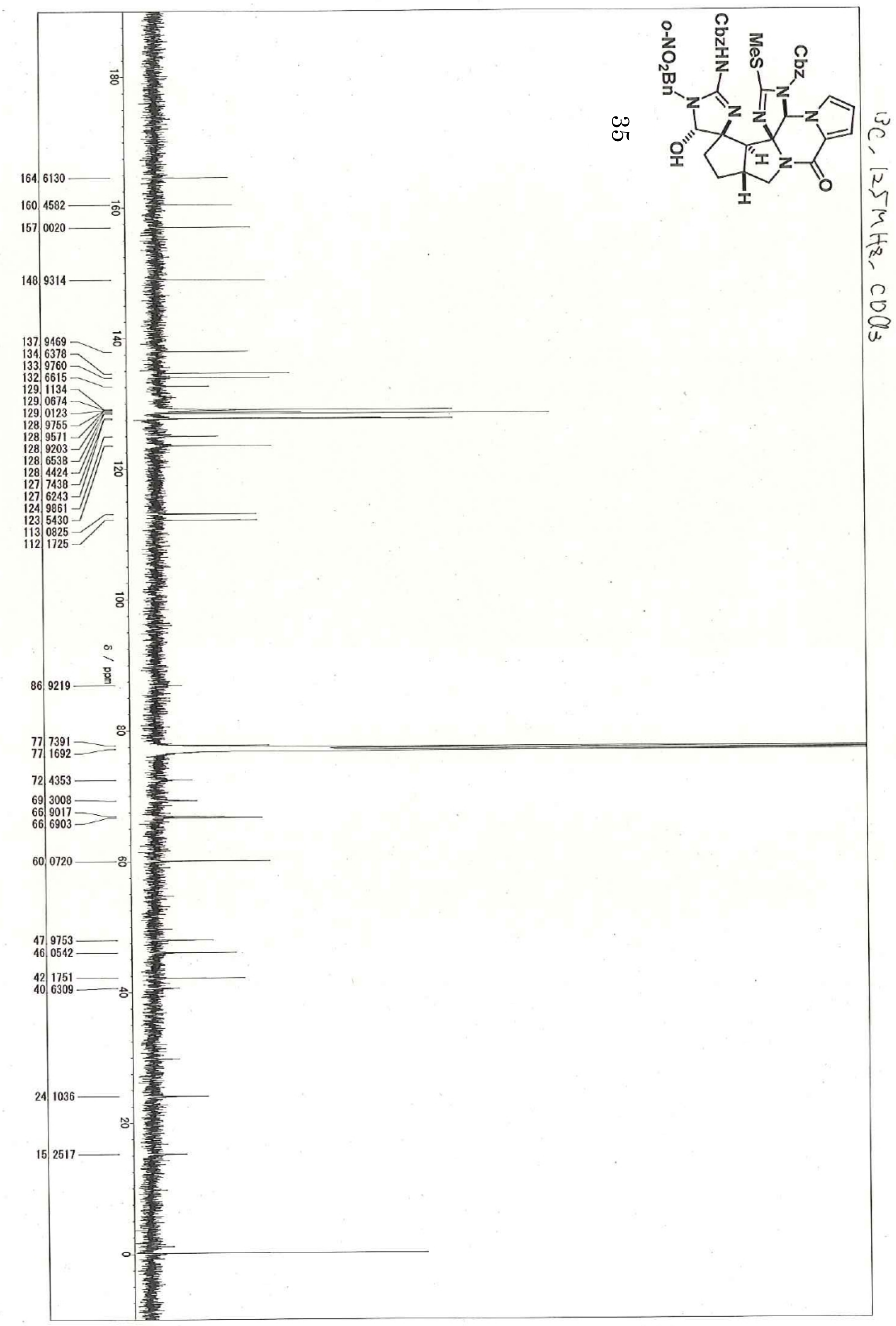
13C, 125 MHz, CDCl3
Cbz
MeS
CbzHN
o-NO2Bn
OH
H
35
164.6130
160.4582
157.0020
148.9314
137.9469
134.6378
133.9760
132.6615
129.1134
129.0674
129.0123
128.9755
128.9571
128.9203
128.6538
128.4424
127.7438
127.6243
124.9861
123.5430
113.0825
112.1725
86.9219
77.7391
77.1692
72.4353
69.3008
66.9017
66.6903
60.0720
47.9753
46.0542
42.1751
40.6309
24.1036
15.2517
δ / ppm

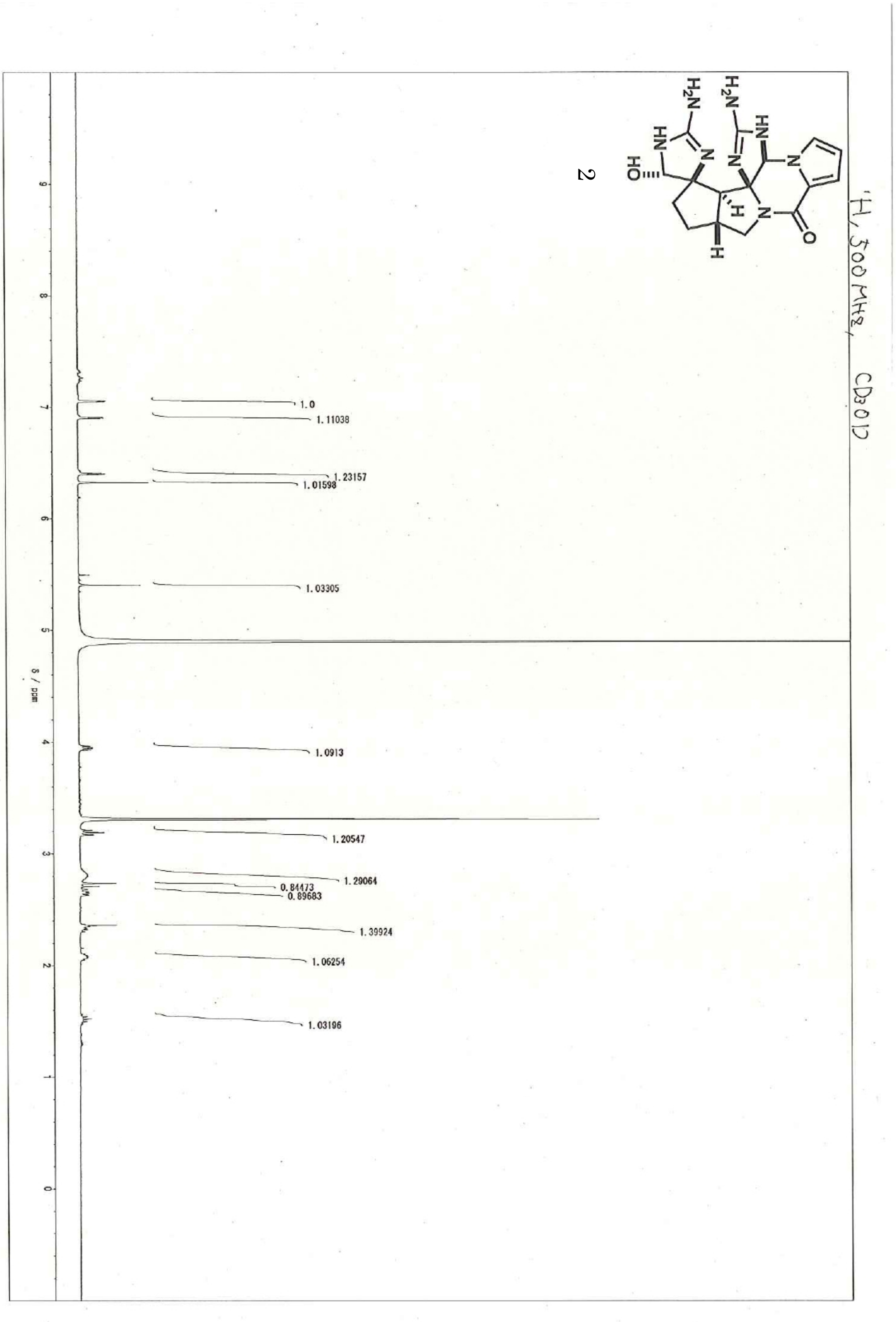
1H, 300 MHz, CD3OD
2
1.0
1.11038
1.23157
1.01598
1.03305
1.0913
1.20547
1.29064
0.84473
0.89683
1.39924
1.06254
1.03196
δ / ppm

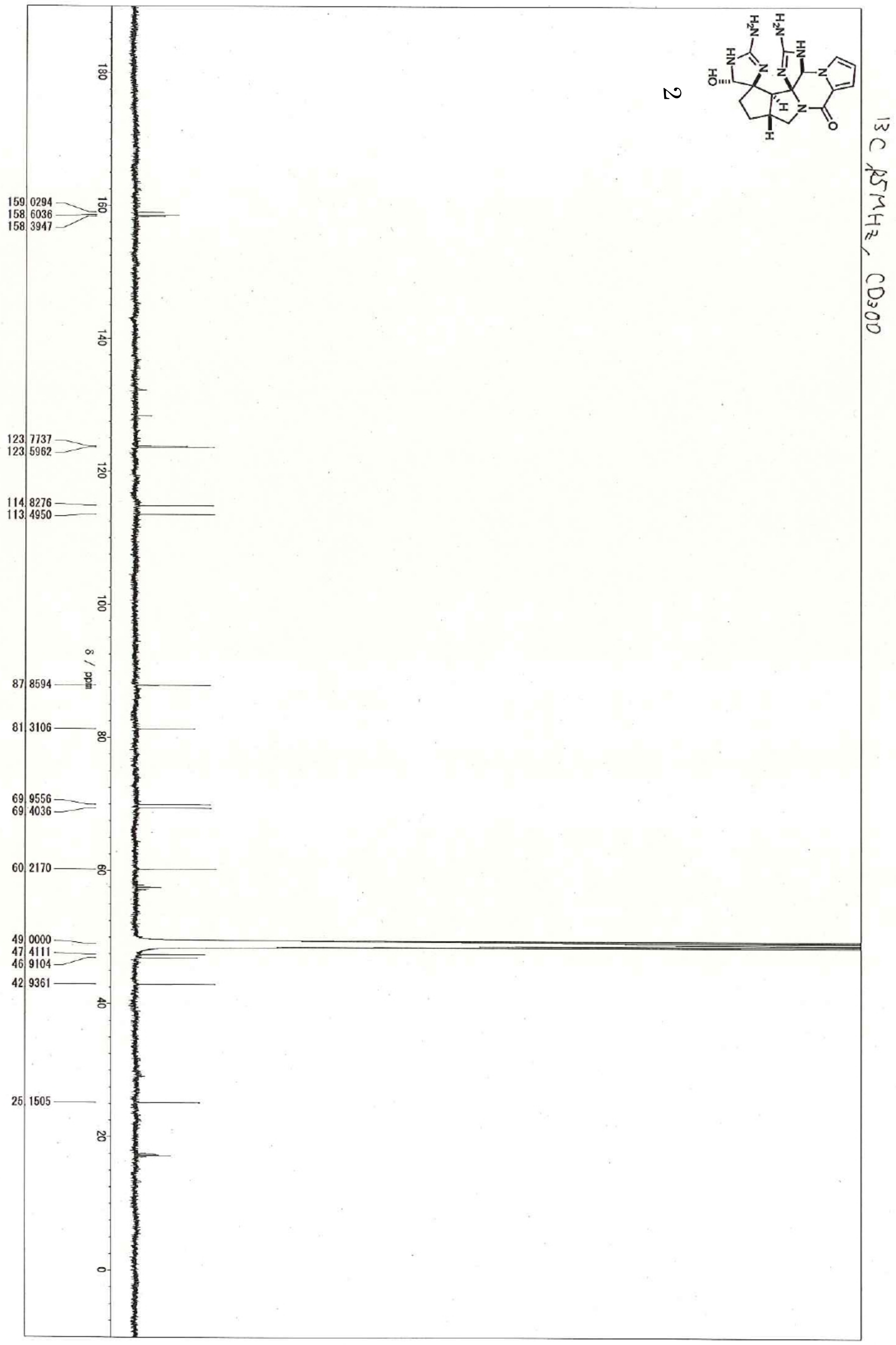
13C 125MHz, CD3OD
2
159.0294
158.6036
158.3947
123.7737
123.5962
114.8276
113.4950
87.8594
81.3106
69.9556
69.4036
60.2170
49.0000
47.4111
46.9104
42.9361
25.1505
δ / ppm

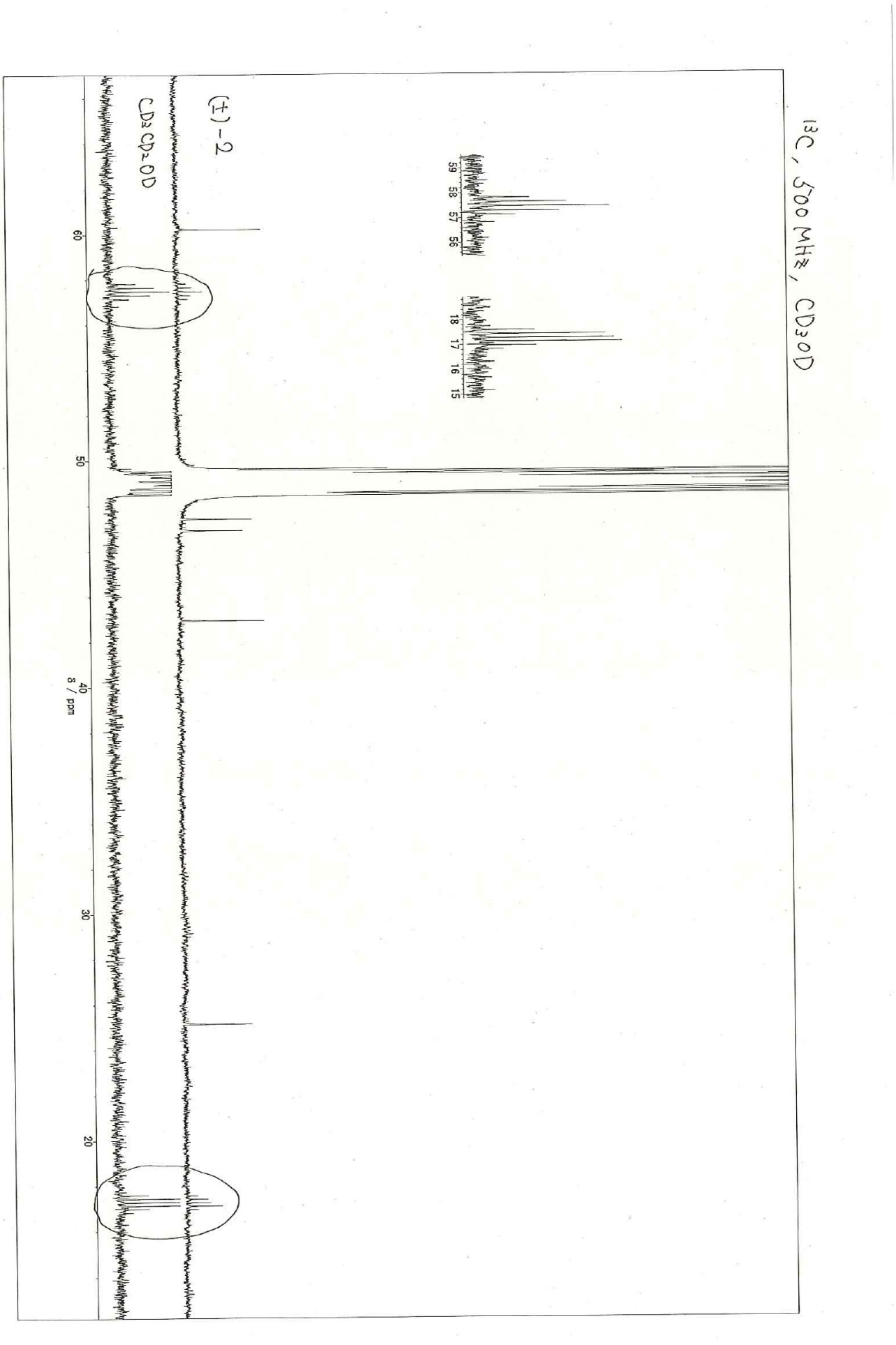
13C, 500 MHz, CD3OD
(±)-2
CD3CD2OD
59 58 57 56
18 17 16 15
60
50
40
δ / ppm
30
20

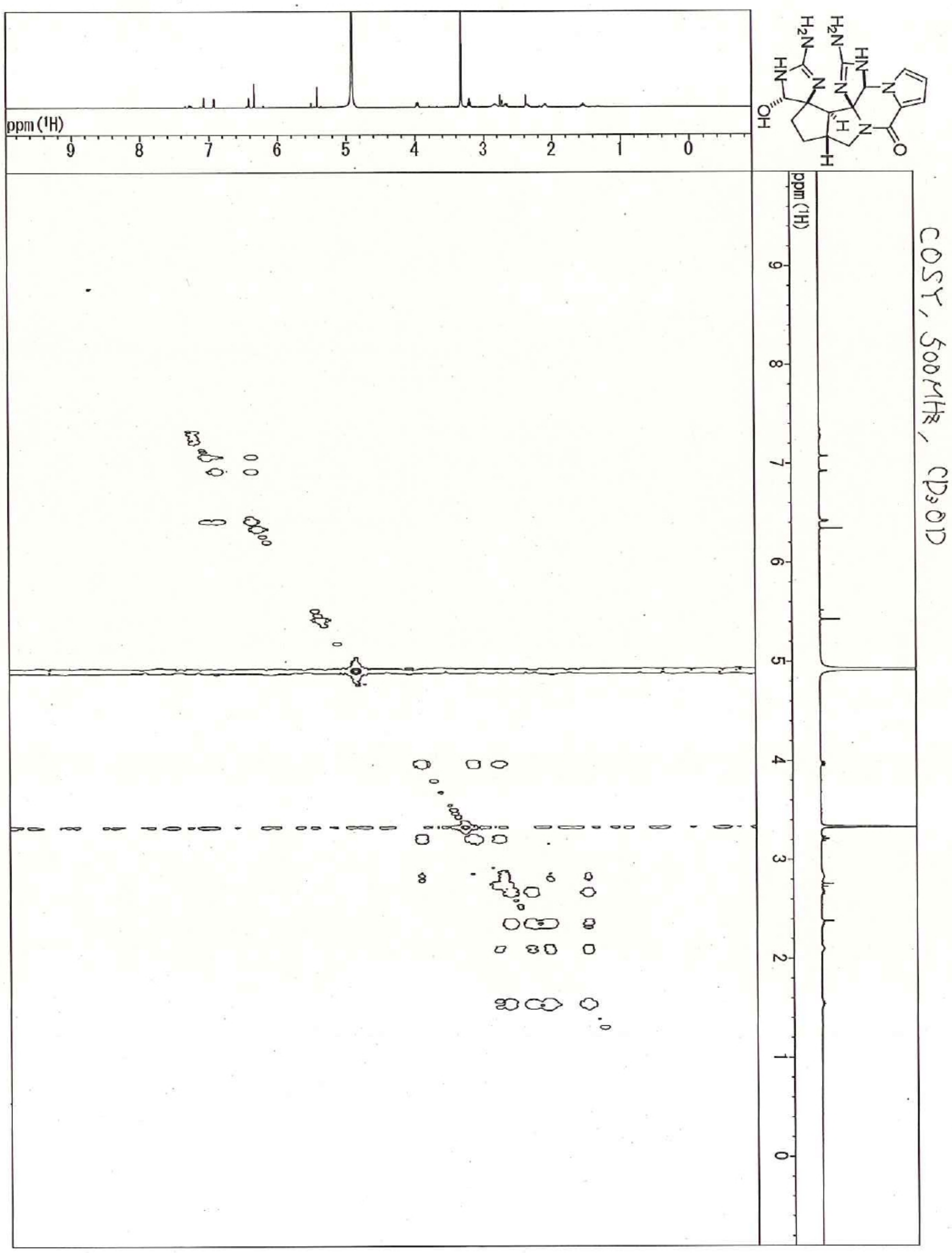
COSY, 500MHz, CD3OD
ppm (1H)
ppm (1H)

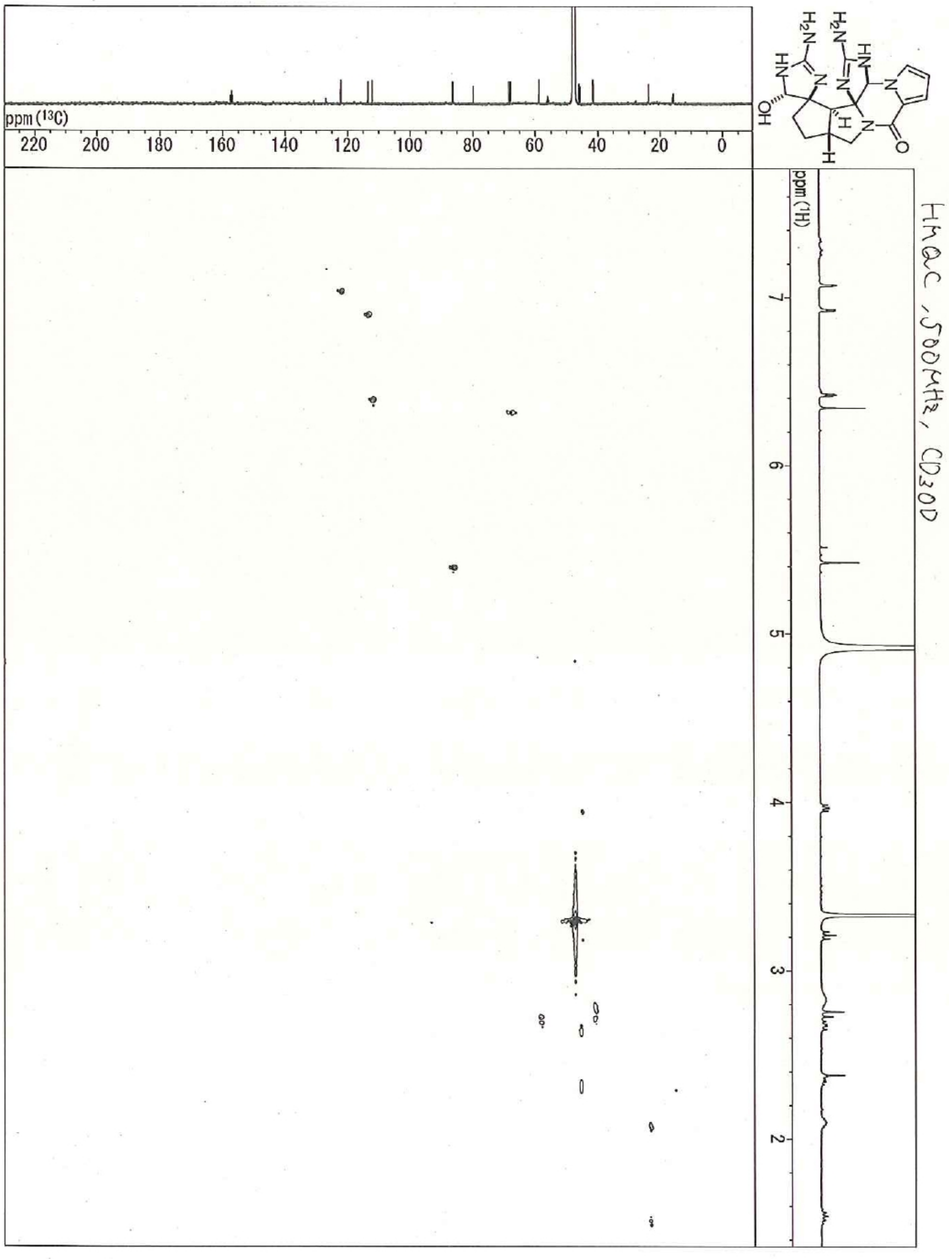
ppm (13C)
220 200 180 160 140 120 100 80 60 40 20 0
ppm (1H)
7 6 5 4 3 2
HMQC, 500MHz, CD3OD

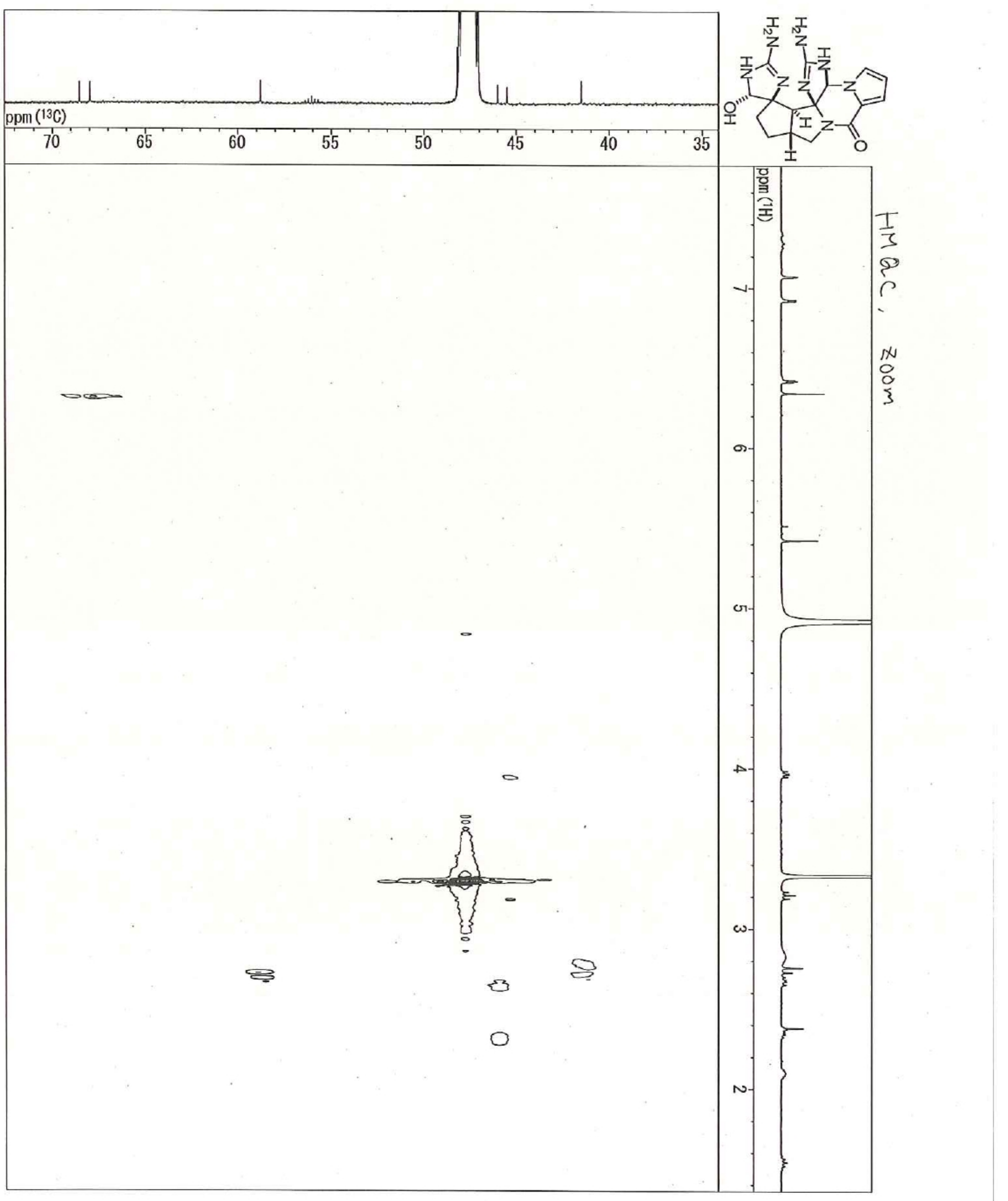
ppm (13C)
70
65
60
55
50
45
40
35
ppm (1H)
7
6
5
4
3
2
HMQC, zoom

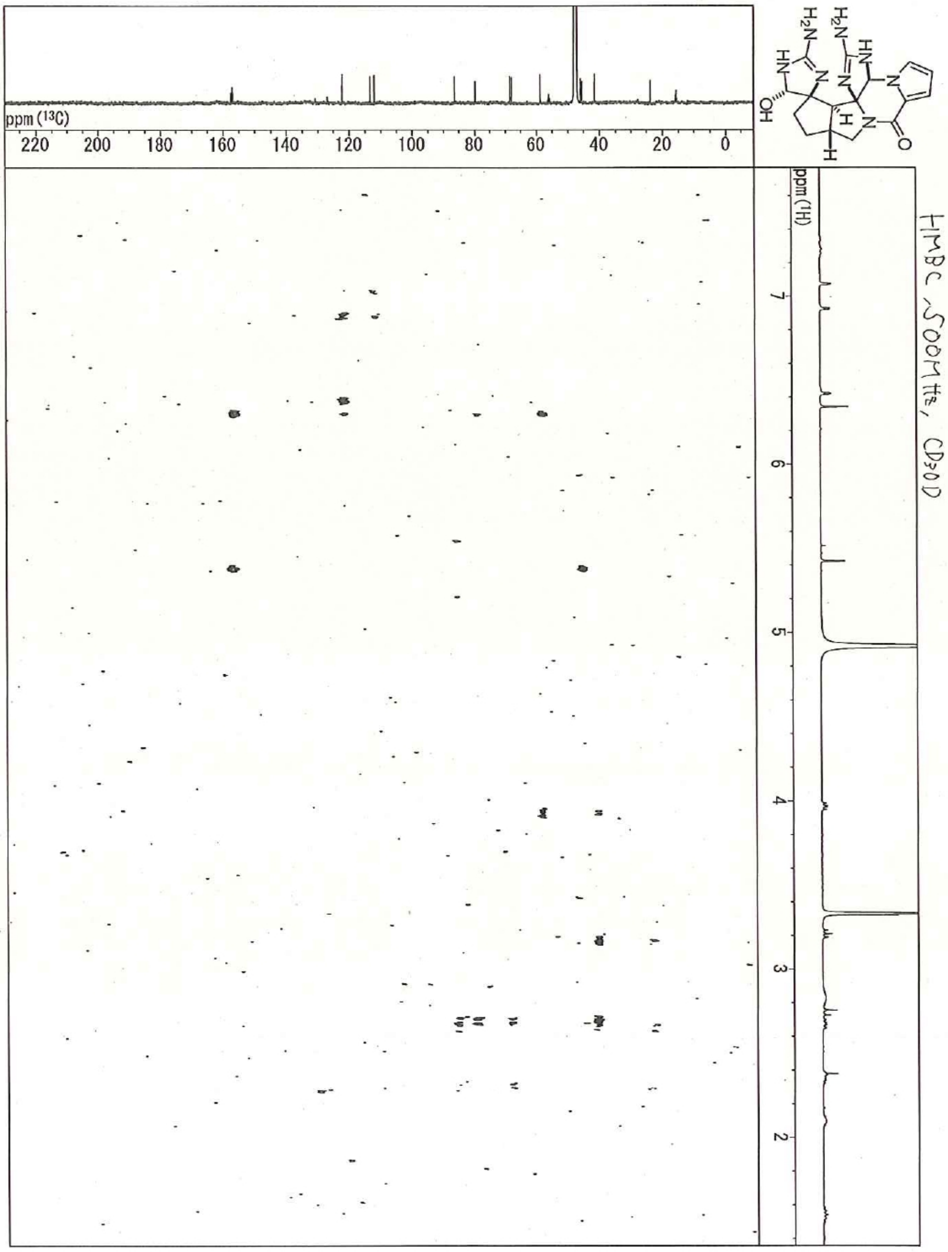

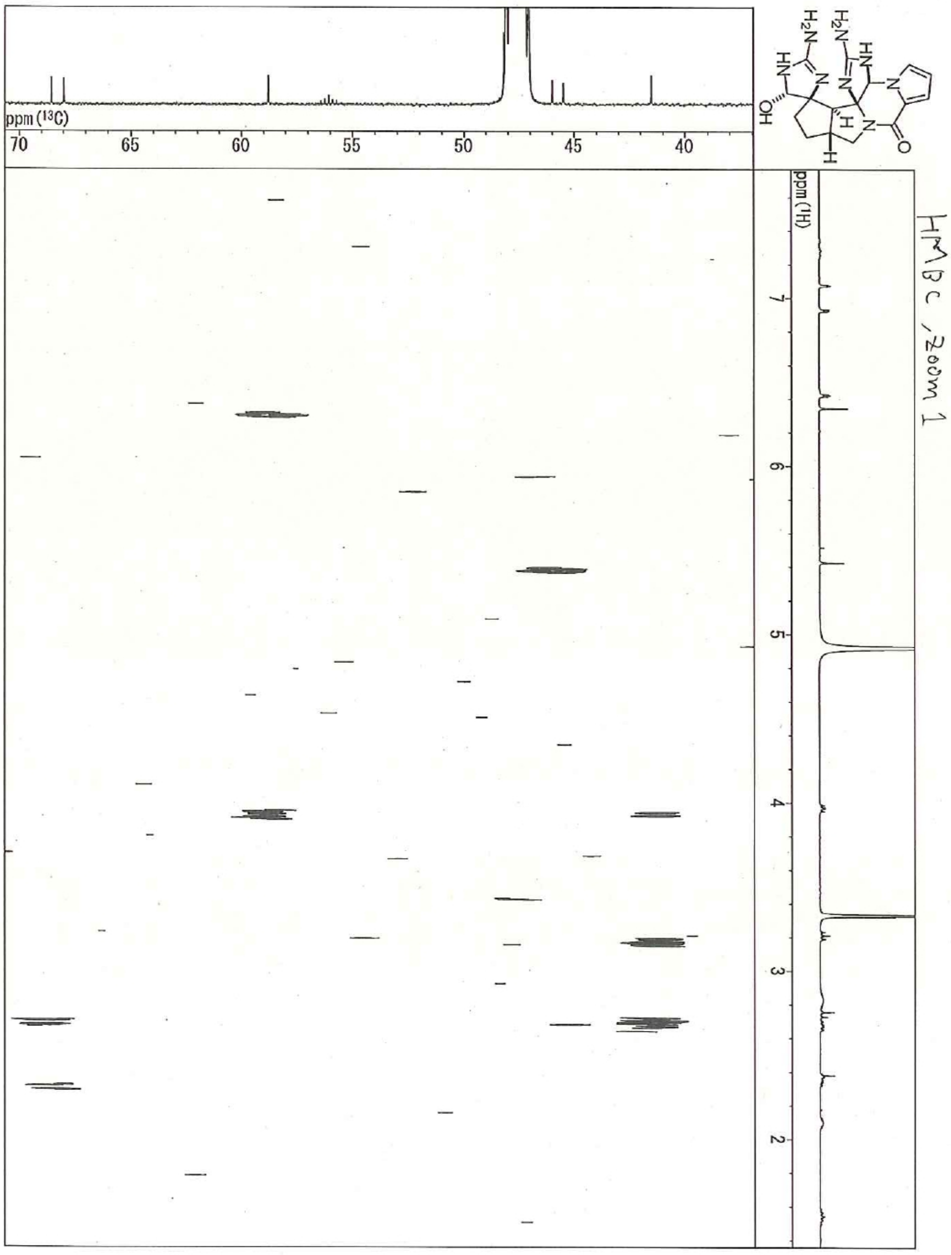

ppm (13C)
70
65
60
55
50
45
40
ppm (1H)
7
6
5
4
3
2
HMBC, zoom 1

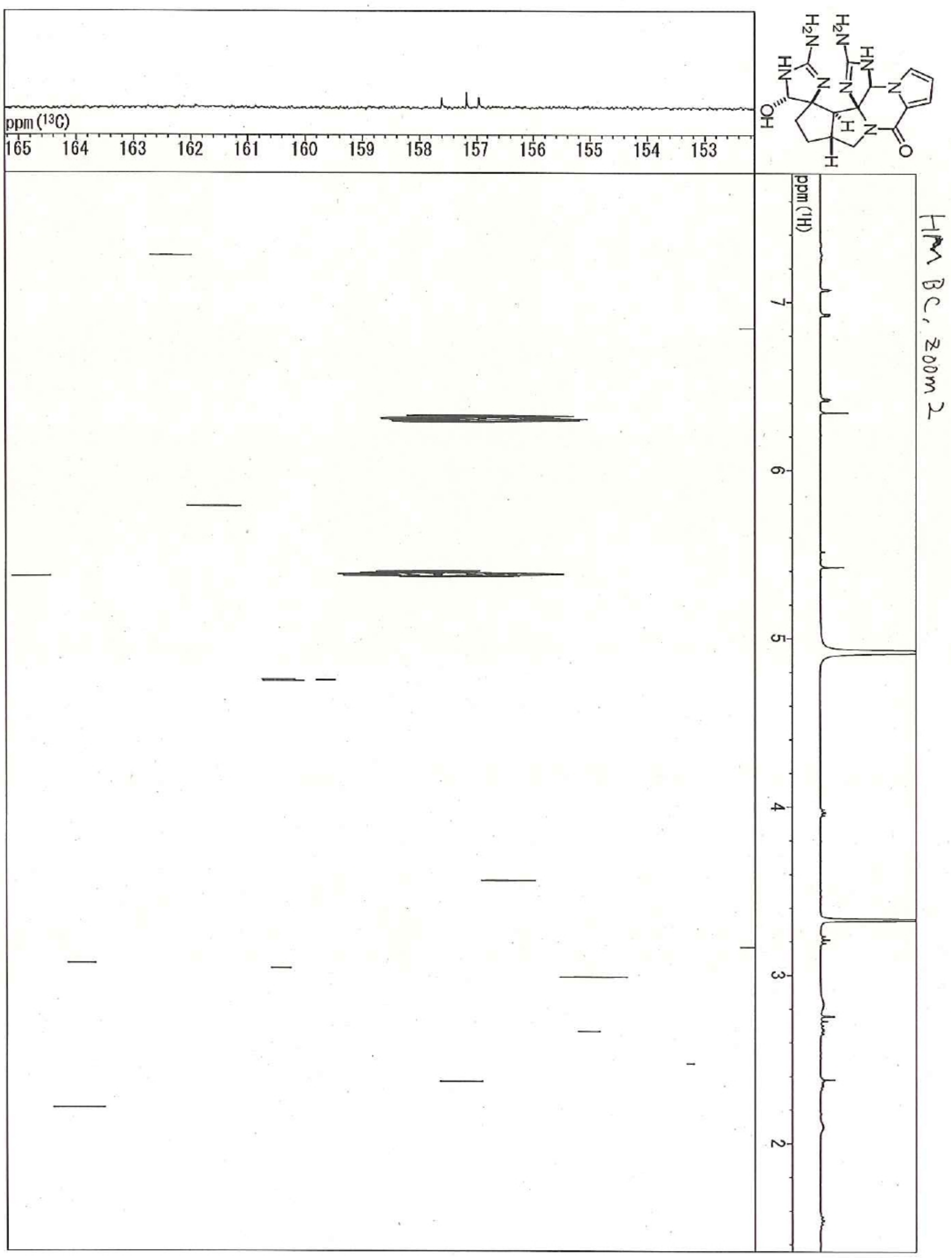
ppm (13C)
ppm (1H)
HMBC, zoom 2

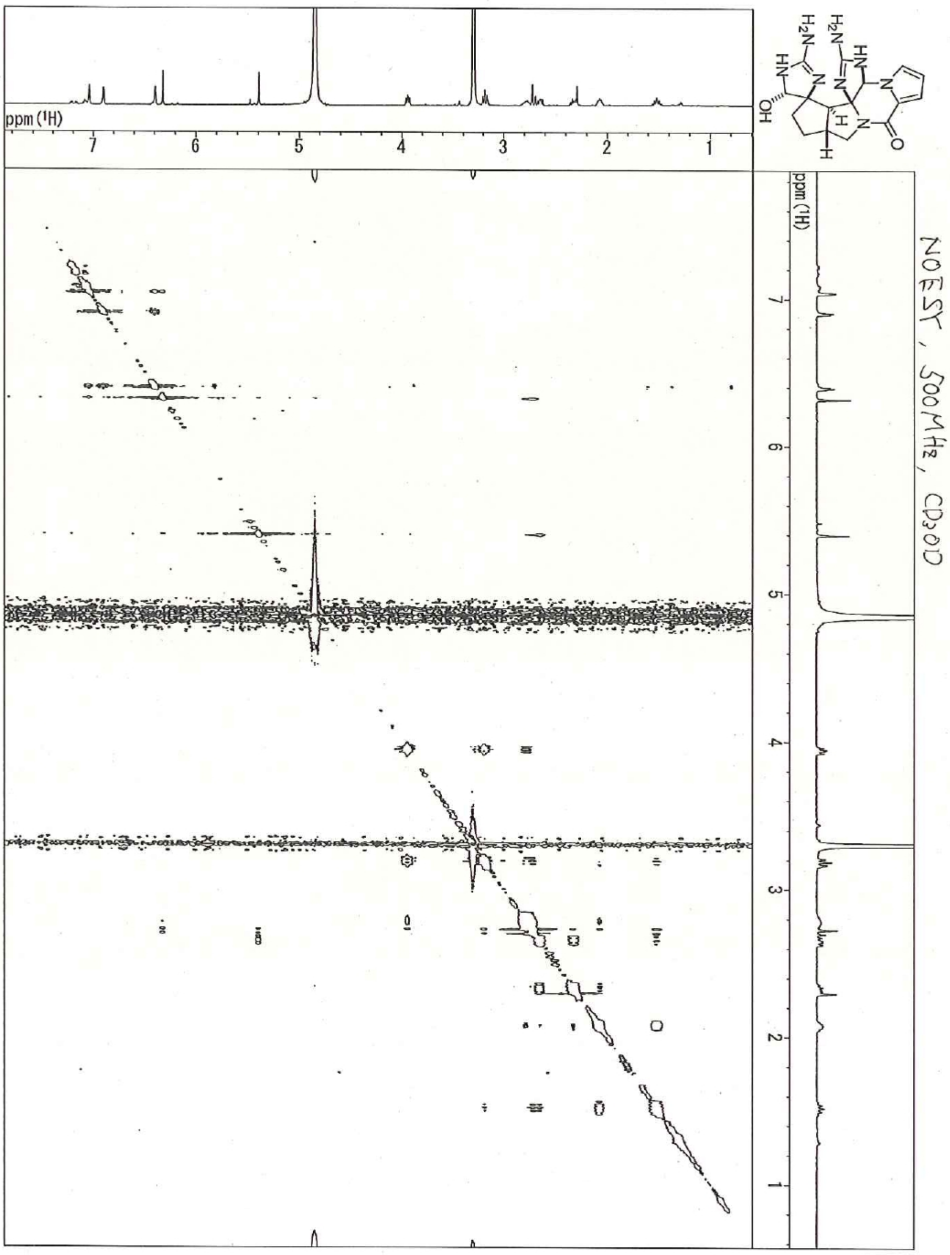
NOESY, 500MHz, CD3OD
ppm (1H)
ppm (1H)
